# Drp1 in M1 layer V GABAergic neurons orchestrates rTMS-mediated motor restoration and analgesia after spinal cord injury

**DOI:** 10.1038/s12276-026-01857-2

**Published:** 2026-10-05

**Authors:** Xiaolong Sun, Rui Zhao, Kunlong Zhang, Xingxing Feng, Xin Zhang, Xinjiang Yang, Zhenzhen Li, Ke Tian, Xin Kang, Xiaodong Lin, Ming Gao, Xu Hu, Yixing Lu, Ying Liang, Ceng Luo, Yayun Wang, Hua Yuan

**Affiliations:** 1 Department of Rehabilitation Medicine, Xijing Hospital, Fourth Military Medical University, Xi’an, China; 2 Military Medical Innovation Center, Fourth Military Medical University, Xi’an, China; 3 Department of Neurobiology, School of Basic Medicine, Fourth Military Medical University, Xi’an, China; 4 Specific Lab for Mitochondrial Plasticity Underlying Nervous System Diseases, National Demonstration Center for Experimental Basic Medicine Science Education, Fourth Military Medical University, Xi’an, China; 5 Department of Health Statistics, Fourth Military Medical University, Xi’an, China

**Keywords:** Premotor cortex, Spinal cord diseases

## Abstract

Spinal cord injury (SCI) leads to chronic motor and sensory deficits, with progressive secondary neurodegeneration posing a major therapeutic challenge. Although high-frequency repetitive transcranial magnetic stimulation (HF-rTMS) over the primary motor cortex (M1) shows therapeutic potential, its underlying cellular mechanisms remain poorly understood. This translational study first demonstrated that HF-rTMS concurrently improved motor function and alleviated neuropathic pain in retrospective clinical cohorts and a validated murine SCI model. To decipher the supraspinal mechanism, we used an integrated approach combining behavioral analyses, transmission electron microscopy and single-nucleus RNA sequencing. Transcriptomics revealed that HF-rTMS specifically rescued SCI-induced disruptions in oxidative phosphorylation and mitochondrial energy metabolism pathways within M1 GABAergic neurons. Here we pinpointed a key molecular lesion: SCI selectively downregulated the mitochondrial fission regulator Drp1 in M1 layer V GABAergic neurons, leading to dysfunctional mitochondrial dynamics and bioenergetic deficits. HF-rTMS restored Drp1 levels and mitochondrial ultrastructure specifically in M1 but not in the primary somatosensory cortex, underscoring its region-selective action. Most importantly, functional causality was established: Drp1 overexpression in M1 GABAergic neurons mimicked the therapeutic benefits of HF-rTMS, whereas Drp1 knockdown or its pharmacological inhibition completely abolished these effects. Our findings establish impaired mitochondrial dynamics in a specific cortical microcircuit as a convergent driver of multisystem deficits post SCI, and identify Drp1 as a pivotal molecular target of HF-rTMS. This work provides a novel mechanistic foundation for Drp1-directed precision therapies, highlighting the potential of rescuing cortical mitochondrial bioenergetics to halt progressive secondary damage and improve functional recovery after central nervous system injury.

**Figure:**
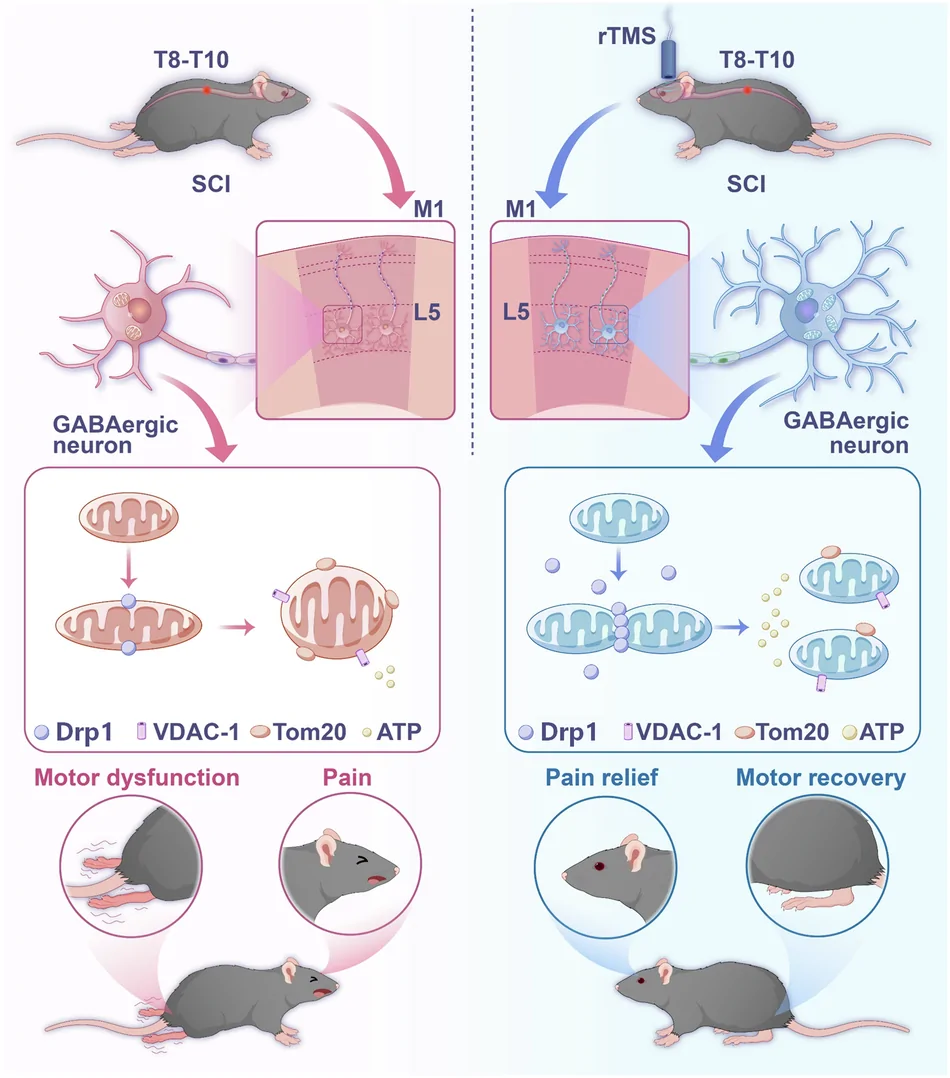
This study demonstrates that high-frequency repetitive transcranial magnetic stimulation (HF-rTMS) alleviates motor dysfunction and neuropathic pain after spinal cord injury (SCI). We show that HF-rTMS acts by upregulating the mitochondrial fission protein Drp1 specifically in layer V GABAergic neurons of the primary motor cortex. This restoration of Drp1 rescues impaired mitochondrial dynamics, normalizes associated proteins (VDAC-1 and Tom20) and recovers cellular energy (ATP) production. Consequently, rTMS reverses SCI-induced mitochondrial pathology, leading to substantial functional recovery. The therapeutic effect is dependent on Drp1, as its inhibition abolishes the benefits of HF-rTMS. Our findings reveal a precise neuromodulation mechanism targeting cortical mitochondrial homeostasis for treating SCI comorbidity.

This study demonstrates that high-frequency repetitive transcranial magnetic stimulation (HF-rTMS) alleviates motor dysfunction and neuropathic pain after spinal cord injury (SCI). We show that HF-rTMS acts by upregulating the mitochondrial fission protein Drp1 specifically in layer V GABAergic neurons of the primary motor cortex. This restoration of Drp1 rescues impaired mitochondrial dynamics, normalizes associated proteins (VDAC-1 and Tom20) and recovers cellular energy (ATP) production. Consequently, rTMS reverses SCI-induced mitochondrial pathology, leading to substantial functional recovery. The therapeutic effect is dependent on Drp1, as its inhibition abolishes the benefits of HF-rTMS. Our findings reveal a precise neuromodulation mechanism targeting cortical mitochondrial homeostasis for treating SCI comorbidity.

## HF-rTMS restores motor function by targeting Drp1 in SCI

Spinal cord injury (SCI) leads to severe motor deficits and neuropathic pain, significantly impacting quality of life. This study explores the potential of high-frequency repetitive transcranial magnetic stimulation (HF-rTMS) to address these issues by targeting the primary motor cortex (M1). Researchers used a combination of clinical data and a mouse model to investigate the effects of HF-rTMS on motor function and neuropathic pain. They discovered that HF-rTMS significantly improved both motor recovery and pain relief in patients with SCI and mice. The study identified mitochondrial dysfunction in GABAergic neurons within the M1 region as a key factor in SCI pathology. HF-rTMS was found to restore mitochondrial dynamics by upregulating the fission protein Drp1, crucial for neuronal energy metabolism. These findings suggest that HF-rTMS offers a promising noninvasive treatment for SCI, with potential for broader applications in neurological rehabilitation. This summary was initially drafted using artificial intelligence, then revised and fact-checked by the author.

## Introduction

Spinal cord injury (SCI) is a debilitating condition characterized by extensive secondary neurodegeneration that predominantly affects young and middle-aged adults, imposing severe socioeconomic consequences due to its high prevalence during peak productive years^1^. A previous study demonstrated that nearly all patients with complete SCI and the majority with incomplete SCI experience motor deficits^2^, and estimates suggest that 68% of patients with SCI suffer neuropathic pain (NP)^3^, both of which substantially reduce quality of life. Although a spontaneous repair mechanism exists after SCI, complete neurologic recovery is exceptionally uncommon, occurring in <1% of cases. Current clinical treatments, including pharmacological interventions, surgical approaches, rehabilitation and nursing care, have limited efficacy, highlighting the urgent need for new mechanistic insights to enhance rehabilitation and improve clinical outcomes.

Beyond alterations in spinal cord excitability, SCI induces extensive plastic changes in the brain, especially in cortical regions^4^. The primary motor cortex (M1) is essential for voluntary movement. After SCI, the M1 region undergoes substantial structural remodeling and electrophysiological alterations, manifesting as excitatory/inhibitory (E/I) loop imbalances and motor dysfunctions^5^. GABAergic neurons are essential for maintaining the cortical excitability balance. A recent study demonstrated that GABAergic neurons in the M1 are significantly associated with NP development^6^. Thus, the M1 region is essential in regulating both motor function and NP. Repetitive transcranial magnetic stimulation (rTMS), a noninvasive neuromodulation method, has emerged as an efficacious, nonpharmacological intervention for neurological disorders^7^. Existing studies have  demonstrated that M1-targeted high-frequency rTMS (HF-rTMS) can alleviate motor deficits^5,8^. In addition, HF-rTMS has also shown potential in mitigating NP following SCI^9–11^. Despite its therapeutic potential, the precise mechanisms by which rTMS modulates motor and sensory functions remain unclear.

Recent research has identified increased cortical reactive oxygen species (ROS) levels in SCI, contributing to oxidative stress and neuronal damage^12^. Mitochondria are crucial for neuronal function as central sites for ROS production and energy metabolism. The functionality of neuronal mitochondria is critically dependent on the balance of mitochondrial dynamics, including fission and fusion, which shape their physical form and structure. Impaired mitochondrial dynamics and function are hallmarks of many neurological disorders^13–15^. Dynamin-related protein 1 (Drp1), a key regulator of mitochondrial fission, is extensively expressed in the central nervous system^16^. Our previous study demonstrated that enhancing Drp1 expression in the dorsal horn of the spinal cord restored mitochondrial morphology and mitigated ROS accumulation, positively affecting peripheral NP^17^. We hypothesized that HF-rTMS exerts its therapeutic effects by rectifying Drp1-mediated mitochondrial dynamics in M1 GABAergic neurons, thereby alleviating the motor–pain comorbidity.

We integrated a multilevel analysis encompassing behavior, ultrastructure and omics to test this hypothesis. Retrospective clinical data first indicated that HF-rTMS can mitigate both motor deficit and NP in patients with SCI. Capitalizing on these findings, we created a murine model of SCI-induced motor deficit and NP comorbidity and examined the impact of HF-rTMS on motor function and pain-related behaviors. Furthermore, we evaluated mitochondrial morphology in the M1 region of the cerebral cortex. We investigated the complex mechanisms of HF-rTMS in motor deficit and NP treatment using single-nucleus RNA sequencing (snRNA-Seq) analysis and immunofluorescence (IF) staining, with a particular focus on the pivotal role of Drp1-mediated mitochondrial dynamics. We aimed to clarify the neural mechanism by which Drp1 alleviates motor deficit and NP in response to HF-rTMS, utilizing viral vectors and pharmacological interventions. The novel and most important finding of this study is the identification of mitochondrial dysregulation in M1 as a unifying mechanism linking motor and sensory deficits, and it establishes Drp1 as the first bidirectional mediator of rTMS efficacy in SCI recovery. This study offers a mechanism-driven paradigm for precise rehabilitation in SCI management.

## Materials and methods

### Study settings

The retrospective clinical study included motor functional and NP data of patients with SCI from January 2018 to June 2025 that were collected at the inpatient unit of the Department of Rehabilitation Medicine in Xijing Hospital, a tertiary academic medical care institution in Xi’an, People’s Republic of China. This study complies with the Declaration of Helsinki and was approved by the Institutional Review Board of the medical center (no. KY20222096-C-1). Additional informed consent was not required owing to the retrospective observational study design. Furthermore, the study was registered in the Chinese Clinical Trial Registry (ChiCTR; ChiCTR2300073824).

### Participants

Inclusion criteria included: (1) fulfilled the diagnostic criteria for SCI^18^ and were right-handed; (2) aged 18–70 years; (3) had disease duration ≤6 months from onset and presented with concomitant motor deficit and NP; and (4) received ≥4 consecutive weeks of rTMS, with assessments of motor function, pain and activities of daily living before and after the 4-week intervention. Exclusion criteria were: (1) severe neurological disorders (for example, cerebral hemorrhage and cerebral infarction) or relevant medical history; (2) co-existing conditions causing motor dysfunction (for example, limb fractures); (3) serious comorbidities such as coronary artery disease, uncontrolled hypertension or malignancy; (4) ferromagnetic implants in the head or implantation of cardiac pacemaker, absolute contraindications for TMS examination; and (5) taking drugs that may affect the TMS examination within a week, including baclofen, diazepam and other drugs^19^. Data were collected by certified rehabilitation physicians and therapists using standardized case report forms. In total, 93 eligible patients with SCI were enrolled and allocated to either the M1 region SCI + rTMS group (*n* = 40) or the SCI group (*n* = 53) according to whether they received active rTMS. In the SCI + rTMS group, stimulation was applied to the left M1 at 80% of individual resting motor threshold, delivered at 10 Hz in trains of 1.2 s followed by 3-s intertrain intervals, for a total of 1200 pulses per session. Treatment was administered once daily for four consecutive weeks.

### Clinical assessment

#### Motor score

The modified Barthel index (MBI) is a standardized assessment method used to evaluate a patient’s activities of daily living, and the total score ranges from 0 to 100. The manual muscle test (MMT) is a method of examination that determines whether the muscle strength of the examined muscle or muscle group is normal and its grade by observing the range of active limb movement and sensing the force of muscle contraction. In this study, the muscle strength of all ten key muscles of the patients was measured, with a total score of 100.

#### Pain score

The numerical rating scale (NRS) is an accurate and concise tool for pain assessment. Patients need to choose from 11 scores ranging from 0 to 10, including no pain (0), mild pain (1–3), moderate pain (4–6) and severe pain (7–10).

### Animals

Adult male C57BL/6J mice weighing 20 ± 2.5 g on average were provided by the Experimental Animal Center of the Fourth Military Medical University, Xi’an, Shaanxi, China. The animals were housed under a 12-h light–dark cycle (8:00 to 20:00), with unrestricted access to food and water. Male mice were used throughout the study to control for sex-related differences. All mice were randomly assigned to the groups. All animal protocols were conducted in accordance with the procedures approved by the Animal Ethical and Welfare Committee of the Fourth Military Medical University (permission number IACUC-20241244) and followed our institutional guidelines for the use of laboratory animals.

### Surgical procedures of SCI model

A model of SCI in mice was induced as described previously^20^. Mice were given pentobarbital anesthesia (50 mg/kg, intraperitoneal (i.p.)) and a laminectomy was performed at T9–T11 to expose spinal cord segments. An acute-compression SCI was inflicted in mice at T10 by using a micro-vascular clip (with 30 *g* force, ST2 64-0195, Harvard Apparatus) under visualization using a stereozoom microscope (SZ 60, Olympus). The dura and spinal nerve roots were not disturbed, and the clip was left in place for 30 s. The clip was removed, and the incisions were closed. Sham mice received laminectomy without compression. Bladders were manually expressed twice daily until reflex bladder function was established (7–14 days after SCI).

### rTMS

rTMS was delivered using a stimulator (YRD CCY-II, YIRUIDE) equipped with a 2-cm diameter rodent coil^21^. Mice received one HF-rTMS session daily, starting on day 3 post SCI^22^. We used stimulation with 40% intensity of machine output and a frequency setting of 20 Hz, a stimulation time of 2 s, a time interval of 10 s, a total number of 1,600 pulses and a total stimulation time of 8 min per mouse per day. The SCI group (Sham rTMS) received a similar treatment, except that the TMS coil was positioned parallel to the animal’s head, which resulted in the exposure of mice to the acoustic artifact of rTMS.

### Behavioral assays

#### BMS

For mice, the Basso Mouse Scale (BMS) open-field test was used^23^. In brief, the BMS uses a nine-point scale that provides a gross indication of locomotor ability and determines the phases of locomotor recovery and features of locomotion. The score for each animal was recorded as the average of the left and right hind paws to obtain one BMS score per mouse, and then, the mean of the group was calculated.

#### Mechanical allodynia

Baseline behavioral assessments were conducted within 2 weeks after SCI to identify the development of NP. Using von Frey monofilaments, we assessed the paw-withdrawal mechanical threshold (PWMT) method to assess the hypersensitive response of mice to mechanical stimuli. To simulate the pain situation of SCI-NP patients in the clinic, both hind paws of SCI mice were tested, and the side with more severe pain symptoms was selected for statistical analysis.

#### Thermal hyperalgesia

A thermal testing apparatus (PL-200, Chengdu Techman Software) was used to examine the thermal sensitivity of the mice^24^. The paw-withdrawal thermal latency (PWTL), defined as the time from irradiation onset to paw withdrawal, was recorded. The PWTL data were recorded three times, and the average time was utilized for statistical analysis. A minimum interval of 5 min was maintained between each set of hind paw tests.

#### Gait analysis

Gait analysis was performed using the CatWalk XT system (Noldus)^25^. The mouse was placed on the open end of an enclosed glass platform and walked across the glass floor voluntarily; a high-speed camera underneath the device captured images of each paw and transmitted the data to gait analysis software (CatWalk XT, version 10.6; Noldus).

### Stereotaxic surgery

For viral vector delivery, we used plasmid adeno-associated viruses (pAAVs) under the control of the GAD67 promoter to achieve cell-specific manipulation of Drp1 in GABAergic neurons. To overexpress or knockdown Drp1 specifically in GABAergic neurons, we used pAAV-GAD67-EGFP-P2A-*Dnm1l*-3×FLAG-tWPA (8.70 × 10^12^ genome copies (GC)/ml) or pAAV-GAD67-mCherry-3×FLAG-miR30shRNA(*Dnm1l*)-WPRE (3.66 × 10^12^ GC/ml), respectively. Both viruses were produced by Heyuan Biotechnology (OBIO). Mice were anesthetized with pentobarbital and placed in a stereotaxic apparatus (RWD 69100). The coordinates used were based on the Paxinos and Franklin’s Mouse Brain Atlas (Fourth Edition) for the bilateral M1 region: anteroposterior 0.25 mm, mediolateral ±1.25 mm, dorsoventral −0.60 mm (from the brain surface)^26^. A microsyringe (RWD 79013) was used to inject adeno-associated virus (AAV) with a target injection capacity of 400 nl (1.0 × 10^12^ GC/ml) and an injection speed of 40 nl/min, with the same dose of PBS serving as a vehicle control. The needle was left in the brain for a further 10 min before it was removed. The wound was sutured with intermittent sutures, and iodine disinfectant was applied to the closed wound.

### Analysis of mitochondrial function

Brain tissue from the M1 region was homogenized and centrifuged, and the supernatant was aspirated. The ATP content in the supernatant was analyzed by an ATP analysis kit (S0027, Beyotime). The ROS content in the supernatant was analyzed using a ROS kit (S0033S, Beyotime). The mitochondrial membrane potential in the supernatant was assessed using a JC-1 test kit (C2006, Beyotime).

### Characterization of GABAergic neurons in TEM

Samples were obtained from multiple ultrathin sections of 6 biologically independent mice per group. The identification of GABAergic neurons under transmission electron microscopy (TEM) mainly relies on their unique ultrastructural characteristics, including synaptic morphology and vesicle types^27^. GABAergic synapses are classified as symmetric (Gray II-type), characterized by a thin postsynaptic density (PSD) of similar thickness to the presynaptic membrane and predominantly pleomorphic synaptic vesicles. This contrasts with glutamatergic synapses, which are asymmetric (Gray I-type) and feature a prominent, thickened PSD and round synaptic vesicles. These distinct synaptic morphologies served as the primary ultrastructural signature for distinguishing GABAergic from glutamatergic neurons in layer V of the M1 cortex.

### Morphometric analysis

The size of each mitochondrion was obtained by drawing the electron microscopy sagittal profile of the mitochondria^17^. The circularity of mitochondria was determined according to the method previously described^26^, that is, circularity = 4πarea/perimeter^2^. The number of mitochondria was hand-counted in the M1 region of each image to get the total number of mitochondria, and then the density of the mitochondria was calculated.

### Drug treatment

Mdivi-1 (MCE, HY-15886) was prepared by dissolving in DMSO and was intraperitoneally administered at 10 mg/kg^17^ or equal volume of vehicle DMSO at 1 h before weekly behavioral assays respectively.

### Statistical analysis

The sample size estimation was based on pilot study data of PWMT (NP) and BMS (motor function). After rTMS treatment, the average value of PWMT in SCI mice changed from 0.07 (±0.01) to 0.38 (±0.02), and 3.83 (±0.31) to 5.33 (±0.21) for BMS. To achieve a desired power of 80% and with a type 1 error set at 0.05, we calculated that a minimum of six mice per group would be sufficient to detect biologically meaningful differences. Mice were randomized using a random-number table, and investigators were blinded. For categorical variables, chi-squared or Fisher’s exact test was used. Normality and variance were assessed using the Shapiro–Wilk and Levene tests for continuous variables. Normally distributed data were analyzed with unpaired *t*-test (Welch’s correction), one-way or repeated-measures ANOVA, all followed by Bonferroni post hoc tests. For nonparametric tests, Dunn’s post hoc test was used following the Kruskal–Wallis test. Data are expressed as mean ± standard error of the mean, and non-normal data are represented by the median and interquartile range (IQR). Values of *P* < 0.05 were considered statistically significant.

## Results

### HF-rTMS concurrently improved motor function and attenuated NP in patients with SCI

**Fig. 1:**
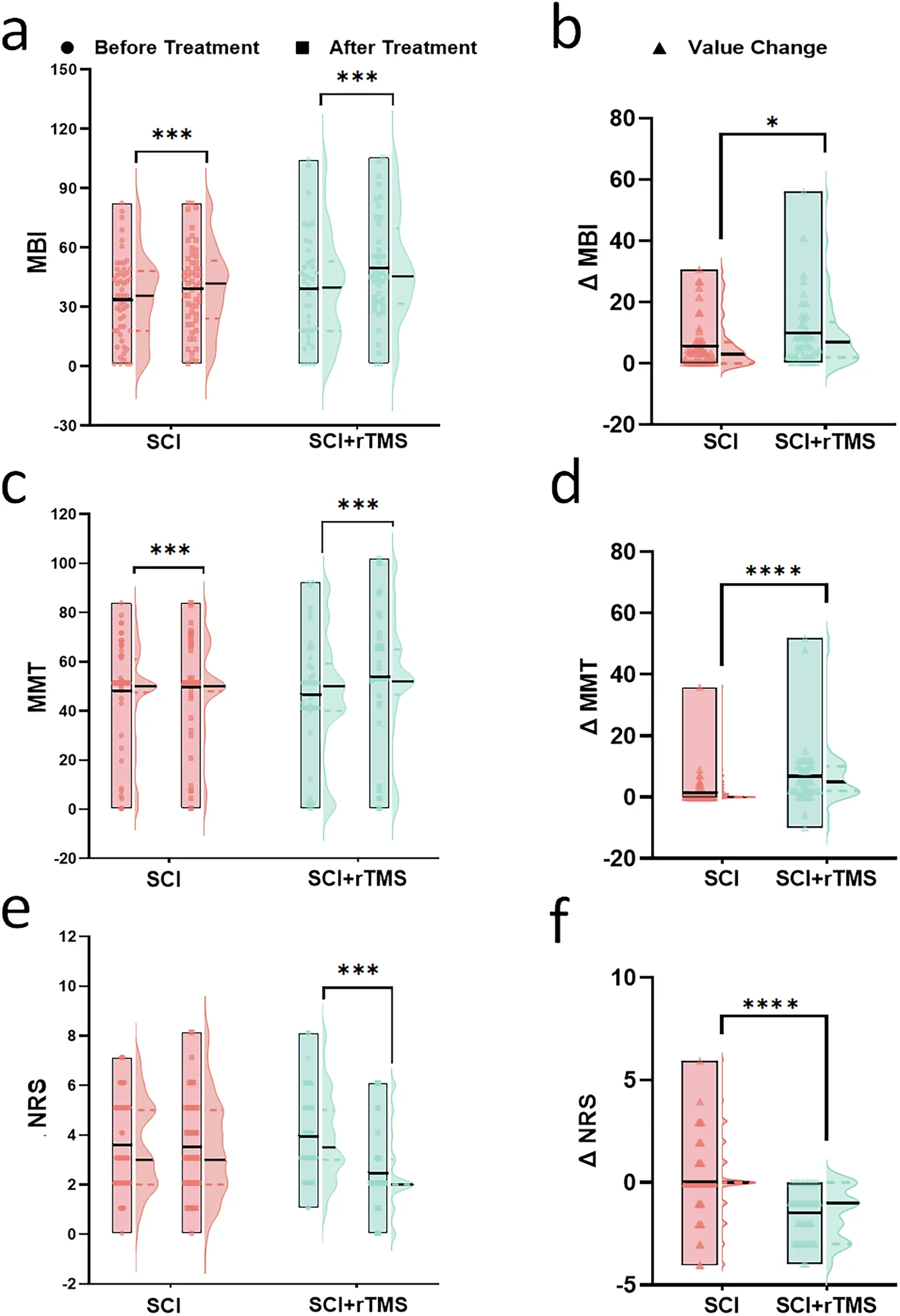
Motor and sensory assessment results of patients with SCI after HF-rTMS. **a**, **b** The MBI value results (**a**) and the changes in MBI (**b**) of patients in the SCI and SCI + rTMS groups before and after 4-week HF-rTMS treatment. **c**, **d** The MMT value results (**c**) and the changes in MMT (**d**) of patients in the SCI and SCI + rTMS groups before and after 4-week HF-rTMS treatment. **e**, **f** The NRS value results (**e**) and the changes in NRS (**f**) of patients in the SCI and SCI + rTMS groups before and after 4-week HF-rTMS treatment. Data are presented as box plots (left) showing the mean and kernel probability density and violin plots (right) showing the median (solid line) and IQR (dashed lines). *n* = 53 patients in SCI, *n* = 40 patients in SCI + rTMS. rTMS refers to HF-rTMS. *P < 0.05, ***P < 0.001 and ****P < 0.0001.

Although HF-rTMS has been separately reported to enhance motor recovery or relieve NP after SCI, the two symptom domains are rarely examined in tandem, leaving unresolved whether HF-rTMS can benefit the frequent motor–pain comorbidity characteristic of SCI. To address this gap, we retrospectively enrolled patients with SCI admitted between January 2018 and June 2025 and allocated them to either the SCI + rTMS group (*n* = 40) or the SCI group (*n* = 53) according to whether they received HF-rTMS. The demographic data and basic information did not differ between groups (Supplementary Table 1). After 4 weeks, both groups exhibited significant improvements in the MBI compared with baseline, with the magnitude of improvement significantly greater in the SCI + rTMS group (Fig. 1a, b). MMT scores showed a similar pattern, indicating that HF-rTMS conferred superior motor recovery (Fig. 1c, d). Notably, only the SCI + rTMS group demonstrated a significant reduction in the NRS for pain (Fig. 1e), and the extent of this reduction was significantly larger than in the SCI group (Fig. 1f). Collectively, these data suggest that HF-rTMS can simultaneously ameliorate motor deficits and NP after SCI.

### HF-rTMS alleviates the comorbidity of motor deficit and NP in SCI mice

The pathophysiology of SCI is complex, and understanding the underlying pathophysiological mechanisms entails the development of preclinical models that closely replicate human clinical motor deficit and NP symptoms. Here, we focused on the acute-compression SCI model in mice^20^. The compressed spinal cord tissue presented obvious congestion, edema and scattered petechiae, and this model induces persistent motor dysfunction and pain-related behaviors (Supplementary Fig. 1), mirroring the clinical comorbidity. Histopathological assessment via hematoxylin and eosin staining at the injury epicenter confirmed hemorrhage, substantial tissue disruption, neuronal loss and early tissue vacuolation in the acute (1-day) and subacute (3-day) phases post SCI and Nissl staining of both sagittal and coronal sections confirmed marked neuronal loss within the lesion core, with diminished Nissl body staining and nuclear pyknosis in surviving neurons adjacent to the injury site (Supplementary Fig. 2a–c). To evaluate tissue damage and white matter sparing, we performed triple-IF co-staining for neurofilament heavy chain (NF200, marking axons), glial fibrillary acidic protein (GFAP, delineating reactive astrocytes and glial scar), and ionized calcium-binding adaptor molecule 1 (Iba1, marking microglia/macrophages) at 3 days post SCI, following established protocols for SCI histopathology. The glial scar formed a clear boundary between the lesion core and the adjacent spared tissue, with axonal interruption evident at the rostral and caudal margins of the lesion (Supplementary Fig. 2d). Coronal sections at the injury epicenter showed a central lesion core surrounded by a GFAP^+^ glial scar ring with abundant Iba1^+^ cells. Critically, NF200 staining revealed a peripheral rim of preserved axons in the dorsal columns, lateral funiculi and ventral funiculi, confirming substantial but incomplete axonal loss (Supplementary Fig. 2e). This acute and subacute pathology validated the severity of the compression injury.

The BMS was utilized to assess alterations in motor function after SCI. The BMS scores (primarily indicating motor function recovery) and subscores (primarily indicating the coordination of hind paws) revealed profound deficits in SCI mice immediately after injury. While a degree of spontaneous recovery occurred, BMS scores and subscores remained significantly lower in SCI mice compared with Sham mice throughout the 5-week period, confirming sustained motor impairment (Supplementary Fig. 1c). We evaluated the PWMT changes in the hind paw of SCI mice using the von Frey test, the gold standard for algesimetry. PWMT scores decreased markedly after the mice regained autonomous hind paw movement and remained significantly lower than those of Sham mice for 5 weeks (Supplementary Fig. 1d). In addition, the PWTL exhibited a similar downward trend, indicating that SCI caused a significant reduction in heat pain threshold (Supplementary Fig. 1e). The examination of both hind paws revealed symmetrical pain responses, excluding unilateral bias (Supplementary Fig. 1f). To obtain a more integrated and objective assessment, we specifically implemented the CatWalk gait analysis, which revealed profound alterations in the gait pattern of SCI mice (Supplementary Fig. 1g). Compared with the Sham group, the regularity index (Supplementary Fig. 1h) and body speed (Supplementary Fig. 1i) in the SCI group were significantly reduced, suggesting that the SCI mice exhibited motor abnormalities, consistent with the BMS results. Furthermore, parameters indicative of pain and weight-bearing were altered. The maximum contact mean intensity was significantly increased in the front paws and decreased in the hind paws. Concurrently, the standing duration and maximum contact area of the hind paws were markedly reduced (Supplementary Fig. 1j–l). This pattern signifies a redistribution of load away from painful hind limbs, providing a quantifiable behavioral correlate of NP. Together, these data establish that our mouse model faithfully recapitulates the comorbid motor–pain phenotype observed in patients with SCI.

**Fig. 2:**
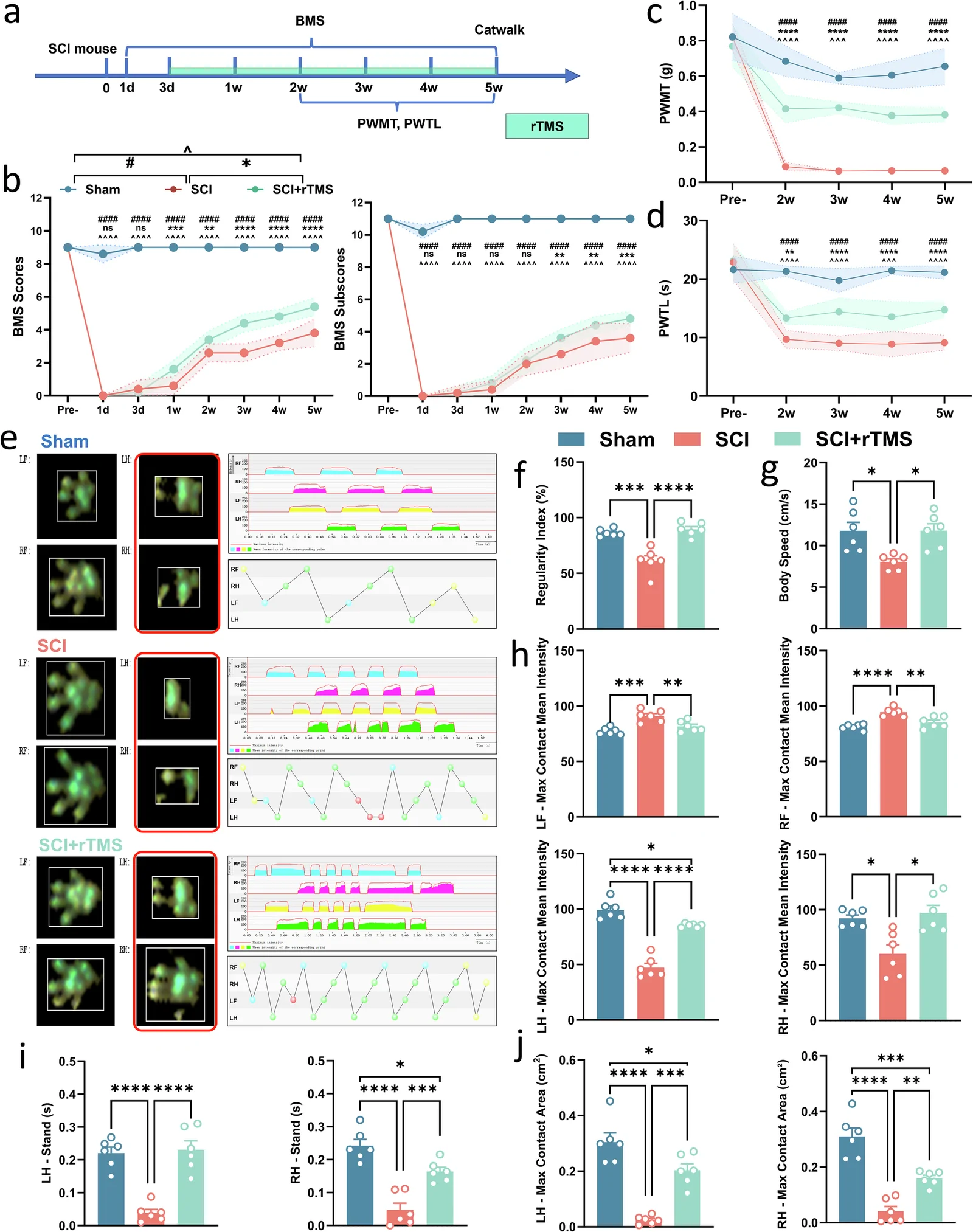
HF-rTMS can improve motor deficit and NP caused by SCI. **a** Schematic representation of the experimental paradigm. **b** BMS scores evaluating locomotor function in Sham, SCI and SCI + rTMS mice at various time points post SCI. **c**, **d** Assessment of mechanical allodynia (**c**) and thermal hyperalgesia (**d**) in Sham, SCI and SCI + rTMS mice. Data are presented as PWMT and PWTL, respectively. **e** The typical mouse paw print in CatWalk, where the red box is the hind paw print. **f**–**j** Quantification of CatWalk gait analysis in Sham, SCI and SCI + rTMS mice at 5 weeks post SCI, as measured by the regularity index (**f**), body speed (**g**), the maximum contact mean intensity of paw prints (**h**), the stand time (**i**), and the maximum paw contact area (**j**) of hind paw prints. *n* = 6 mice per group in all. Line graphs (**b**–**d**) use # (Sham versus SCI), ^ (Sham versus SCI + rTMS), * (SCI versus SCI + rTMS). Graded symbols correspond to *P* < 0.05, 0.01, 0.001, 0.0001. ns, not significant. rTMS refers to HF-rTMS. *P < 0.05, **P < 0.01, ***P < 0.001 and ****P < 0.0001.

We next investigated the therapeutic effect of HF-rTMS in this model. HF-rTMS treatment significantly accelerated the rate of motor recovery. BMS scores and subscores in the SCI + rTMS group increased more rapidly and reached significantly higher plateaus compared with the untreated SCI group from week 1 onwards (Fig. 2a, b). In parallel, HF-rTMS produced a potent analgesic effect, significantly reversing both mechanical allodynia and thermal hyperalgesia (Fig. 2c, d). Furthermore, the CatWalk gait analysis revealed that 5-week HF-rTMS significantly restored the gait of SCI mice (Fig. 2e). Specifically, the regularity index and body speed were significantly improved (Fig. 2f, g), indicating better coordination and mobility. Crucially, the pain-associated gait parameters were also reversed (Fig. 2h–j), indicating restored weight-bearing capacity. Collectively, these data validate the model’s comorbid motor–pain phenotype and demonstrate that HF-rTMS affords rapid, sustained dual symptomatic relief.

### snRNA-Seq reveals the effect of HF-rTMS on M1 cells and the regulation of mitochondrial function after SCI

**Fig. 3:**
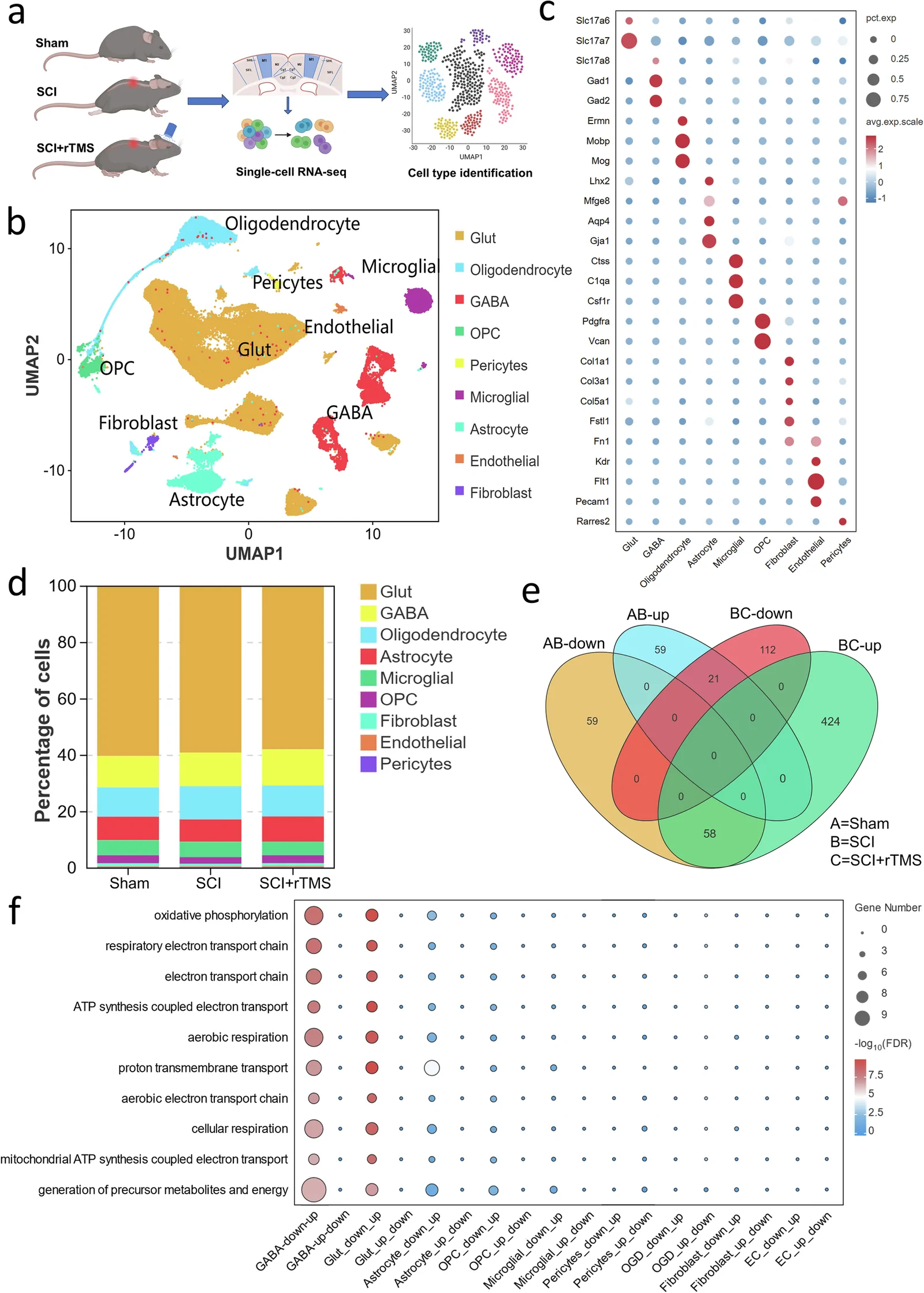
Results of snRNA-Seq in M1 region of mice. **a** The snRNA-Seq of mouse M1 region. **b** The UMAP clustering of ~90,000 snRNA-Seq libraries from three groups of mice identifies nine mega-clusters in the M1 region. **c** Different clusters were screened according to specific markers; a dot plot showing the expression level (color scale) and the percent of cells expressing (dot size) marker genes across cell types. **d** The percentage of different cell distribution in each group of mice. **e** A Venn diagram shows the number of genes significantly different in SCI versus Sham and/or in SCI + rTMS versus SCI in M1 region (*n* = 3 mice per group), with GABAergic neurons as an example. A, Sham; B, SCI; C, SCI + rTMS. **f** The top ten biological processes of GO analysis with emphasis on mitochondrial energy metabolism-related processes; a dot plot showing the expression level (color scale) and the number of genes (dot size) in different biological processes.

We conducted snRNA-Seq analysis to elucidate the complex and dynamic interactions among cells within the M1 region influenced by SCI and HF-rTMS. Our investigation commenced with the analysis of approximately 90,000 cells procured from the M1 region of Sham, SCI and SCI + rTMS groups (Fig. 3a). Utilizing relevant biomarkers and uniform manifold approximation and projection clustering, nine major cell types were identified, including neuronal, glial, endothelial and fibroblast populations (Fig. 3b, c). The subsequent quantification of cellular proportions revealed glutamatergic neurons as the dominant population, followed by GABAergic neurons and oligodendrocytes; importantly, the proportional distribution of cell types in each group remained unchanged (Fig. 3d). Comparative analyses of differentially expressed genes across these cell types were then undertaken to delineate the counteracting influence of rTMS on SCI-driven transcriptional alterations. The Venn analysis identified two key gene sets: (1) genes upregulated by SCI but downregulated by HF-rTMS (AB-up and BC-down) and (2) genes downregulated by SCI but upregulated by HF-rTMS (AB-down and BC-up) (Fig. 3e and Supplementary Fig. 3a–i). Subsequently, Gene Ontology (GO) enrichment analysis was performed on reversed gene sets from all M1 cells to identify the biological processes most prominently affected by SCI and reversed by HF-rTMS. This cell-type-resolved analysis revealed distinct reversing pathway signatures (Supplementary Fig. 3j, k and Supplementary Tables 2–10). Microglia, astrocytes and oligodendrocyte lineage cells showed changes related to synaptic transmission, cell cycle, glycolysis and nervous system development (Supplementary Tables 2, 7 and 8). By contrast, mitochondrial energy metabolism was the dominant pathway in GABAergic and glutamatergic neurons (Supplementary Tables 5 and 6). Given that the primary endpoints of this study are motor function recovery and NP alleviation—two processes that critically depend on the output of M1 cortical neurons and their exceptionally high bioenergetic demands—we focused our main figure presentation and subsequent functional investigation on mitochondrial energy metabolism pathways (Fig. 3f). This enrichment was particularly pronounced within neuronal populations. Terms such as ‘oxidative phosphorylation’, ‘ATP synthesis coupled electron transport’, ‘aerobic respiration’ and ‘proton transmembrane transport’ were prominently featured. Further examination showed that the number of reversing genes and the statistical significance of enrichment were strongest in GABAergic neurons, followed by glutamatergic neurons, compared with glial cells such as astrocytes and oligodendrocyte precursor cells. This bioinformatic finding strongly suggests that the pathophysiological impact of SCI on the M1 cortex and the therapeutic action of HF-rTMS converge on the regulation of mitochondrial bioenergetic capacity within specific neuronal circuits, with GABAergic and glutamatergic neurons appearing especially pivotal.

### SCI-induced mitochondrial morphological alteration and dysfunction in GABAergic neurons within layer V of the M1 region

**Fig. 4:**
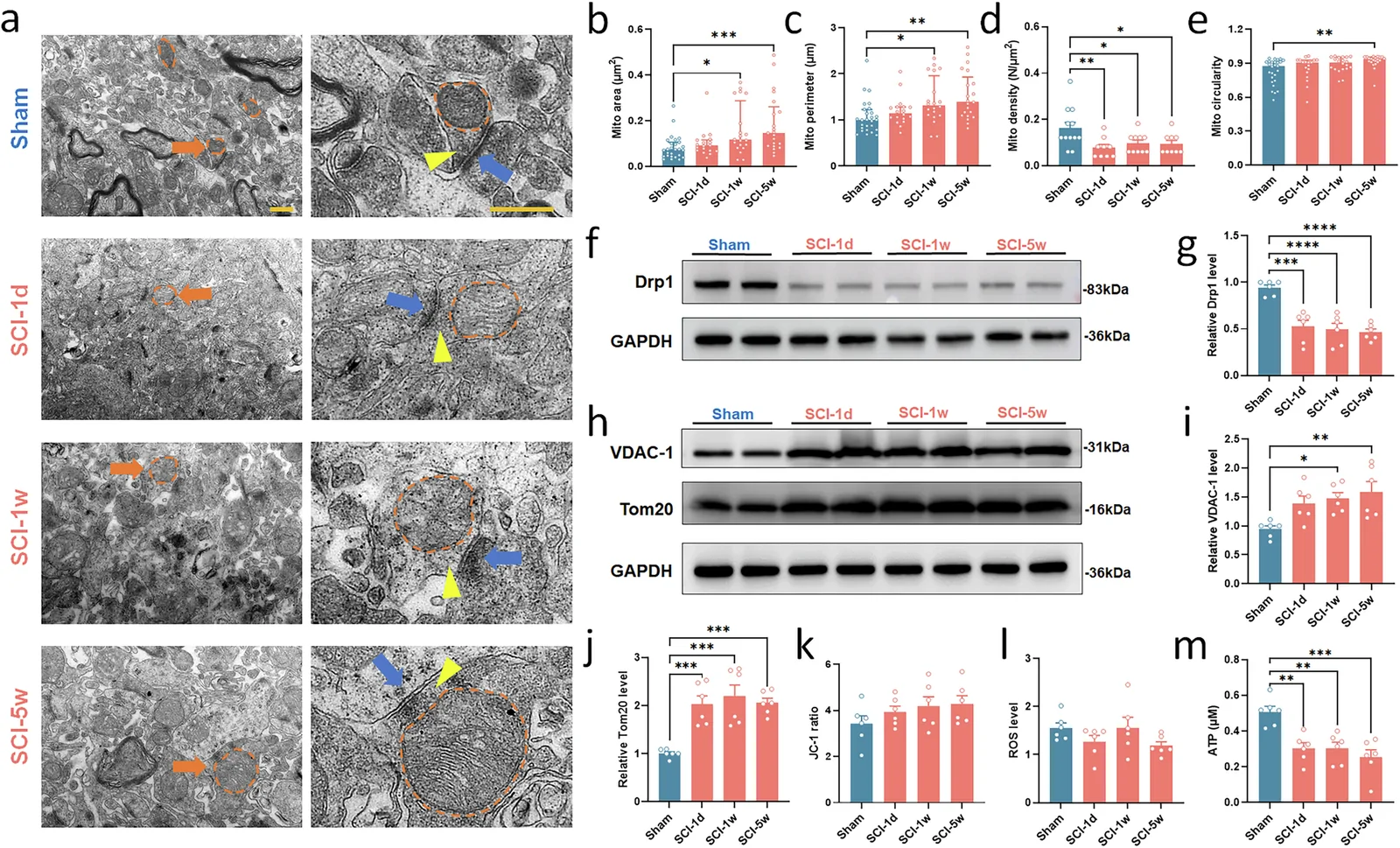
Decreased Drp1 after SCI leads to abnormal mitochondrial division and dysfunction. **a**–**e** TEM images of mitochondria in the M1 region of Sham and SCI mice (scale bars, 500 nm) and quantified by the area (**b**) perimeter (**c**), density (**d**) and circularity (**e**) of mitochondria in Sham (*n* = 27), SCI-1d (*n* = 18), SCI-1w (*n* = 20) and SCI-5w (*n* = 21) groups. The orange arrows and dotted lines identify mitochondria, the blue arrows indicate Gray II-type symmetric synapses (characterized by equally thickened pre- and postsynaptic membranes, a hallmark of GABAergic synapses), and the golden triangles identify synaptic vesicles. **f** Western blot analysis of Drp1 protein expression in the M1 region of Sham and SCI-1d, SCI-1w and SCI-5w post SCI. **g** Quantification of Drp1 protein levels normalized to GAPDH (*n* = 6 per group). **h**–**j** Western blot analysis of VDAC-1 and Tom20 protein expression in the M1 region (**h**), and quantification of VDAC-1 (**i**) and Tom20 (**j**) protein levels normalized to GAPDH (*n* = 6 per group). **k**–**m** quantification of mitochondrial function (*n* = 6 per group), including JC-1 ratio (**k**) ROS level (**l**) and ATP (**m**). *P < 0.05, **P < 0.01, ***P < 0.001 and ****P < 0.0001.

Given this cell-type-specific distribution pattern, we hypothesized that mitochondrial bioenergetic dysfunction in neurons constitutes a core pathological feature of SCI and a major target of HF-rTMS treatment. Therefore, we next focused on the mechanism underlying changes in mitochondrial function of GABAergic and glutamatergic neurons. Mitochondria are highly active organelles with continuous cycles of fusion and fission, essential for mitochondrial function^28^. To accurately characterize the changes in mitochondrial morphology at various periods after SCI, we selected the results of SCI mice at 1 day (SCI-1d), 1 week (SCI-1w) and 5 weeks (SCI-5w) post SCI, (corresponding to the acute, subacute and chronic stages of patients with SCI, respectively)^29^ and used TEM to examine the structural modifications of neuronal mitochondria. Previous studies have demonstrated that neurons in layer V of M1 are pivotal in movement execution and pain modulation^6,21,30^. GABAergic neurons exhibit symmetric synapses (Gray II-type), characterized by equally thickened pre- and postsynaptic membranes due to the absence of pronounced PSD thickening, in contrast to the asymmetric synapses (Gray I-type) in glutamatergic neurons^27^. Leveraging this distinct ultrastructural trait, we examined the impact of SCI on mitochondrial morphology of these two neuronal subtypes of the M1 region (Fig. 4a and Supplementary Fig. 4a). The quantitative ultrastructural analysis revealed that, compared with Sham mice, mitochondrial remodeling was evident in GABAergic neurons within layer V of the M1 in SCI mice (Fig. 4b–e). SCI mice exhibited progressive mitochondrial enlargement, evidenced by the increase in size (Fig. 4b) and elongation in perimeter (Fig. 4c), alongside a decrease in neuronal mitochondrial density (Fig. 4d). This morphological shift continued throughout the 5-week post-injury period, indicating the sustained impairment of the Drp1-dependent mitochondrial fission mechanism. Moreover, the results revealed that the circularity of mitochondria, which is significantly associated with mitotoxicity and neurotoxicity^26^, increased significantly after SCI-5w (Fig. 4e), suggesting compromised mitochondrial quality control mechanisms after SCI. By contrast, glutamatergic neurons showed a more modest and delayed response, with significant increases in area and perimeter after SCI-5w and no significant changes in density or circularity (Supplementary Fig. 4b–e). This indicates a particular vulnerability of GABAergic interneurons to SCI-induced mitochondrial dynamic imbalance.

Drp1 is the master regulator of mitochondrial fission and involved in mitochondrial quality control^31^. Western blot analysis revealed a rapid and sustained decrease in Drp1 protein expression specifically within the M1 cortex of SCI mice, which persisted for at least 5 weeks (Fig. 4f, g). This decrease was more pronounced and enduring in M1 compared with the spinal cord tissue below the injury (Supplementary Fig. 5a, b). Mitochondrial fusion is the converse process of fission. Mitofusin 1 and 2 (Mfn1 and Mfn2) proteins on the outer mitochondrial membrane are responsible for the fusion process^32,33^. The results revealed that no significant difference was observed in Mfn1 or Mfn2 expression in the M1 region at any stage after SCI (Supplementary Fig. 5c, d).

Compromised mitochondrial integrity promotes mitochondrial autophagy, an essential mechanism for maintaining cellular homeostasis^34^. We detected a markedly elevated protein abundance of voltage-dependent anion channel 1 (VDAC-1) and translocase of the outer mitochondrial membrane 20 (Tom20) after SCI (Fig. 4h–j). These two outer mitochondrial membrane proteins are indispensable for initiating autophagic cascades and sustaining intact, functionally efficient mitochondrial networks; their upregulation therefore indicates massive buildup of damaged mitochondria stemming from defective mitochondrial clearance^35^. Functional assessment confirmed a state of bioenergetic crisis. While JC-1 ratio and ROS levels were not significantly altered, the ATP content in the M1 tissue was substantially depleted at both 1 and 5 weeks post SCI (Fig. 4k–m). This decrease in ATP indicated a disruption in the mitochondrial energy metabolism, highlighting a decline in the mitochondria’s ability to meet the cell’s energy demands. In summary, SCI induces a Drp1-deficient state in M1, leading to aberrant mitochondrial morphology and impaired mitochondrial function. These insights highlight the significance of restoring mitochondrial dynamic balance and functionality in SCI.

### HF-rTMS induces upregulation of Drp1 in GABAergic neurons in layer V of M1

**Fig. 5:**
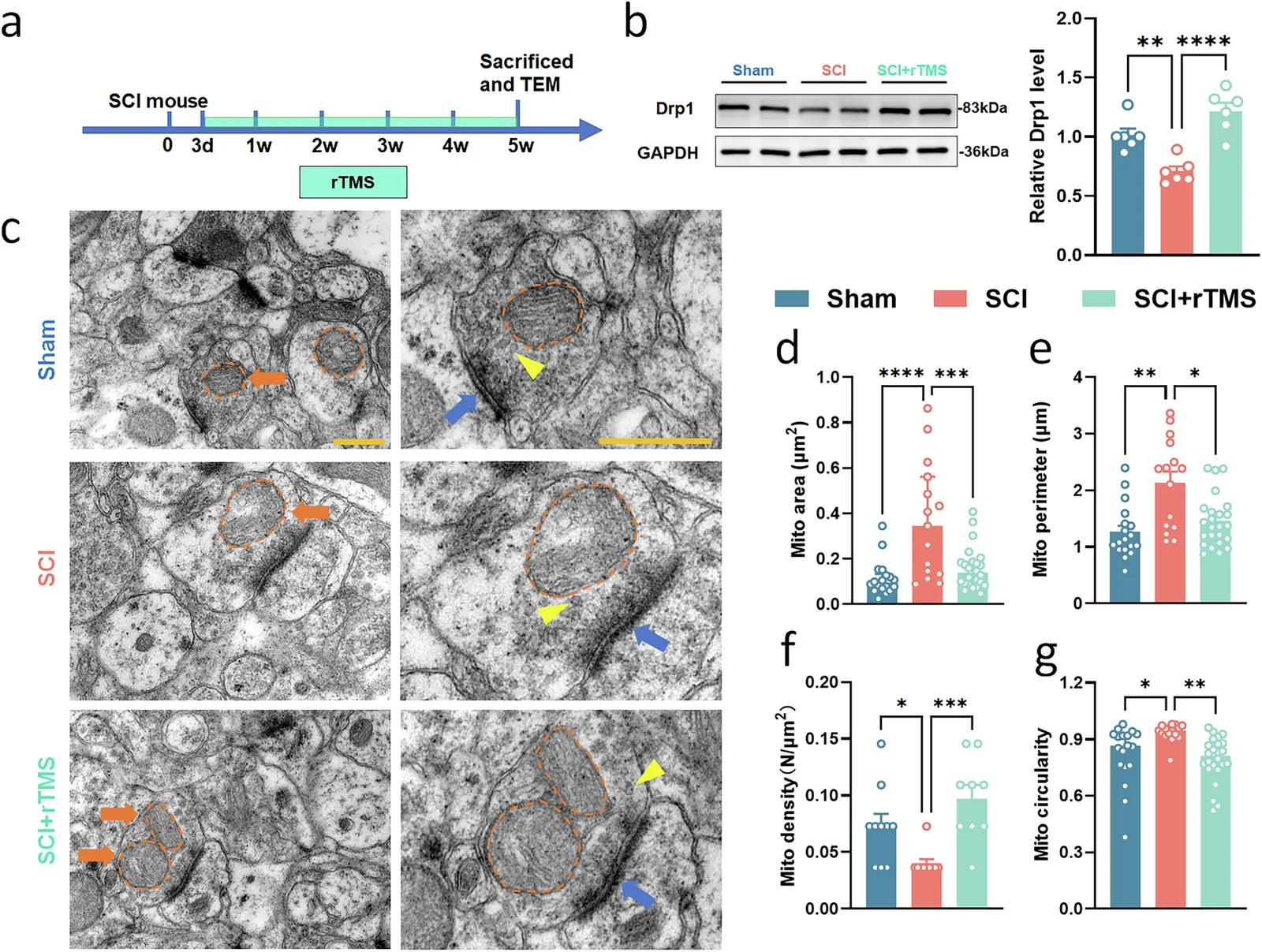
HF-rTMS can improve mitochondrial division abnormalities in M1 region. **a** Schematic representation of the experimental paradigm. **b**, Western blot analysis of Drp1 protein expression in the M1 region of Sham, SCI and SCI + rTMS mice (left), and quantification of the relative protein levels of Drp1, normalized to GAPDH (right) (*n* = 6 per group). **c** Representative TEM images showing the ultramicrostructure of GABAergic neurons in layer V of the M1 in Sham, SCI and SCI + rTMS groups (scale bars, 500 nm). The orange arrows and dotted lines indicate mitochondria, the blue arrows indicate synaptic structures (Gray II-type), and the golden triangles indicate the synaptic vesicles. **d**–**g** Quantitative analysis of mitochondrial parameters in GABAergic neurons in mitochondrial area (**d**), mitochondrial perimeter (**e**), mitochondrial density (**f**), and mitochondrial circularity (**g**) in Sham (*n* = 18), SCI (*n* = 15) and SCI + rTMS (*n* = 24) groups. rTMS refers to HF-rTMS. *P < 0.05, **P < 0.01, ***P < 0.001 and ****P < 0.0001.

We next examined whether the therapeutic effects of HF-rTMS were associated with a rescue of this mitochondrial pathology. Remarkably, 5 weeks of HF-rTMS treatment reversed the SCI-induced downregulation of Drp1 protein in the M1 cortex (Fig. 5a, b). The TEM analysis provided structural corroboration. In GABAergic neurons, HF-rTMS effectively normalized mitochondrial morphology: it significantly reduced the enlarged area and perimeter, increased the depleted mitochondrial density back toward the sham level and reduced the elevated circularity (Fig. 5c–g). This pattern is consistent with restored Drp1-mediated fission activity. In glutamatergic neurons, HF-rTMS only partially attenuated the increases in area and perimeter and had no significant effect on density or circularity (Supplementary Fig. 6), reinforcing the cell-type-specific nature of the intervention.

**Fig. 6:**
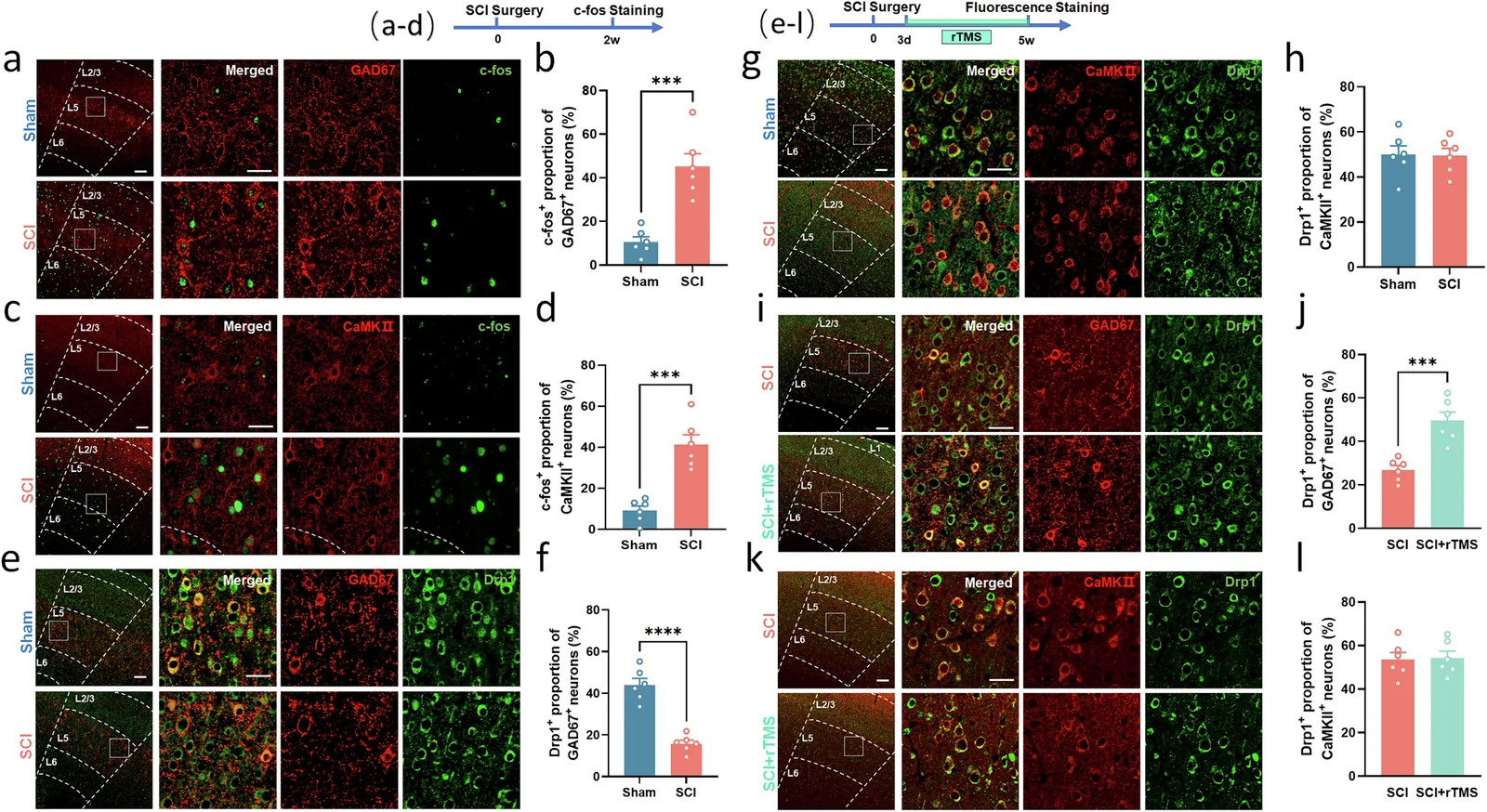
rTMS mediates its effects via GABAergic neurons in layer V of M1. **a**–**d** Representative confocal images of CaMKII-c-Fos positive neurons (**a**) and GAD67-c-Fos positive neurons (**c**) in the layer V of M1 at 2 weeks post SCI (scale bars, 100 μm, left; scale bars, 20 μm, right); quantitative analysis of the proportion of c-Fos^+^ cells among CaMKII neurons (**b**) and GAD67 neurons (**d**) (*n* = 6 slices). **e**–**h** Representative confocal images of CaMKII-Drp1 positive neurons (**e**) and GAD67-Drp1 positive neurons (**g**) in the layer V of M1 at 5 weeks post SCI (scale bars, 100 μm, left; scale bars, 20 μm, right); quantitative analysis of the proportion of Drp1^+^ cells among CaMKII neurons (**f**) and GAD67 neurons (**h**) (*n* = 6 slices). **i**–**l** Representative confocal images of CaMKII-Drp1 positive neurons (**i**) and GAD67-Drp1 positive neurons (**k**) for SCI and SCI + rTMS groups in the layer V of M1 at 5 weeks post SCI (scale bars, 100 μm, left; scale bars, 20 μm, right); quantitative analysis of the proportion of Drp1^+^ cells among CaMKII neurons (**j**) and GAD67 neurons (**l**) (*n* = 6 slices). rTMS refers to HF-rTMS. *P < 0.05, **P < 0.01.

To link Drp1 expression directly to neuronal subtypes, we performed confocal imaging on the M1 region of SCI mice. We initially confirmed neuronal activation in M1 layer V post SCI by observing a significant increase in c-Fos^+^ nuclei, which colocalized with both GABAergic (GAD67^+^) and glutamatergic (CaMKII^+^) neuronal markers (Fig. 6a–d). Next, we analyzed Drp1 expression within these populations. The analysis revealed a significant reduction in the proportion of Drp1^+^ neurons among GAD67^+^ neurons following SCI (Fig. 6e, f); however, no substantial change was observed in CaMKII^+^ neurons (Fig. 6g, h), mirroring the western blot and TEM data. Critically, HF-rTMS treatment specifically restored Drp1 expression within the GAD67^+^ neuronal population (Fig. 6i, j), without affecting its expression in CaMKII^+^ neurons (Fig. 6k, l). This precise, cell-type-restricted upregulation of Drp1 by HF-rTMS aligns perfectly with the selective rescue of mitochondrial morphology in GABAergic neurons and positions Drp1 as a key molecular effector of HF-rTMS.

### Drp1 OE in GABAergic neurons recapitulates HF-rTMS benefits

**Fig. 7:**
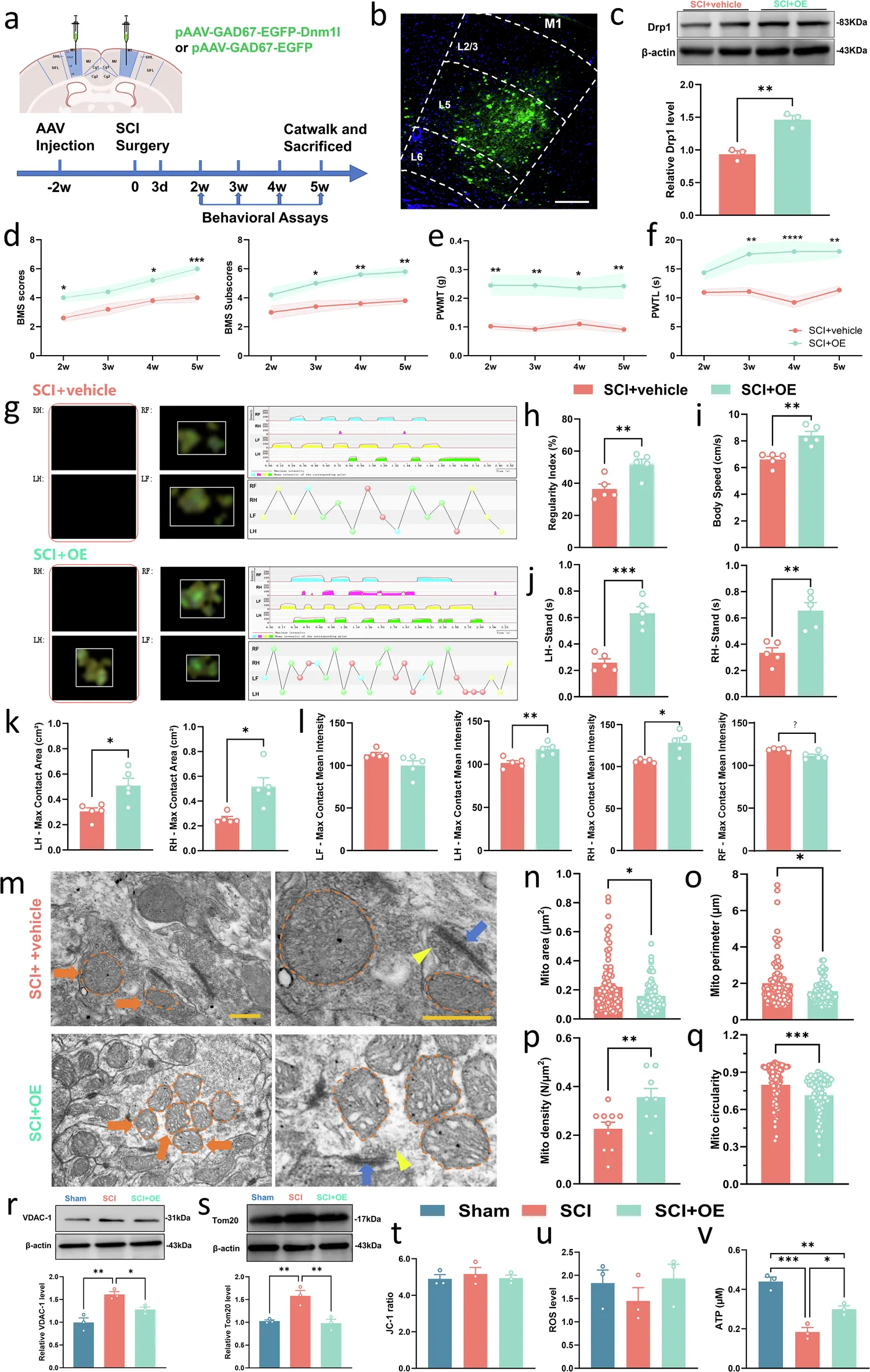
Drp1 OE in GABAergic neurons mimics HF-rTMS benefits on behavioral and mitochondrial recovery after SCI. **a** Schematic timeline of the experimental procedure. **b** Schematic representation of an IF image showing the expression of the EGFP marker in the layer V of M1 region (scale bars, 200 μm). **c**, Western blot analysis of Drp1 protein expression in the M1 region of SCI mice treated with a vehicle (SCI + vehicle) or OE (SCI + OE) (top). Quantitative analysis of the relative protein levels of Drp1, normalized to β-actin (bottom) (*n* = 6 per group). **d** BMS scores in SCI + vehicle and SCI + OE mice (*n* = 6 per group). **e**, **f** Assessment of mechanical allodynia (**e**) and thermal hyperalgesia (**f**) in SCI + vehicle and SCI + OE mice (*n* = 6 per group). **g**–**l**, Quantification of CatWalk gait analysis in SCI + vehicle and SCI + OE mice at 5 weeks post SCI, as measured by the regularity index (**h**), body speed (**i**), the stand time (**j**), maximum paw contact area (**k**) of hind paw prints, and the mean intensity of paw prints (**l**) (*n* = 6 per group). **m**–**q**, TEM images of mitochondria in the M1 region of SCI + vehicle and SCI + OE mice (scale bars, 500 nm) quantified by the area (**n**) perimeter (**o**), density (**p**) and circularity (**q**) of mitochondria in SCI + vehicle (*n* = 83) and SCI + OE (*n* = 67) groups. The orange arrows and dotted lines identify mitochondria, the blue arrows identify the synaptic structure of Gray II-type, and the golden triangles identify synaptic vesicles. **r**, **s** Western blot analysis and quantification of VDAC-1 (**r**) and Tom20 (**s**) protein expression in the M1 region, normalized to β-actin (*n* = 6 per group). **t**–**v** Quantification of mitochondrial function (*n* = 6 per group), including JC-1 ratio (**t**), ROS level (**u**) and ATP (**v**). *P < 0.05, **P < 0.01 and ***P < 0.001.

To establish a direct causal role for Drp1 in GABAergic neurons, we used a gene therapy approach. We injected an AAV vector driving Drp1 overexpression (OE) specifically in GAD67-expressing neurons (pAAV-GAD67-EGFP-*Dnm1l*) into layer V of the M1 cortex of mice before SCI (Fig. 7a). Successful OE was confirmed by IF and western blot (Fig. 7b, c).

Drp1 OE in GABAergic neurons profoundly influenced recovery. SCI mice with Drp1 OE exhibited significantly enhanced motor recovery, with higher BMS scores from week 2 onward compared with SCI mice receiving a control virus (Fig. 7d). Pain behavior was also markedly improved, with alleviation of both mechanical allodynia and thermal hyperalgesia (Fig. 7e, f). CatWalk analysis at 5 weeks confirmed comprehensive improvement: increased regularity index and body speed, alongside partially restored standing duration, contact area and contact intensity of hind paws (Fig. 7g–l).

At the subcellular level, Drp1 OE in GABAergic neurons prevented the pathological mitochondrial enlargement seen after SCI, resulting in smaller mitochondria, higher density and lower circularity (Fig. 7m–q), directly mirroring the effects of HF-rTMS. Furthermore, it normalized the elevated levels of mitophagy markers VDAC-1 and Tom20 (Fig. 7r, s) and, most importantly, significantly increased ATP content toward sham levels in M1 tissue (Fig. 7v), without altering membrane potential or ROS levels (Fig. 7t, u). Thus, targeted Drp1 upregulation in GABAergic neurons is sufficient to restore mitochondrial health, bioenergetics and functional recovery.

### Cell-type-specific validation of Drp1-mediated mitochondrial dysfunction in GABAergic neurons

To directly assess mitochondrial dysfunction within GABAergic neurons, we performed fluorescence co-staining for mitochondrial superoxide (MitoSOX Red) and ATP synthase subunit (ATP5A) together with GAD67 as a GABAergic marker in M1 layer V. In SCI mice, GAD67^+^ neurons exhibited a marked increase in MitoSOX fluorescence intensity and decreased ATP5A immunoreactivity compared with sham controls, indicating elevated mitochondrial oxidative stress and compromised oxidative phosphorylation capacity. Both HF-rTMS treatment and Drp1 OE in GABAergic neurons significantly reversed these abnormalities (Supplementary Fig. 7). Notably, whereas whole-tissue ROS levels did not differ significantly between SCI and Sham groups (Figs. 4l and 7u), the cell-type-specific fluorescence approach revealed robust mitochondrial oxidative stress selectively within GABAergic neurons (Supplementary Fig. 7b). By contrast, ATP depletion was consistently detected in both whole-tissue homogenates (Figs. 4m and 7v) and GABAergic neuron-specific ATP5A staining (Supplementary Fig. 7d). This consistent reduction validates that bioenergetic insufficiency arises specifically within this neuronal subtype.

### Drp1 knockdown in GABAergic neurons exacerbates deficits and ablates HF-rTMS efficacy

Conversely, we used an AAV encoding a shRNA to knock down Drp1 specifically in GAD67^+^ neurons (Supplementary Fig. 8a, b). This knockdown exacerbated the SCI phenotype, leading to worse motor recovery (lower BMS scores), more severe allodynia and hyperalgesia and a lack of improvement in CatWalk gait parameters (Supplementary Fig. 8c–k). Ultrastructurally, it intensified mitochondrial enlargement and reduced density in GABAergic neurons (Supplementary Fig. 8l–p) but could not rescue the ATP reduction, alongside disrupted mitochondrial membrane homeostasis and oxidative stress accumulation (Supplementary Fig. 8q–u).

Most decisively, Drp1 knockdown completely abolished the therapeutic effects of HF-rTMS. In mice with Drp1 knocked down in GABAergic neurons, subsequent HF-rTMS treatment failed to elevate Drp1 protein levels (Supplementary Fig. 9a, b) and conferred no significant benefit on motor function (BMS), pain behavior (PWMT and PWTL) or gait analysis parameters compared with untreated SCI mice (Supplementary Fig. 9c–k). This demonstrates that the presence of Drp1, specifically within layer V M1 GABAergic neurons, is absolutely required for HF-rTMS to exert its therapeutic action.

Considering the crucial function of the primary somatosensory cortex (S1) in pain perception^36^, we scrutinized the alterations in the S1 region post SCI, particularly the S1HL subregion responsible for the hind paw. IF results indicated a significant upsurge in c-Fos activation in the S1HL region of SCI mice (Supplementary Fig. 10a, b). These results suggest that the neurons in the S1HL are involved in SCI-NP. By comparing the expression in the S1HL region before and after HF-rTMS, we observed that HF-rTMS failed to modulate Drp1 expression in either CaMKII^+^ or GAD67^+^ neurons (Supplementary Fig. 10c–g), which may underlie the suboptimal analgesic outcomes of rTMS when targeting the S1 region in clinical studies^37^. In conclusion, our findings affirm that the therapeutic efficacy of HF-rTMS is contingent upon Drp1 expression within layer V GABAergic neurons of the M1 region.

### Pharmacological inhibition of Drp1 blocks the therapeutic action of HF-rTMS

**Fig. 8:**
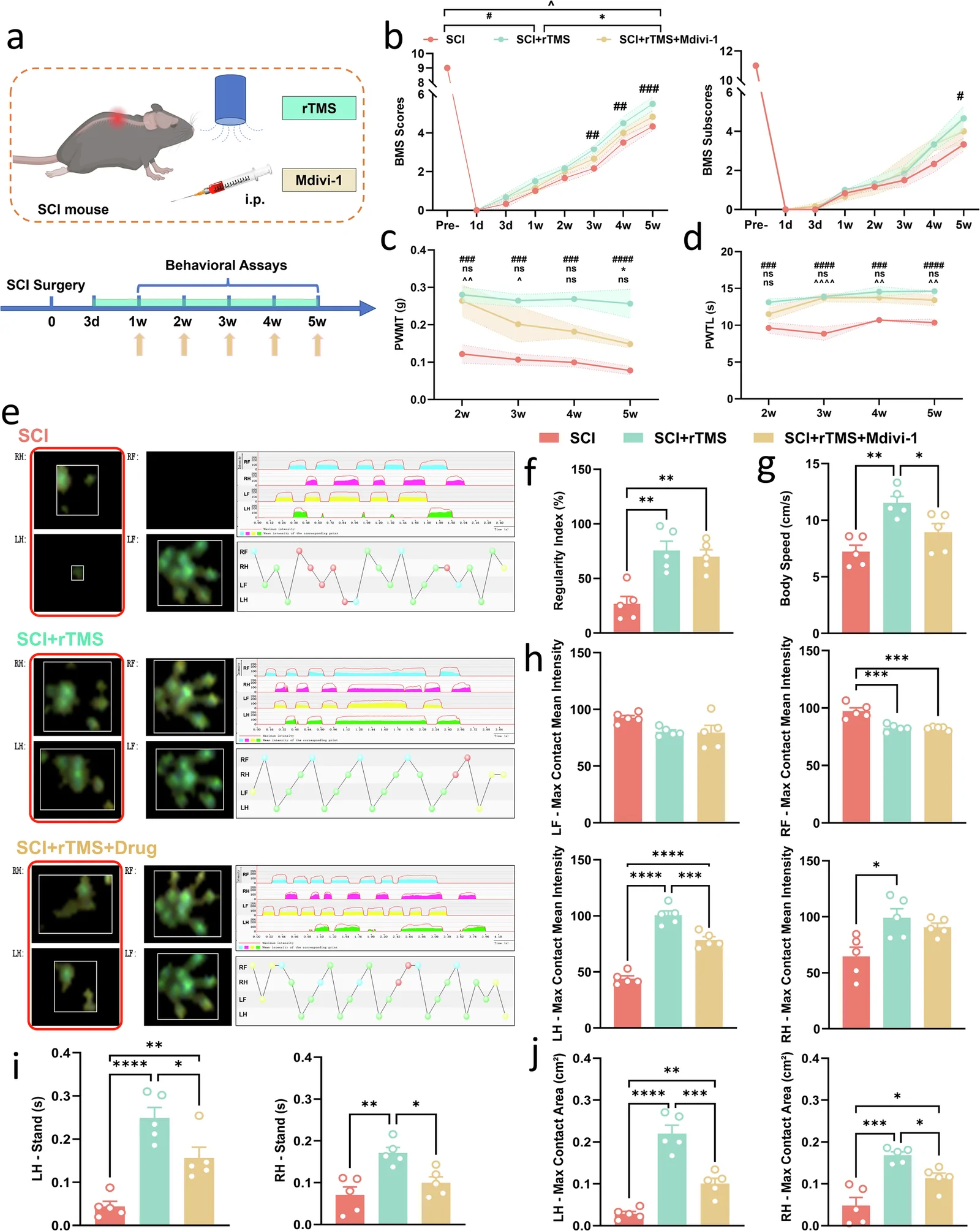
HF-rTMS exerts therapeutic effects through Drp1. **a** Schematic representation of the experimental paradigm. **b** BMS scores evaluating locomotor function in SCI, SCI + rTMS and SCI + rTMS + Mdivi-1 mice. **c**, **d** Assessment of mechanical allodynia (**c**) and thermal hyperalgesia (**d**) in SCI, SCI + rTMS and SCI + rTMS + Mdivi-1 mice. **e** The typical mouse paw print in CatWalk, where the red box is the hind paw print. **f**–**j** Quantification of CatWalk gait analysis in SCI and SCI + rTMS mice with and without Mdivi-1 at 5 weeks post SCI, as measured by the regularity index (**f**), body speed (**g**), the mean intensity of paw prints (**h**), the stand time (**i**), and maximum paw contact area (**j**) of hind paw prints. Line graphs (**b**–**d**) use # (SCI versus SCI + rTMS), ^ (SCI versus SCI + rTMS + Mdivi-1), * (SCI + rTMS versus SCI + rTMS + Mdivi-1). Graded symbols correspond to *P* < 0.05, 0.01, 0.001, 0.0001. ns, not significant. *n* = 6 mice per group in all. rTMS refers to HF-rTMS. *P < 0.05, **P < 0.01, ***P < 0.001 and ****P < 0.0001.

Having established the cell-type-specific nature of Drp1-mediated mitochondrial dysfunction in GABAergic neurons, we next explored the translational implications of this mechanism. We administered Mdivi-1, a pharmacological inhibitor of Drp1^38^, to SCI mice receiving HF-rTMS (Fig. 8a). Mdivi-1 treatment effectively nullified the benefits of HF-rTMS. It prevented the improvement in motor function (Fig. 8b) and significantly blunted the analgesic effect on mechanical allodynia (Fig. 8c, d). The CatWalk analysis confirmed the loss of therapeutic gain across multiple parameters (Fig. 8e–j). This experiment confirms that Drp1 is an essential molecular mediator underlying the therapeutic effects of HF-rTMS and reveals that combined use of Drp1-targeted drugs may interfere with the clinical efficacy of rTMS intervention.

## Discussion

This study elucidates a novel and coherent mechanism through which HF-rTMS alleviates the comorbid motor and NP deficits following SCI, centered on the restoration of mitochondrial dynamics in a specific cortical microcircuit. We first provided clinical evidence from a retrospective cohort demonstrating the dual symptomatic efficacy of HF-rTMS. Using a translational mouse model, we then systematically dissected the underlying pathway. Our snRNA-Seq analysis revealed that SCI and therapeutic HF-rTMS exert opposing transcriptional influences converging on mitochondrial energy metabolism pathways in the M1 cortex, with notable enrichment in GABAergic neurons. Pursuing this lead, we discovered that SCI induces a persistent suppression of the mitochondrial fission protein Drp1 specifically within layer V M1 GABAergic interneurons. This Drp1 deficiency drives a pathological shift in mitochondrial dynamics, leading to enlarged, sparse and dysfunctional organelles, culminating in a bioenergetic deficit (reduced ATP). HF-rTMS precisely reverses this cascade by restoring Drp1 expression in these neurons, thereby normalizing mitochondrial morphology, density and energy production. Using gain- and loss-of-function experiments, we established a direct causal relationship: Drp1 OE in GABAergic neurons mimicked HF-rTMS benefits, while its knockdown ablated them. Finally, pharmacological inhibition of Drp1 confirmed that its activity is essential for HF-rTMS efficacy. Therefore, we propose that HF-rTMS ameliorates SCI comorbidity by selectively upregulating Drp1 in layer V M1 GABAergic neurons, rescuing mitochondrial fission and restoring cortical bioenergetic homeostasis.

The present findings advance our understanding of SCI pathophysiology and rTMS mechanisms in several important respects. First, most prior studies linking SCI to Drp1 dysregulation and mitochondrial dysfunction have focused on spinal cord neurons at the primary lesion site or relied on bulk cortical tissue homogenates that lack cell-type resolution^39,40^. Few have pinpointed Drp1-mediated mitochondrial pathology in defined neuronal subtypes within supraspinal regions following thoracic SCI. Using snRNA-Seq and cell-type-specific analyses, our study identifies layer V GABAergic interneurons in M1 as the predominant vulnerable population, thereby extending the recognized scope of post-SCI mitochondrial pathology from the spinal lesion to specific cortical microcircuits. Second, although preclinical studies have demonstrated the therapeutic potential of rTMS for SCI-related motor deficits and NP, the proposed mechanisms have largely centered on suppressing neuroinflammation, attenuating glial scarring and promoting neuronal survival at the injury site^10,41^. These interpretations rarely identified a definitive molecular target or a cell-type-specific pathway underlying rTMS-mediated cortical recovery, leaving unclear how rTMS improves remote circuit function. Third, our study establishes a mechanistic cascade in which therapeutic rTMS restores Drp1 expression to correct pathological mitochondrial fragmentation, thereby rescuing oxidative phosphorylation and ATP production selectively within M1 layer V GABAergic neurons. This cell-type-specific Drp1-mitochondrial axis defines a novel therapeutic target distinct from previously reported inflammation- or glia-oriented mechanisms. Importantly, our gain- and loss-of-function experiments demonstrate that Drp1 is both necessary and sufficient for rTMS efficacy, providing a level of causal evidence in neuromodulation research. This framework may have broader implications for understanding how noninvasive brain stimulation achieves durable effects across neurological and psychiatric conditions.

The frequent co-occurrence of motor impairment and NP after SCI presents a major therapeutic challenge, as pain can directly inhibit engagement in rehabilitative motor training^42,43^. Our findings position HF-rTMS as a promising noninvasive modality capable of breaking this vicious cycle. The clinical data align with and extend previous literature showing separate benefits of M1-targeted rTMS for motor recovery or NP^44–46^. The novelty here lies in demonstrating concurrent improvement, addressing the comorbidity directly. The convergence of clinical and robust preclinical data strengthens the translational validity of our mechanistic findings.

The cell-type specificity uncovered is critical. Layer V of M1 harbors both corticospinal projection (glutamatergic) neurons and a diverse array of GABAergic interneurons that shape cortical output^47^. Our data point to GABAergic interneurons as the primary cellular target for the restorative effect of HF-rTMS on mitochondrial dynamics. This specificity may be rooted in their unique metabolic demands and role in maintaining cortical E/I balance^6,48^. SCI-induced NP is associated with reduced GABAergic inhibition (disinhibition) in sensory and motor cortices. The observed Drp1 downregulation and mitochondrial dysfunction in these neurons could impair their energy-intensive firing and synaptic signaling, contributing to disinhibition and maladaptive plasticity^30,49,50^. By rescuing mitochondrial function specifically in this population, HF-rTMS may restore their inhibitory capacity, thereby re-normalizing cortical E/I balance^6,51,52^. This interpretation is supported by studies showing that rTMS can enhance GABAergic transmission^53,54^. Consistently, our cell-type-specific fluorescent staining further validates this model. In SCI mice, we observed selective mitochondrial dysfunction of GABAergic neurons in M1 layer V, including elevated MitoSOX and diminished ATP5A signals. Either HF-rTMS or Drp1 OE could reverse these defects. Notably, whereas whole-tissue ROS assays did not detect significant differences between SCI and Sham groups, the cell-type-specific approach revealed robust mitochondrial oxidative stress within GABAergic neurons. This apparent discrepancy is not contradictory but rather reflects the inherent limitation of tissue homogenate-based assays, which average signals across the diverse cell populations of the M1 cortex. As GABAergic neurons constitute only approximately 15–20% of M1 cortical cells, their cell-type-specific pathology can be masked by signal dilution from other cell populations with distinct metabolic profiles. By contrast, ATP depletion was consistently detected in both whole-tissue homogenates and GABAergic neuron-specific ATP5A staining, confirming that the bioenergetic deficit in this neuronal population is functionally consequential and of sufficient magnitude to overcome such dilution. These cell-type-resolved findings provide direct evidence that SCI induces mitochondrial oxidative stress and bioenergetic failure in M1 layer V GABAergic neurons, and underscore the necessity of cell-type-specific approaches when investigating mitochondrial pathology in heterogeneous brain tissue. In contrast to GABAergic neurons, the more limited mitochondrial response in glutamatergic neurons suggests their role may be more related to structural plasticity or network integration, with different metabolic priorities.

To our knowledge, this study provides the first cell-type-specific evidence linking the therapeutic effects of rTMS to the precise regulation of mitochondrial fission dynamics. Mitochondria are not merely passive energy suppliers but dynamic signaling hubs influencing synaptic plasticity, calcium buffering and cell survival^55^. The snRNA-Seq signature highlighting oxidative phosphorylation, coupled with the functional rescue of ATP levels by both HF-rTMS and Drp1 OE, firmly establishes bioenergetic recovery as a central mechanism. The pathological mitochondrial enlargement (due to fission failure) and the restorative effect of HF-rTMS/Drp1 OE provide a clear structural correlate. The increase in mitochondrial mass markers (VDAC-1 and Tom20) post SCI probably reflects an accumulation of dysfunctional organelles secondary to impaired mitochondrial turnover rather than successful mitophagic clearance. HF-rTMS, by restoring fission, may facilitate the generation of healthy mitochondria and the efficient clearance of damaged ones, ultimately re-establishing a healthy mitochondrial network.

The necessity and sufficiency of Drp1 in GABAergic neurons were rigorously demonstrated. The AAV-mediated knockdown experiment is particularly compelling, as it demonstrates that even in the presence of HF-rTMS, functional recovery is blocked if Drp1 cannot be upregulated in this specific cellular compartment. This identifies Drp1 as a nonredundant, bidirectional mediator of rTMS responsiveness in SCI recovery. The Mdivi-1 experiment further translates this finding, revealing a potential clinical vulnerability: concomitant use of drugs that inhibit mitochondrial fission (such as olanzapine, amitriptyline, escitalopram and haloperidol)^56,57^ could inadvertently negate the benefits of rTMS therapy. This underscores the importance of considering a patient’s pharmacogenomic profile in neuromodulation treatment planning, while clinical validation of their interaction with rTMS in patients with SCI remains to be established.

An intriguing observation was the lack of Drp1 modulation by HF-rTMS in S1, a region also involved in pain processing^58^. This region-specific molecular response may explain why rTMS protocols targeting M1 often show superior or more consistent analgesic effects compared with those targeting S1^9,37,59^. The preferential engagement of M1 over S1 in SCI-induced pain control can be understood through their fundamentally distinct anatomical and functional positions within the central pain matrix. Seminal work by Gan et al. demonstrated that M1-derived analgesia operates through a layer-specific dual-pathway architecture: M1 layer V neurons project to the zona incerta (ZI) and periaqueductal gray to suppress sensory hypersensitivity, whereas layer VI neurons connect with the mediodorsal thalamus and nucleus accumbens (NAc) to specifically alleviate the affective-motivational dimension of pain^6^. This hierarchical organization positions M1 as uniquely capable of concurrently addressing both sensory and emotional components of chronic pain—a dual benefit not equally afforded by S1-targeted interventions. Beyond their independent roles, M1 and S1 are functionally coupled within a cortico-cortical network that becomes pathologically remodeled after SCI. Clinical neuroimaging studies have established that the degree of S1 cortical reorganization, including gray matter volume shrinkage, significantly correlates with ongoing NP intensity^60^. However, this S1 pathology does not act in isolation to generate pain. A pivotal study by Wang et al. (Neuron 2025^21^) delineated a canonical sensory–motor–sensory feedback circuit, in which nociceptive signals originating from S1 converge onto M1 to trigger powerful descending antinociceptive output. In this model, the reactivation of M1 can rectify the S1–M1 microcircuit imbalance and reinstate descending pain control, as S1-derived nociceptive signals converge onto M1, and M1 output in turn gates ascending nociceptive transmission via its brainstem and spinal projections^21^. Furthermore, M1-mediated analgesia may engage S1 as a downstream effector: recent evidence demonstrates that M1 stimulation activates somatostatin-expressing inhibitory interneurons within S1, which suppress layer V pyramidal neuron hyperactivity, thereby contributing to prolonged analgesia^61^. Within this hierarchical framework, M1 occupies a rate-limiting node: it is both the recipient of pathological S1-derived nociceptive drive and the sole cortical origin of the robust, layer-defined descending pathways to the periaqueductal gray, zona incerta and other nodes of the endogenous pain modulatory system^6,21^. Although these studies were performed in non-SCI pain models, the conserved circuit logic provides a mechanistic rationale for our observations. Our finding that HF-rTMS restores Drp1 expression and mitochondrial bioenergetics selectively in M1 GABAergic neurons, but not in S1, is consistent with this hierarchical framework. By rescuing the metabolic capacity of M1 inhibitory microcircuitry, rTMS may strengthen the cortical output required for effective descending analgesia, without necessitating direct molecular modulation in S1. M1’s unique connectivity to descending inhibitory pain pathways and subcortical regions involved in the affective–cognitive aspects of pain^21,62,63^, combined with its sensitivity to mitochondrial modulation via rTMS in our study, may collectively render it a more effective and mechanistically grounded target for treating the complex, multidimensional pain state following SCI.

Several limitations should be acknowledged. First, while we used a standard rodent coil^21,22^, the spatial precision of TMS in a small mouse brain is imperfect, and effects on adjacent regions cannot be entirely excluded. However, our subsequent highly precise AAV manipulations confirm that the M1 layer V GABAergic circuit is the critical site of action. Second, the Mdivi-1 experiment further demonstrates that concomitant medications affecting mitochondrial dynamics could modulate rTMS responsiveness. The retrospective review of our cohort’s medication histories revealed comparable pregabalin use between groups, and exclusion of the single venlafaxine-treated patient did not alter the analgesic outcome. Nevertheless, our medication data are inherently limited to what was documented in medical records and lack granular information on dosage, treatment duration and adherence, and we cannot exclude effects of unrecorded medications or supplements with mitochondrial effects. To overcome these constraints, we have initiated a multicenter, double-blind, sham-controlled randomized clinical trial (RCT). This ongoing prospective RCT (ChiCTR2400094593) will provide the high-level evidence required to inform clinical guidelines and therapeutic practice. Finally, the detailed molecular pathways connecting rTMS-induced neuronal activity to the transcriptional upregulation of Drp1 require further investigation, potentially involving activity-dependent transcription factors or epigenetic modifiers, as well as the biological significance of glial cell-related pathways.

This work provides a multilevel understanding of HF-rTMS action in SCI, bridging from human clinical effects to a specific cellular and organellar mechanism. We identify the regulation of Drp1-dependent mitochondrial fission in layer V M1 GABAergic neurons as a critical pathway through which HF-rTMS restores bioenergetic capacity and cortical circuit function, leading to concurrent motor and pain recovery. These findings not only advance the mechanistic understanding of neuromodulation but also highlight mitochondrial dynamics and GABAergic interneuron health as promising targets for developing new therapeutic strategies for SCI and potentially other disorders involving central nervous system injury and circuit dysfunction.

## Supplementary information

Supplementary Information

Supporting data values

Unedited blot and gel images

RCT: retrospective study

RCT: prospective study

## Data Availability

The snRNA-Seq data used in this study have been deposited in the NCBI’s Sequence Read Archive (SRA) (SRA study accession code, PRJNA1299693; https://dataview.ncbi.nlm.nih.gov/object/PRJNA1299693SRP323752). Other data associated with this study are present in this article or the supplemental materials. Additional data that support the findings of this study are available from the corresponding author upon reasonable request.
