# Supplementary material for: Drp1 in M1 layer V GABAergic neurons orchestrates rTMS-mediated motor restoration and analgesia after spinal cord injury
Source: Exp Mol Med. 2026 Oct 5;58(10):3440–56. doi: 10.1038/s12276-026-01857-2 (PMC13737150; doi:10.1038/s12276-026-01857-2)
Supplement: Supplementary file 1 [file 12276_2026_1857_MOESM1_ESM.pdf]

Supplementary Information Files

Drp1 in M1 Layer V GABAergic Neurons Orchestrates rTMS-mediated Motor Restoration and Analgesia after spinal cord injury

Supplementary methods

Hematoxylin and eosin (H&E) staining

H&E staining is the most basic and widely used technique in the teaching and research of histology, embryology and pathology. The chromatin in the nucleus and the nucleic acid in the cytoplasm are purplish-blue using hematoxylin dye; and eosin dye is used to make components in the cytoplasm and extracellular matrix red. Briefly, mouse T9 spinal cord was removed and immersed in 4% paraformaldehyde solution for 24 h. The spinal cord was fixed in 4% paraformaldehyde solution for 24 h and then placed in a dehydration box for dehydration. The embedding cassettes were placed in paraffin wax and replaced three times with paraffin waxes of different melting points for 1 h. The soaked tissues were embedded using an embeddingapparatus. Wax blocks with different melting points were melted in incubators at different temperatures and poured into the embedding box, and the tissues were embedded in unconsolidated wax and labeled. The wax blocks were cooled on a cryogenic platform at -20°C. After cooling and solidifying, the wax blocks were removed from the encapsulation frame and trimmed. The wax blocks were sectioned using a microtome to a thickness of 4 μm. The slices were floated at 35°C. The slices were removed, covered with a coverslip and placed in an oven at 60°C for baking. Sections were dewaxed with dewaxing clear solution and hydrated through a graded series of alcohol with dehydration time of 4 min. Sections were placed in staining solution for 5 min, after which the sections were rinsed with ultrapure water, differentiated with acid alcohol and rinsed with tap water, followed by blueing for 20-40 s and dehydration with 80% ethanol. Finally, the sections were stained with eosin, and the staining time was controlled at 30 s to 3 min, followed by differentiation with dilute alcohol. The stained sections were dehydrated within different volume fractions of alcohol, followed by cleared in xylene, and finally sealed with neutral balsam.

Nissl staining

Three mice were taken from each group and injected intraperitoneally with 5 % sodium isopentobarbital at 5 mg/kg body weight. After deep anesthesia, the needle was inserted through the left ventricle of the mice, and the left ventricle of the mice was rapidly perfused with 30 ml of 0.01 mol/L phosphate buffered saline (PBS), and then replaced with 30 ml of phosphate buffer (PB) containing 4 % paraformaldehyde, and the spinal cord of the T9 segment was taken and fixed in 4 % paraformaldehyde solution for 4-6 h. After dehydration in overnight, the tissues were removed

and coronal frozen sections were made, with a thickness of 30 μm. The slices were dyed with toluidine blue, kept at a constant temperature of 50-60 ℃ for 25-50 min, followed by rapid differentiation and dehydration with gradient alcohol, and then cleared in xylene and sealed with neutral resin.

Immunofluorescence (IF)

The experimental animals were perfused transcardially with 4% paraformaldehyde. Then the fixed brains were sectioned. The coronal slices containing M1 were incubated in the following primary antibodies: rabbit anti-Drp1 (Abcam, 1:250), mouse anti-GAD67 (Sigma, 1:1000), mouse anti-CaMKII (Abcam, 1:500), rabbit anti-ATP5A (Immunoway, 1:500) and rabbit anti-c-Fos (CST, 1:1000) in 0.01 M PBS containing 1% bovine serum albumin and 0.3% Triton X-100 for 24 h at 4 °C. After washing, the sections were labeled with Alexa Fluor 594 (Invitrogen, 1:2000) or Alexa Fluor 488 (Invitrogen, 1:2000) for 2 h at room temperature. FV1000 (Olympus) was used for image acquisition. The numbers of positive cells were counted in 6 slices (30 μm thickness) of the M1 obtained from 6 mice per group, and the results were averaged by two investigators blinded to group allocation.

Evaluation of mitochondrial superoxide

Fresh frozen brain sections were rinsed with prewarmed sterile PBS to remove residual embedding medium. Mitochondrial superoxide levels were evaluated using MitoSOX™ Red (Cat#T75342; TargetMol). Working solution containing 2.5 μM MitoSOX™ Red was prepared in accordance with the manufacturer's protocols. Brain slices were completely covered with corresponding probe solutions and incubated for 30 min at 37 °C in a light-shielded humidified environment. Upon completion of incubation, sections were rinsed twice with PBS to discard unbound probes. Subsequent immunofluorescent staining targeting GAD67 was performed to label GABAergic neurons localized in layer V of the M1 cortex for cell-type-specific analysis. Confocal laser scanning microscopy was applied to capture fluorescent signals, with excitation/emission wavelengths set at 510/580 nm for MitoSOX™ Red. The mean fluorescence intensity (MFI) of MitoSOX was quantified by outlining the soma of individual $GAD67^+$ neurons across multiple brain slices, obtained from 6 biologically independent mice per group.

Western blot

Mice were decapitated, brains were removed and placed in brain molds that had been pre-chilled on ice. Motor cortex tissues were removed and fully homogenized using radioimmunoprecipitation assay (RIPA) buffer (Solarbio, Beijing, China) containing protease/phosphatase inhibitor cocktail (Sigma, Germany). NanoDrop™ One (Thermo Scientific) was used to measure the protein concentration of RIPA lysates. Protein extracts were separated by electrophoresis on 10% SDS-PAGE at 120 V. The proteins were transferred to a polyvinylidene difluoride (PVDF) membrane (Millipore, USA) at 100 V. Immunoblots were soaked in 5% skimmed milk for 2 h at room temperature and subsequently detected with the following primary antibodies (4˚C, overnight): rabbit-anti-Drp1 (1:250, Cat No. ab184247, Abcam, USA), rabbit-anti-Mfn1(1:1000, Cat

No.13798-1-AP, Proteintech Group, China), rabbit-anti-Mfn2 (1:1000, Cat No.12186-1-AP, Proteintech Group, China), rabbit-anti-Tom20 (1:500, Cat No. 11802-1-AP, Proteintech Group, China), rabbit-anti-VDAC-1 (1:500, Cat No. 55259-1-AP, Proteintech Group, China), mouse-anti-β-actin (1:5000, Cat No. ABL1010, Abbkine, China) and rabbit-anti-β-actin (1:5000, Cat No. ABL1011, Abbkine, China). They were then incubated with the corresponding horseradish peroxidase (HRP)-conjugated secondary antibodies (1:5000, Cat No. SA00001-1/SA00001-2, Proteintech Group, China). The bands were detected with enhanced chemiluminescence (EpiZyme, China) followed by exposure using a luminometer (Bio-Rad, USA). The open-source FIJI (NIH ImageJ) software was used to quantify the intensity of the immunoreactive bands of interest on autoradiograms. Target protein levels were normalized relative to β-actin levels and expressed as a fold change relative to the control group.

Transmission electron microscopy (TEM)

Mice were anesthetized and perfused sequentially with 0.01 M PBS and 4% formaldehyde with 0.1% glutaraldehyde in PBS. Then, the brain tissues were removed on ice and fixed on a vibrating slicer (Leica 1200 s, Wetzlar, Germany), and coronal slices (300 μm thick) containing M1 (fontanel 1.40-0.55 mm, determined by the shape of the lateral ventricles and corpus callosum) were placed into 4% glutaraldehyde solution for tissue fixation. After fixation, each tissue was washed twice with 0.1 M PB. This was followed by 1% osmium tetroxide (TED PELLA, Inc. No. 18451) for staining for 2 h. Then, 50%, 70%, and 90% ethanol and 100% acetone were used respectively for dehydration, followed by acetone: embedding agent (Embed 812, DDSA, NMA, DMP-30) = 1:1 for 2 h at room temperature. Lastly, the tissue was removed from the embedding agent and stored overnight at room temperature. The following day, the tissue was placed into a special electron microscope mold and incubated at 60 °C for 48 h. A 70 nm thick section was prepared using an ultramicrotome. The slices were placed on a copper grid and stained with lead citrate and uranyl acetate for 10 minutes each. Images were recorded using a TEM (JEM 1400, JEOL, Japan). The mitochondrial morphology data were analyzed using lmageJ-based image analysis.

Single nucleus RNA sequencing (snRNA-Seq)

From the M1 region of the fresh frozen mouse cerebral cortex, approximately 10 mg of tissue was cut and weighed on dry ice using sterile disposable scalpels. The cortex tissue was homogenized in ice-cold homogenization buffer (0.25M sucrose, 5 mM CaCl2, 3 mM MgAc2, 10 mM Tris-Hcl pH 8.0, 0.1 mM EDTA, 1x Protease inhibitor, and 1U/μl Ribolock RNase inhibitors) with a glass-on-glass Dounce homogenizer, 10 strokes with the A pestle, followed by 10 strokes of the B pestle.

Homogenates were passed through a 70 μm cell strainer, to collect the nuclear fraction. The nuclear fraction was mixed with an equal volume of 50 % iodixanol and added on top of a 30-33 % iodixanol solution for 20 min at 10,000 × g, at 4 °C. After the removal of the myelin layer from the top of the gradient, the nuclei were collected from the 30-33 % iodixanol interface. The nuclei were resuspended in nuclei wash and resuspension buffer (0.04 % bovine serum albumin, 0.2U/μl Ribolock RNase inhibitors, 500 mM mannitol and 0.1mM PMSF Protease inhibitor in phosphate-buffered saline) and pelleted for 5 min at 500×g, 4°C. The nuclei were passed through a 40 μm cell strainer to remove cell debris and large clumps. Nuclei concentration was manually determined using counterstaining and hemocytometer. Nuclei concentration was adjusted to 700-1200 nuclei/μL, and immediately processed using the 10X Genomics® Single Cell Protocol. This part was assisted by Gene Denovo Biotechnology Co (Guangzhou, China).

Supplementary Figure Legends

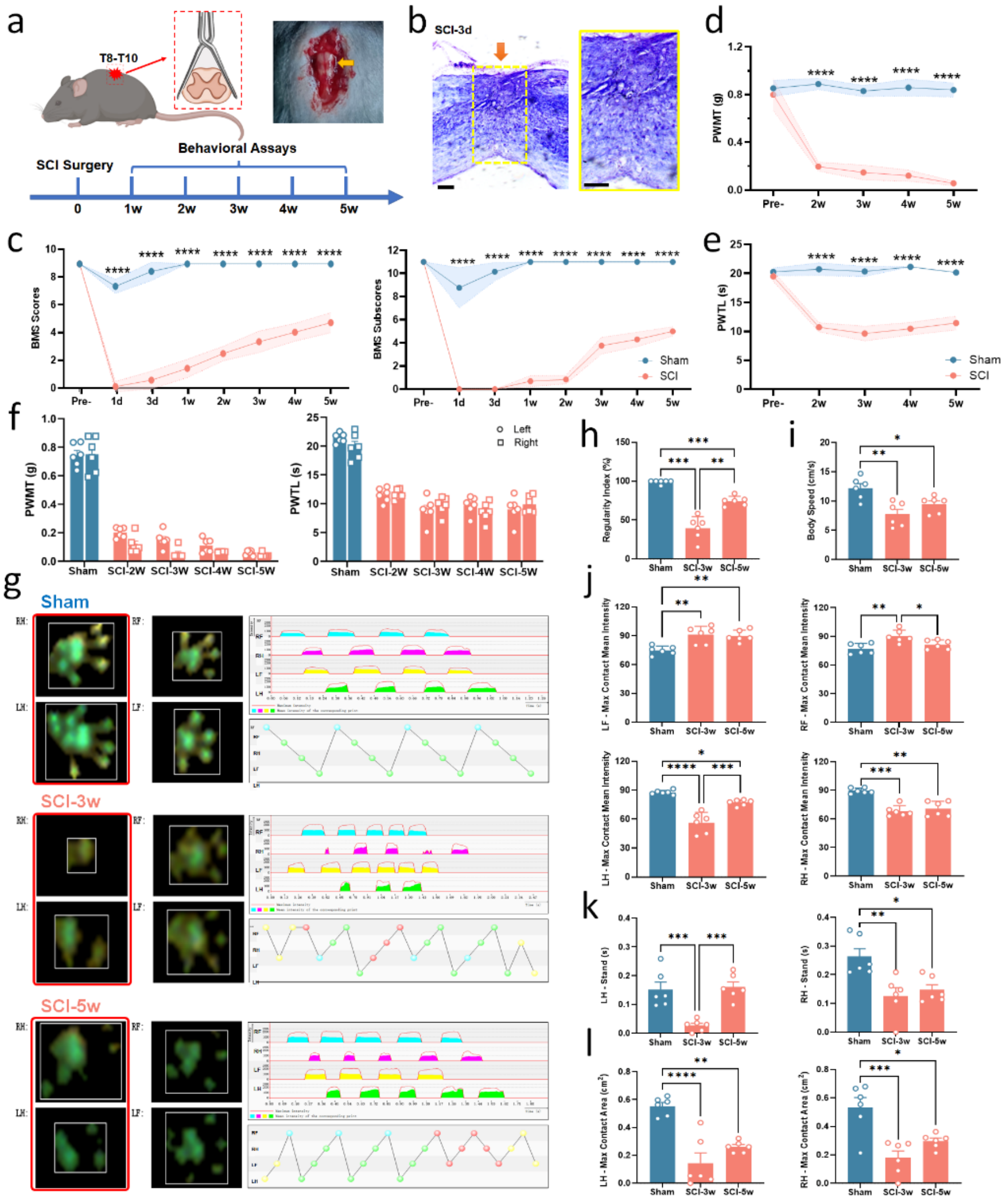

Supplementary Figure 1: SCI induced motor deficit and NP. a, Schematic representation of the spinal cord injury (SCI) model in mice, highlighting the T8-T10 vertebrae as the site of injury. **b,** Nissl staining of thoracic spinal cord sections in SCI-3d mice (Scale bars = 200 μm). **c,** Basso Mouse Scale (BMS) scores assessing locomotor function in SCI mice at different time intervals post-injury. **d,e,** Assessment of mechanical allodynia (**d**) and thermal hyperalgesia (**e**) in Sham and SCI mice at various time points post-surgery. Data are presented as paw withdrawal mechanical threshold (PWMT) and paw withdrawal thermal latency (PWTL), respectively. **f,** Assessment of PWMT and PWTL in bilateral hind paws of SCI mice at various time points post-surgery. **g** depicts a typical mouse paw print in Catwalk, where the red box is the hind paw print. **h-l,** Quantification

of Catwalk gait analysis in Sham and SCI mice at 3 weeks (SCI-3w) and 5 weeks (SCI-5w) post-injury, as measured by the regularity index (**h**), body speed (**i**), the mean intensity of paw prints (**j**), the stand time (**k**) and max paw contact area (**l**) of hind paw prints. $n = 6$ mice per group in all. *$P < 0.05$, **$P < 0.01$, ***$P < 0.001$ and ****$P < 0.0001$.

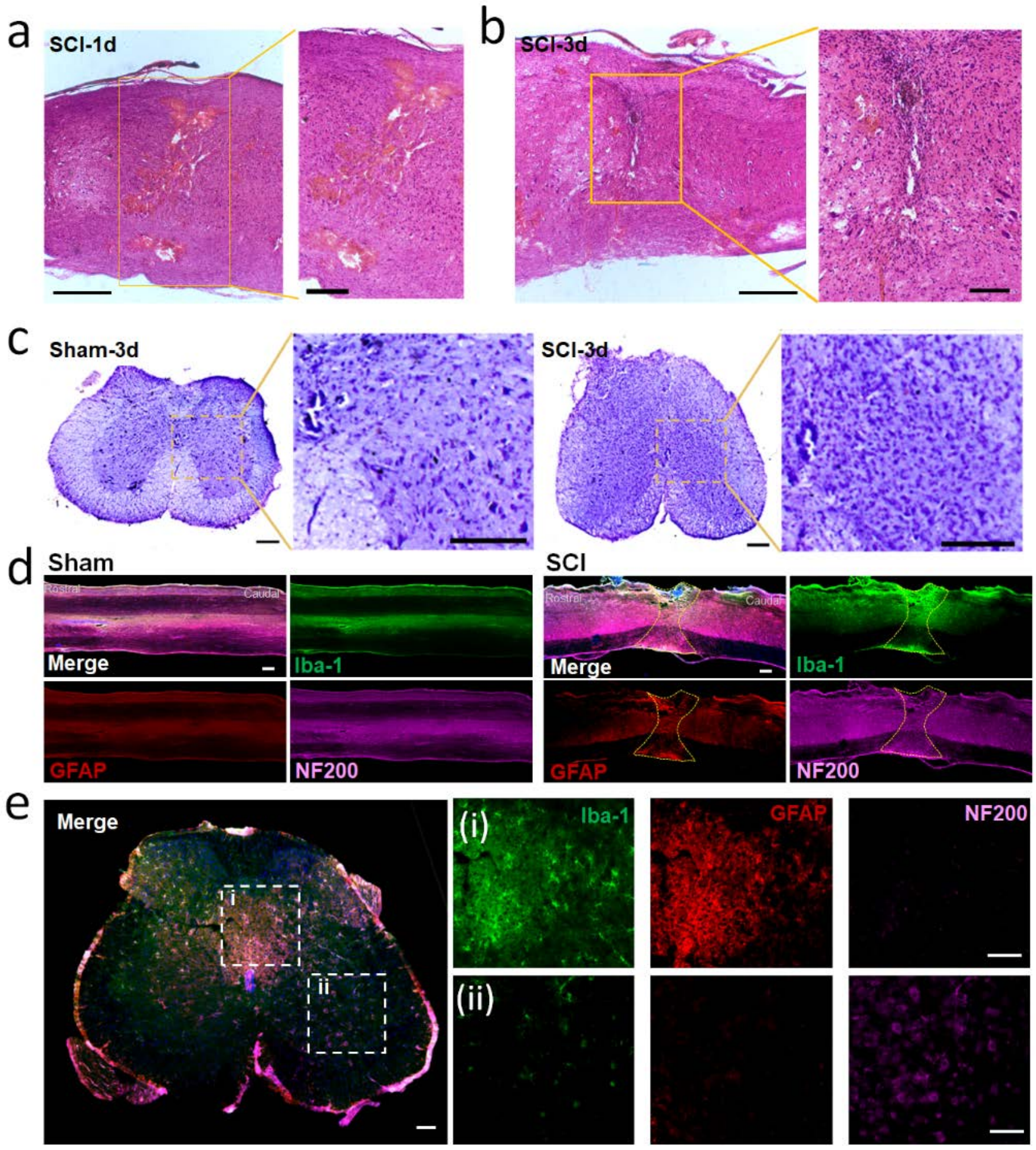

Supplementary Figure 2: Staining, behavioral results and molecular expression of SCI. a,b, Typical images of sagittal surface H&E staining at injury sites of the spinal cord at 1d (**a**) and 3d (**b**) after SCI. **c,** Typical Nissl staining of coronal surface of the spinal cord at the proximal end of the injury site after SCI-3d (Control, left. SCI, right. Scale bars = 200 μm). **d,** Sagittal spinal cord sections stained for NF200 (purple, axons), GFAP (red, astrocytes) and Iba-1 (green, microglia) (Scale bars = 200 μm). Yellow dashed lines outline the SCI lesion core. **e,** Transverse section at the lesion core with merged and separated single-channel immunofluorescence images. (i) Severe axonal loss (NF200) was detected in the lesion center, surrounded by a $GFAP^+$ glial scar,

accompanied by abundant infiltrating Iba-1+ microglia; (ii) The NF200 axonal staining revealed that SCI caused significant but incomplete axonal loss (Scale bars = 100 μm).

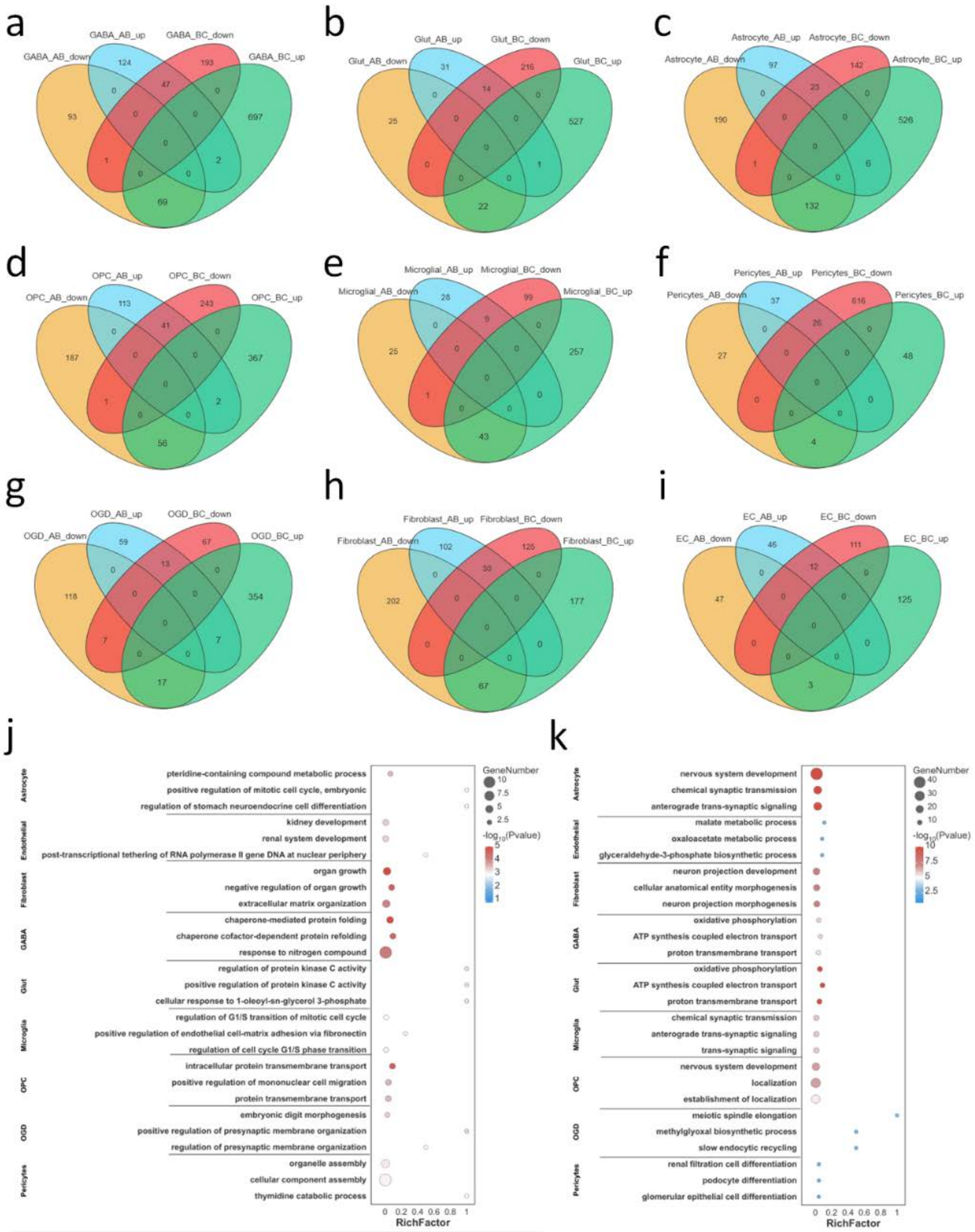

Supplementary Figure 3: Differential gene statistics and GO biological process enrichment analysis across multiple cell subtypes in the M1 region after SCI and rTMS intervention. a-i, Venn diagram showed the number of genes significantly different in SCI *vs* Sham and/or in SCI+rTMS *vs* SCI in M1 region, namely glut neurons (**a**), GABAergic neurons (**b**), OGDs (**c**), astrocytes (**d**), microglias (**e**), OPCs (**f**), fibroblasts (**g**), ECs (**h**), and pericytes (**i**)**.** Bubble plot showing top 3 enriched biological processes GO terms for genes with an upregulated-to-downregulated (**j**) and downregulated-to-upregulated (**k**) expression pattern across distinct cell

subtypes. A = Sham; B = SCI; C = SCI+rTMS. OGD, oligodendrocyte. OPC, oligodendrocyte precursor cell. EC endothelial cell.

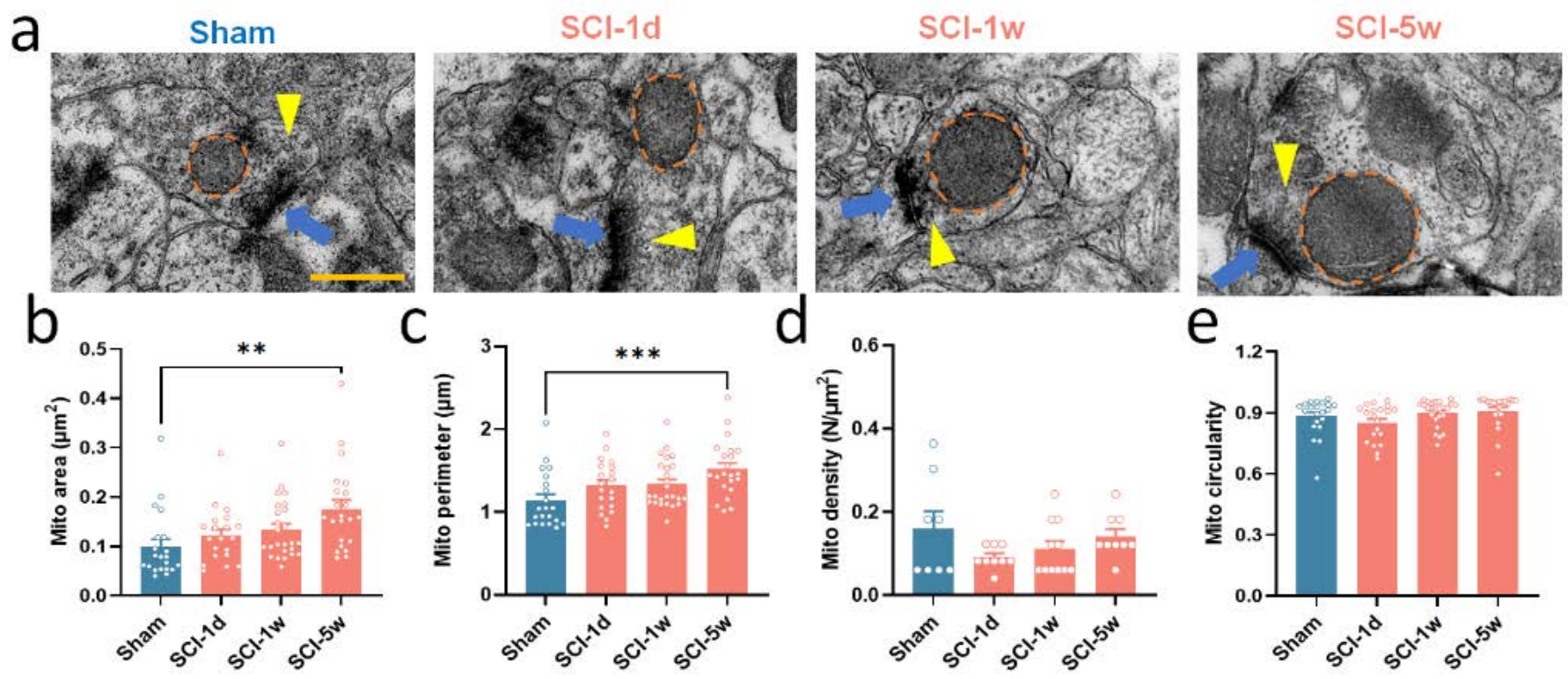

Supplementary Figure 4: Morphological results of Glut neuron mitochondria in Layer V of the M1 after SCI. a, TEM images of mitochondria in the M1 region of Sham and SCI mice at 1 day (SCI-1D), 1 week (SCI-1w), and 5 weeks (SCI-5w) post-injury. The orange dotted lines identify mitochondria, the blue arrows indicate synaptic structures (Gray I-type), and the golden triangles indicate the synaptic vesicles. Scale bars = 500 nm. **b-e,** Quantified by the area (**b**), perimeter (**c**), density (**d**), and circularity (**e**) of mitochondria in Sham (n = 21), SCI-1d (n = 20), SCI-1w (n = 25) and SCI-5w (n = 22) groups. *P < 0.05, ***P < 0.001.

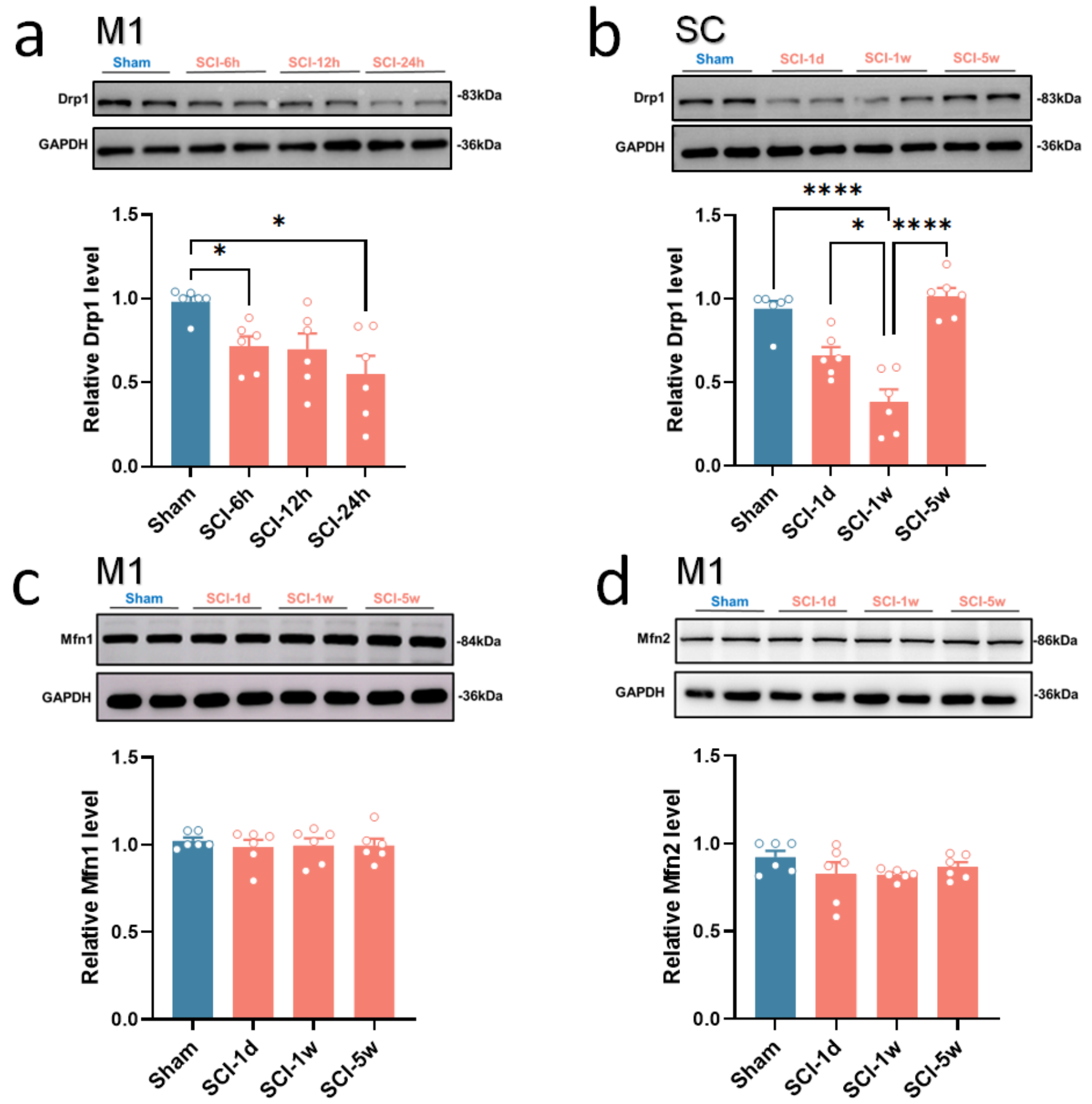

Supplementary Figure 5: The protein expression of Drp1 and Mfn1/2 of SCI mice. a, Western blot analysis of Drp1 protein expression in the M1 region of Sham and SCI mice at 6 hours (SCI-6h), 12 hours (SCI-12h), and 1d (SCI-24h) post-injury. And quantification normalized to GAPDH. **b,** Western blot analysis of Drp1 protein expression in the SC of Sham and SCI mice, and quantification normalized to GAPDH. **c,** Western blot analysis of Mfn1 protein expression in the M1 region of Sham and SCI mice, and quantification normalized to GAPDH. **d,** Western blot analysis of Mfn2 protein expression in the M1 region of Sham and SCI mice, and quantification normalized to GAPDH. $n = 6$ per group in all. *$P < 0.05$, ****$P < 0.0001$.

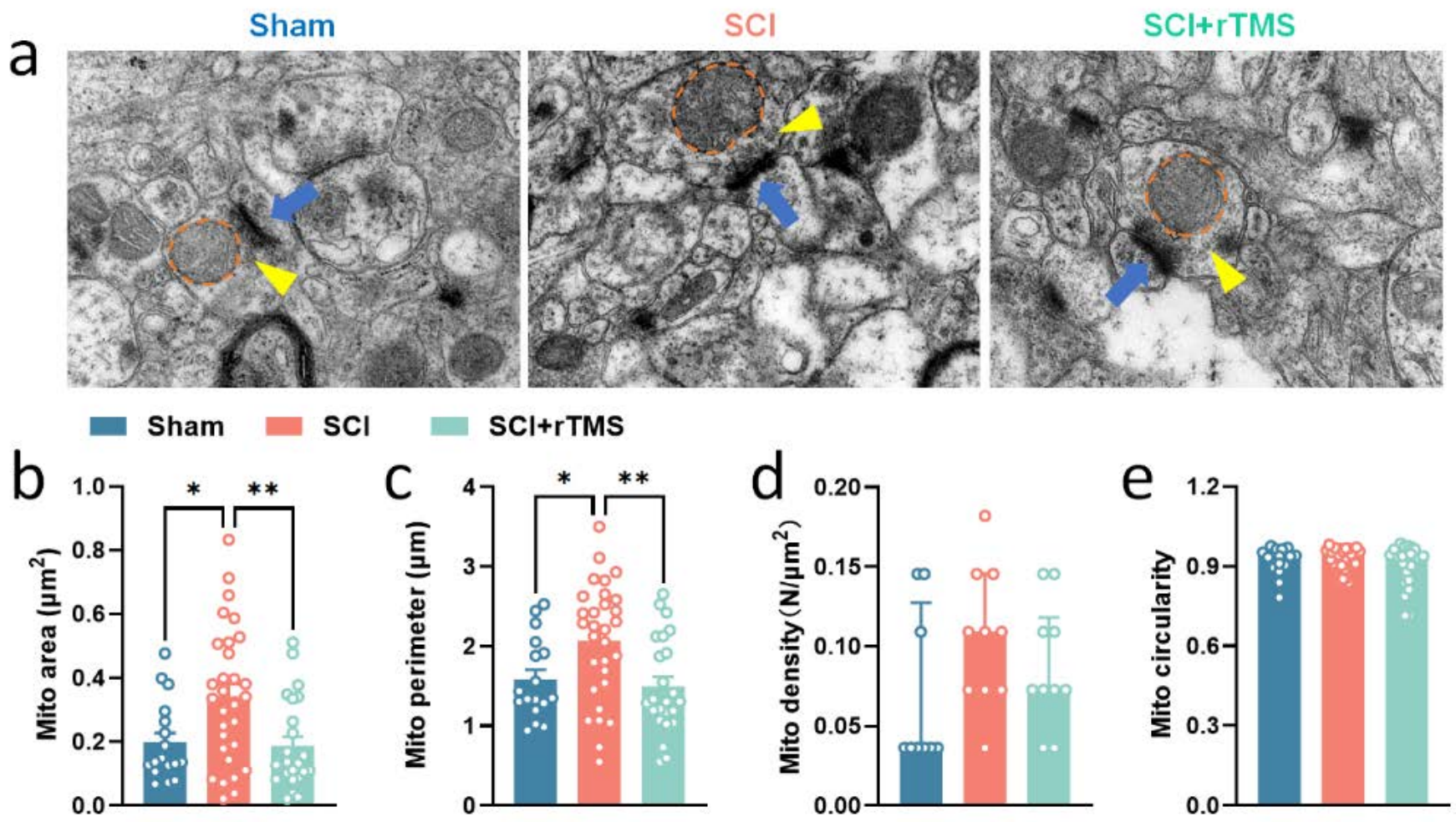

Supplementary Figure 6: Morphological results of Glut neuron mitochondria in Layer V of the M1. a, Representative TEM images showing the ultramicrostructure of Glut neurons in layer V of the M1 in Sham, SCI, and SCI+rTMS mice. The orange dotted lines indicate mitochondria, the blue arrows indicate synaptic structures (Gray I-type), and the golden triangles indicate the synaptic vesicles. Scale bars=500 nm. **b-e,** Quantitative analysis of mitochondrial parameters in Glut neurons. (**b**) mitochondrial area, (**c**) mitochondrial perimeter, (**d**) mitochondrial density, and (**e**) mitochondrial circularity in Sham (n = 17), SCI (n = 30), and SCI+rTMS (n = 24) groups. rTMS refers to HF-rTMS. *P < 0.05, **P < 0.01.

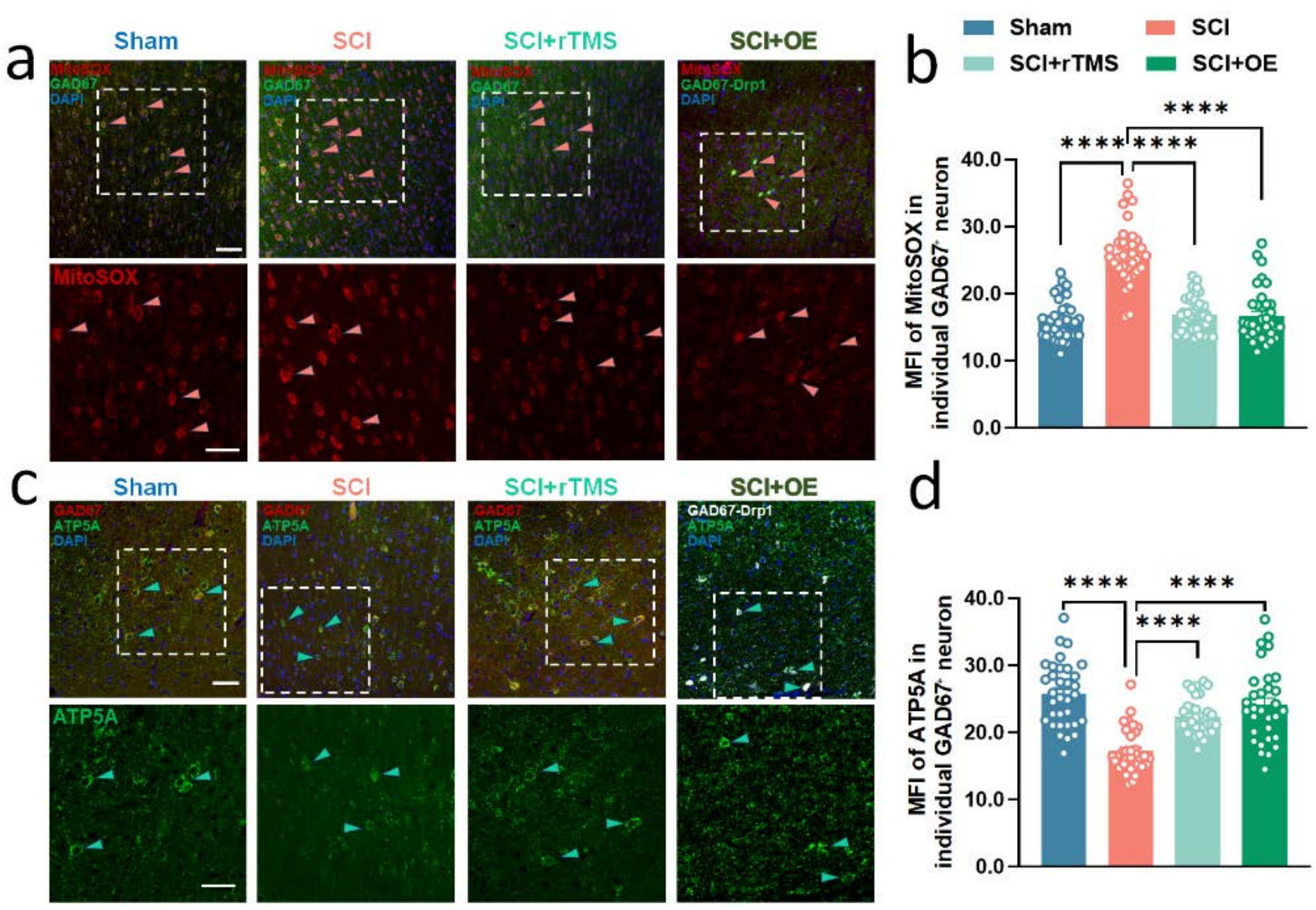

Supplementary Figure 7: Immunofluorescence staining and quantitative analysis of MitoSOX and ATP5A in GAD67^{+} neurons. a, Representative confocal images of layer V of the M1 brain region at 3 days post-SCI, showing MitoSOX (red, mitochondrial superoxide), GAD67 (green, inhibitory neurons), and DAPI (blue, cell nucleus). Arrowheads point to representative GAD67^{+} neurons. Four experimental groups are shown: Sham (n = 36), SCI (n = 36), SCI+rTMS (n = 40) and SCI+Drp1 overexpression (OE) group (n = 36). Scale bars = 100 μm, upper panels; Scale bars = 40 μm, lower panels. **b,** Quantitative analysis of the mean fluorescence intensity (MFI) of MitoSOX in individual GAD67^{+} neurons. **c,** Representative confocal images of layer V of the M1 brain region at 3 days post-SCI, showing ATP5A (green, mitochondrial marker), GAD67 (red pseudocolor was used for the Sham, SCI and SCI+rTMS groups, while white pseudocolor was applied to the SCI+OE group to visually separate it from the green GFP channel), and DAPI (blue, cell nucleus). Arrowheads point to representative GAD67^{+} neurons. Four experimental groups are shown: Sham (n = 33), SCI (n = 35), SCI+rTMS (n = 33) and SCI+Drp1 overexpression (OE) groups (n = 31). Scale bars = 100 μm, upper panels; Scale bars = 40 μm, lower panels. **d,** Quantitative analyze of the MFI of ATP5A in individual GAD67^{+} neurons. rTMS refers to HF-rTMS. *$P < 0.05$, **$P < 0.01$, ****$P < 0.0001$.

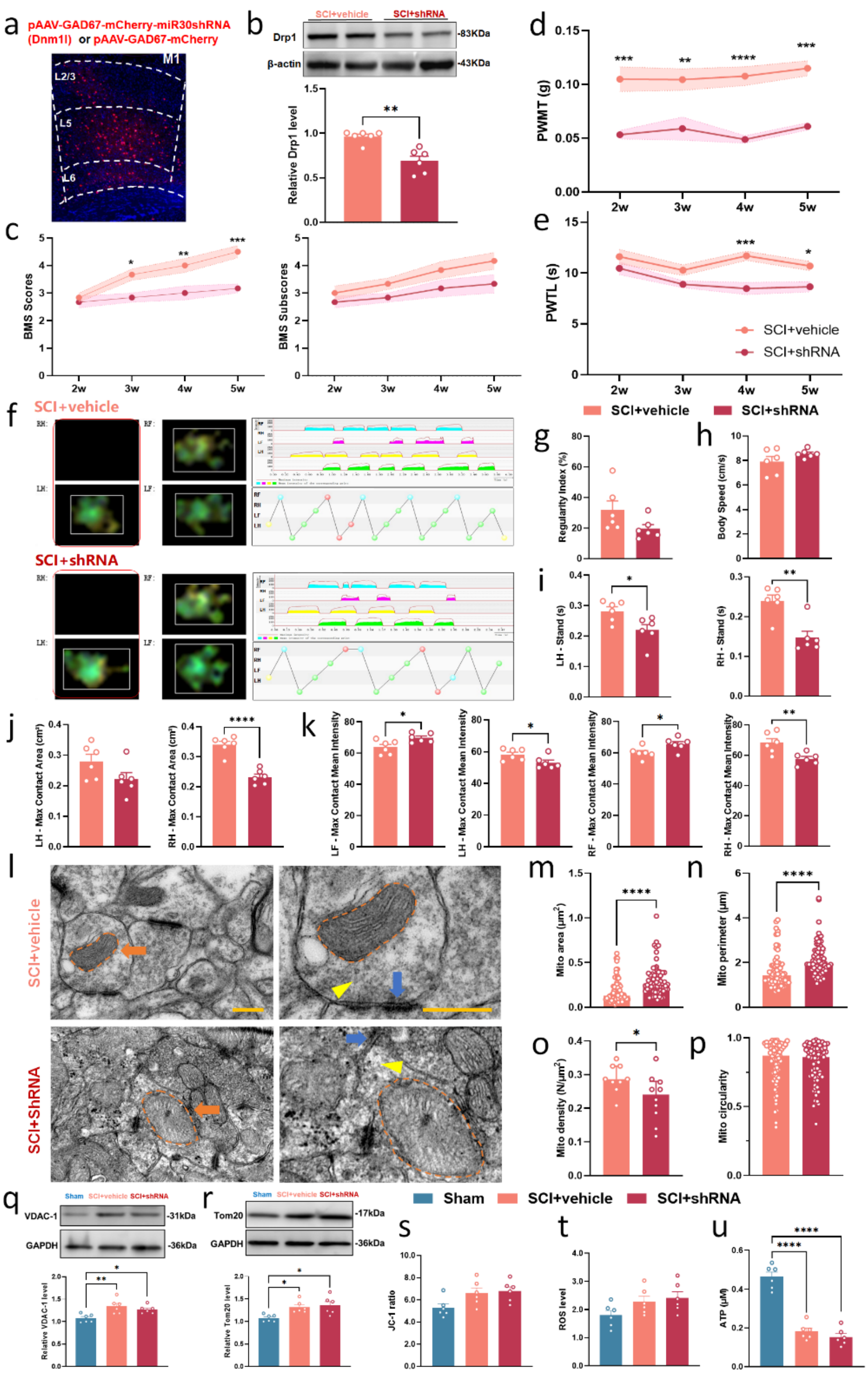

Supplementary Figure 8: Effects of down-regulation Drp1 in GABAergic neuron of M1 Layer V on mitochondrial morphology and function. a, Schematic representation of an IF image showing the expression of the mCherry marker in the layer V of M1 region. Scale bars = 200 μm. **b,** Western blot analysis of Drp1 protein expression in the M1 region of mice treated with a vehicle or shRNA (up). Quantitative analysis of the relative protein levels of Drp1, normalized to β-actin (down) (*n* = 6 per group). **c,** BMS scores and subscores evaluating locomotor function in

SCI+vehicle and SCI+shRNA mice (*n* = 6 per group). **d,e,** Assessment of mechanical allodynia (**d**) and thermal hyperalgesia (**e**) in SCI+vehicle and SCI+shRNA mice (*n* = 6 per group). **f-k,** Representative Catwalk gait images of SCI+vehicle and SCI+shRNA mice at 5 weeks post-SCI, as measured by the regularity index (**g**), body speed (**h**), the stand time (**i**), max paw contact area (**j**) of hind paw prints and the max contact mean intensity of paw prints (**k**) (*n* = 6 mice per group). **l,** TEM images of mitochondria in the M1 region of SCI+vehicle and SCI+shRNA mice. Scale bars = 500 nm. The orange arrows and dotted lines identify mitochondria, the blue arrows identify the synaptic structure of Gray II-type, and the golden triangles identify synaptic vesicles. **m-p,** Quantified by the area (**m**), perimeter (**n**), density (**o**), and circularity (**p**) of mitochondria in SCI+vehicle (*n* = 71) and SCI+shRNA (*n* = 70) groups. Samples were obtained from multiple ultrathin sections of 6 biologically independent mice per group. **q,r,** Western blot analysis and quantification of VDAC-1 (**q**) and Tom20 (**r**) protein expression in the M1 region, normalized to GAPDH (*n* = 6 per group). **s-u,** Quantification of mitochondrial function (*n* = 6 per group), including JC-1 ratio (**s**), ROS level (**t**), and ATP (**u**). *$P < 0.05$, **$P < 0.01$, ***$P < 0.001$ and ****$P < 0.0001$.

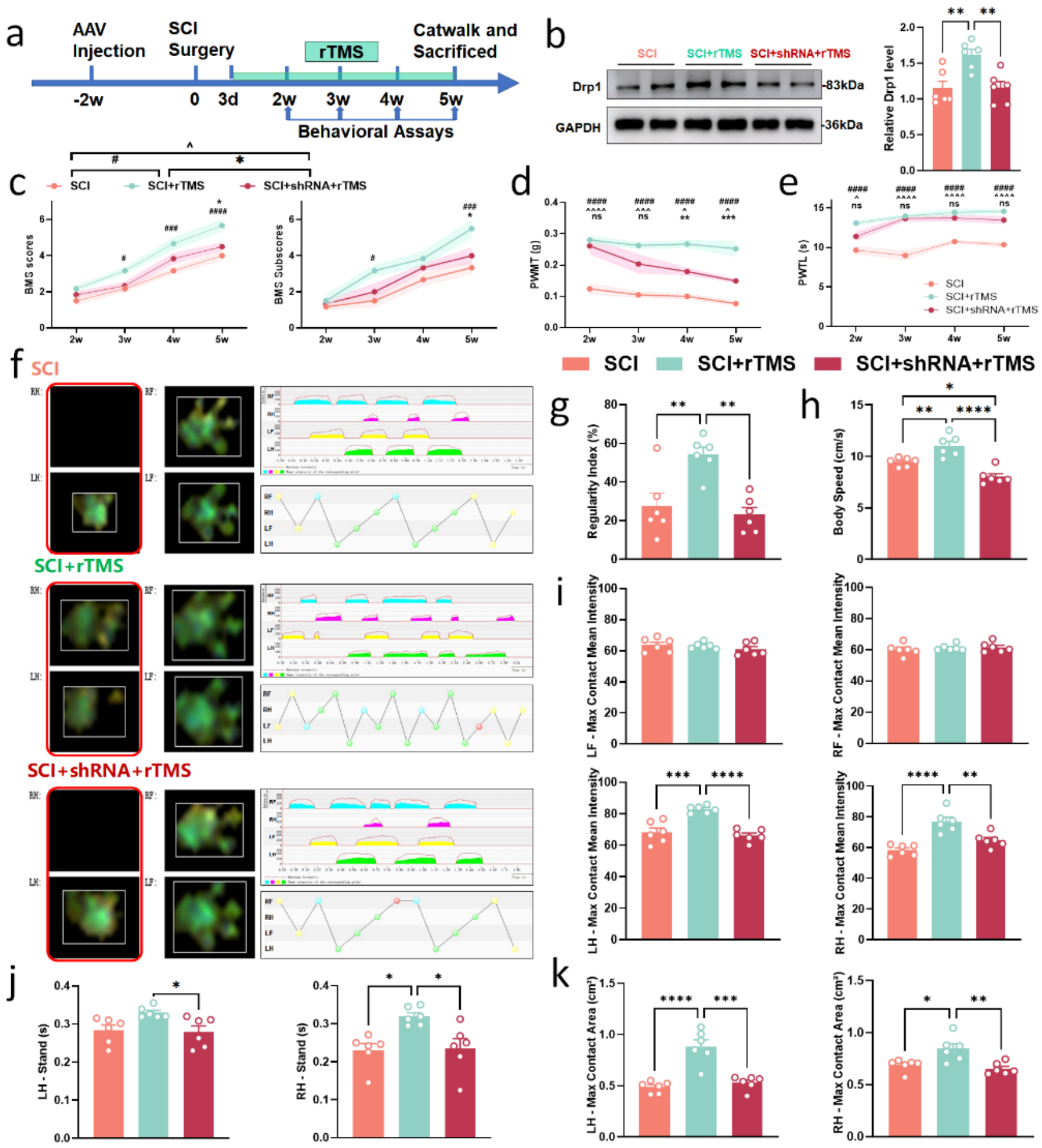

Supplementary Figure 9: The therapeutic effect of HF-rTMS depends on the Drp1 of M1 layer V GABAergic neuron. a, Schematic representation of the experimental paradigm. **b,** Western blot analysis of Drp1 protein expression in the M1 region of SCI, SCI+rTMS, and SCI+shRNA+rTMS mice (left). Quantitative analysis of the relative protein levels of Drp1, normalized to GAPDH (right). **c,** Basso Mouse Scale (BMS) scores evaluating locomotor function in SCI, SCI+rTMS, and SCI+shRNA+rTMS mice at various time points post-SCI. **d,e,** Assessment of mechanical allodynia (**d**) and thermal hyperalgesia (**e**) in SCI mice with or without rTMS and shRNA treatment. **f,** The typical mouse paw print in Catwalk, where the red box is the hind paw print. **g-k**, Quantification of Catwalk gait analysis in SCI, SCI+rTMS, and SCI+shRNA+rTMS mice at 5 weeks post-SCI, as measured by the regularity index (**g**), body speed (**h**), the mean intensity of paw prints (**i**), the stand time (**j**) and max paw contact area (**k**) of hind paw prints. Line graphs (**c-e**) use # (SCI *vs* SCI+rTMS), ^ (SCI *vs* SCI+shRNA+rTMS), * (SCI+rTMS *vs* SCI+shRNA+rTMS).

Graded symbols correspond to $P < 0.05$, 0.01, 0.001, 0.0001. ns = not significant. $n = 6$ per group in all. rTMS refers to HF-rTMS. *$P < 0.05$, **$P < 0.01$, ***$P < 0.001$ and ****$P < 0.0001$.

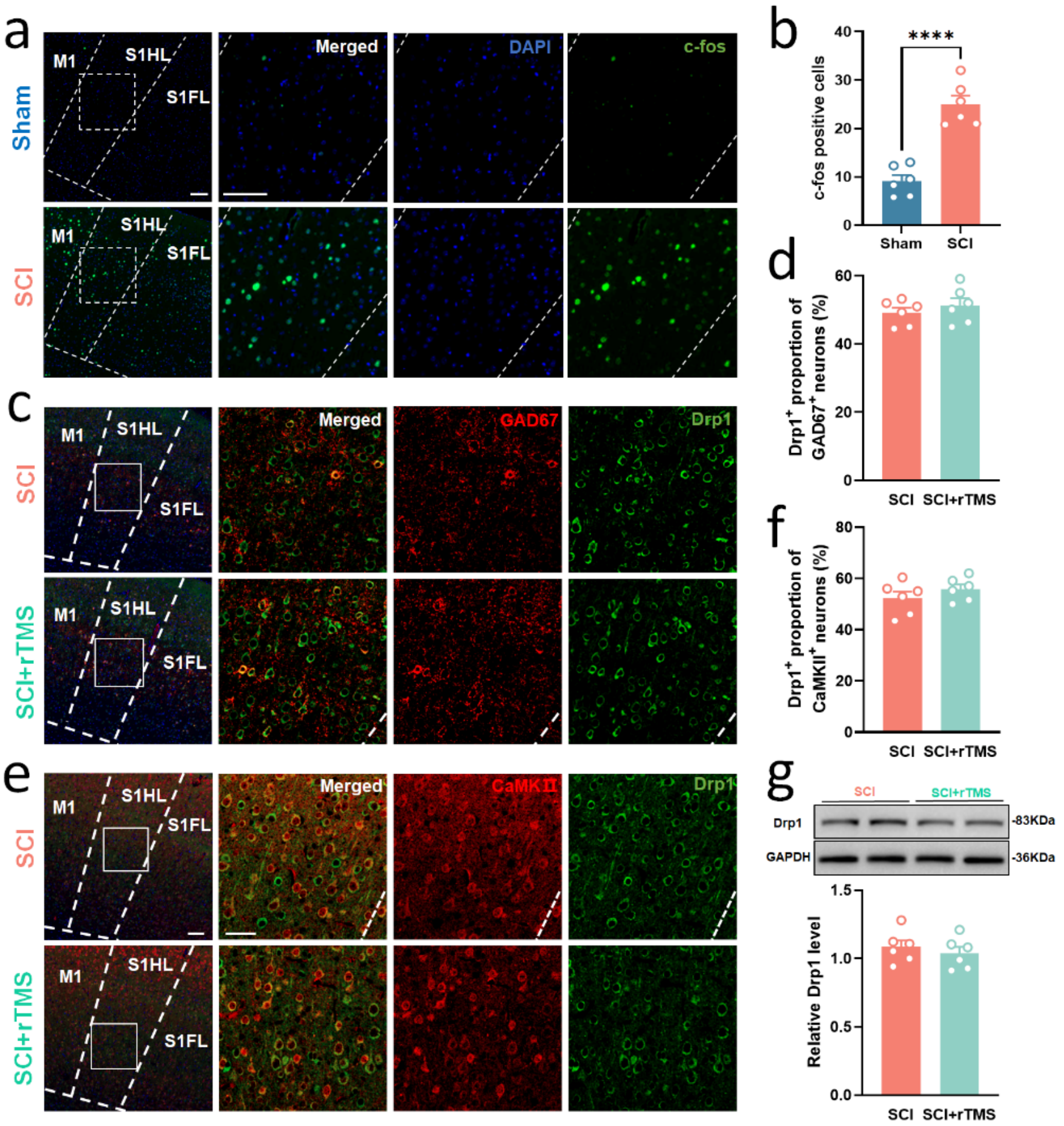

Supplementary Figure 10: The expression of Drp1 in S1HL did not change after rTMS. a,b, Representative confocal images of fos positive neurons (**a**) and quantitative analysis (**b**) at 5 weeks post-SCI (Scale bars = 100 μm, left. Scale bars = 20 μm, right). **c-f,** Representative confocal images of CaMKII-Drp1 positive neurons (**c**) and GAD67-Drp1 positive neurons (**e**) at 5 weeks post-SCI (Scale bars = 100 μm, left. Scale bars = 20 μm, right). Quantitative analysis of CaMKII-Drp1 positive neurons (**d**) and GAD67-Drp1 positive neurons (**f**) ($n = 6$ per group). The fluorescent slices obtained from 6 mice per group. **g,** Western blot analysis of the effect of rTMS on Drp1 protein expression in S1HL region of SCI mice (up). Quantitative analysis of the relative protein levels of Drp1, normalized to GAPDH (down) ($n = 6$ per group). rTMS refers to HF-rTMS. ****$P < 0.0001$.

Supplementary Table 1. Basic characteristics of SCI and SCI+rTMS patients

	SCI<br>*n* = 53	SCI+rTMS<br>*n* = 40	*P*
Age, y, mean (SD) [a]	40.96±10.19	39.39±10.45	0.2156
Gender, n (%) [b]			
Male	49 (92.45)	35 (87.50)	0.6559
Female	4 (7.55)	5 (12.50)	
Injury severity, n (%) [c]			
Complete	17 (32.08)	16 (40.00)	0.4291
Incomplete	36 (67.92)	24 (60.00)	
Injury level, n (%) [d]			
Cervical	14 (26.42)	14 (35.00)	0.5402
Thoracic	26 (49.06)	14 (35.00)	
Lumbar	11 (20.75)	11 (27.50)	
Sacral	2 (3.77)	1 (2.50)	
MBI (QR) [e]	31.62 (16.00, 45.00)	36.60 (16.00, 49.75)	0.4335
NRS (QR) [e]	3.57 (2.00, 5.00)	3.90 (3.00, 5.00)	0.4078
MMT (QR) [e]	47.21 (47.50, 61.00)	48.70 (40.00, 59.25)	0.3810
Analgesic medication, n (%) [d]			
Pregabalin	9 (16.98)	11 (27.50)	0.1226
Oxycodone & Acetaminophen	0 (0.00)	1 (2.50)	
Venlafaxine	0 (0.00)	1 (2.50)	
No analgesic	44 (83.02)	27 (67.50)	

Abbreviations: SCI, spinal cord injury; rTMS, repetitive transcranial magnetic stimulation; y, year; SD, standard deviation; QR, quartile range; MBI, modified Barthel index; NRS, numerical rating scale; MMT, manual muscle test.

[a] Student's t test

[b] Chi-square test with Yates' correction

[c] Chi-square test

[d] Fisher's exact test

[e] Mann-Whitney U test

Supplementary Table 2. TOP10 GO enrichment in biological processes in Astrocyte

Astrocyte	Description	Pvalue	Qvalue	fg_num	bg_num
down_up	nervous system development	1.44E-11	2.47E-08	40	2536
	chemical synaptic transmission	3.19E-11	2.47E-08	22	762
	anterograde trans-synaptic signaling	3.19E-11	2.47E-08	22	762
	trans-synaptic signaling	3.90E-11	2.47E-08	22	770
	modulation of chemical synaptic transmission	9.32E-11	3.82E-08	19	577
	regulation of trans-synaptic signaling	9.60E-11	3.82E-08	19	578
	synaptic signaling	1.05E-10	3.82E-08	22	811
	generation of neurons	7.74E-09	2.46E-06	28	1637
	neuron differentiation	1.09E-08	2.79E-06	27	1553
	neurogenesis	1.10E-08	2.79E-06	30	1891
up_down	pteridine-containing compound metabolic process	0.0002277	0.067867	2	31
	positive regulation of mitotic cell cycle, embryonic	0.0007288	0.067867	1	1
	regulation of stomach neuroendocrine cell differentiation	0.0007288	0.067867	1	1
	negative regulation of stomach neuroendocrine cell differentiation	0.0007288	0.067867	1	1
	regulation of cell fate determination	0.0007288	0.067867	1	1
	negative regulation of cell fate determination	0.0007288	0.067867	1	1
	regulation of pancreatic A cell differentiation	0.0007288	0.067867	1	1
	negative regulation of pancreatic A cell differentiation	0.0007288	0.067867	1	1
	glandular epithelial cell differentiation	0.0013694	0.067867	2	76
	activation of plasma proteins involved in acute inflammatory response	0.001457	0.067867	1	2

Supplementary Table 3. TOP10 GO enrichment in biological processes in EC

EC	Description	Pvalue	Qvalue	fg_num	bg_num
down_up	kidney development	0.00037	0.0962772	3	347
	renal system development	0.0004088	0.0962772	3	359
	post-transcriptional tethering of RNA polymerase II gene DNA at nuclear periphery	0.0008743	0.0962772	1	2
	co-translational protein modification	0.0013113	0.0962772	1	3
	female gonad development	0.0013633	0.0962772	2	129
	development of primary female sexual characteristics	0.0014482	0.0962772	2	133
	male germ-line stem cell asymmetric division	0.001748	0.0962772	1	4
	positive regulation of NK T cell differentiation	0.001748	0.0962772	1	4
	positive regulation of blood-brain barrier permeability	0.001748	0.0962772	1	4
	female sex differentiation	0.0017885	0.0962772	2	148
up_down	neuron projection development	0.0002277	0.067867	2	31
	cellular anatomical entity morphogenesis	0.0007288	0.067867	1	1
	neuron projection morphogenesis	0.0007288	0.067867	1	1
	plasma membrane bounded cell projection morphogenesis	0.0007288	0.067867	1	1
	cell projection morphogenesis	0.0007288	0.067867	1	1
	neurogenesis	0.0007288	0.067867	1	1
	cell morphogenesis involved in neuron differentiation	0.0007288	0.067867	1	1
	neuron development	0.0007288	0.067867	1	1
	neuron differentiation	0.0013694	0.067867	2	76
	axonogenesis	0.001457	0.067867	1	2

Supplementary Table 4. TOP10 GO enrichment in biological processes in Fibroblast

Fibroblast	Description	Pvalue	Qvalue	fg_num	bg_num
**down_up**	neuron projection development	3.22E-08	1.52E-05	16	1131
	cellular anatomical entity morphogenesis	3.33E-08	1.52E-05	14	832
	neuron projection morphogenesis	3.40E-08	1.52E-05	13	697
	plasma membrane bounded cell projection morphogenesis	4.36E-08	1.52E-05	13	712
	cell projection morphogenesis	4.89E-08	1.52E-05	13	719
	neurogenesis	5.09E-08	1.52E-05	20	1891
	cell morphogenesis involved in neuron differentiation	1.18E-07	3.00E-05	12	640
	neuron development	1.76E-07	3.92E-05	16	1279
	neuron differentiation	4.39E-07	8.54E-05	17	1553
	axonogenesis	5.16E-07	8.54E-05	10	473
**up_down**	organ growth	9.87E-06	0.0140572	5	201
	negative regulation of organ growth	2.10E-05	0.0149481	3	37
	extracellular matrix organization	7.61E-05	0.0223542	5	308
	external encapsulating structure organization	7.61E-05	0.0223542	5	308
	extracellular structure organization	7.85E-05	0.0223542	5	310
	skeletal system development	9.94E-05	0.0235889	6	530
	bone development	0.000348	0.0418106	4	233
	negative regulation of developmental growth	0.0004352	0.0418106	3	102
	cardiac muscle cell development	0.0004871	0.0418106	3	106
	cardiac muscle tissue growth	0.0005007	0.0418106	3	107

Supplementary Table 5. TOP10 GO enrichment in biological processes in Glut

Glut	Description	Pvalue	Qvalue	fg_num	bg_num
down_up	oxidative phosphorylation	1.84E-13	1.07E-10	16	1131
	ATP synthesis coupled electron transport	3.15E-13	1.07E-10	14	832
	proton transmembrane transport	4.58E-13	1.07E-10	13	697
	respiratory electron transport chain	2.23E-12	3.59E-10	13	712
	aerobic respiration	2.56E-12	3.59E-10	13	719
	electron transport chain	4.54E-12	5.31E-10	20	1891
	cellular respiration	1.56E-11	1.49E-09	12	640
	aerobic electron transport chain	1.70E-11	1.49E-09	16	1279
	monoatomic ion transport	2.77E-11	2.15E-09	17	1553
	mitochondrial ATP synthesis coupled electron transport	4.36E-11	3.06E-09	10	473
up_down	regulation of protein kinase C activity	0.0005344	0.1138356	1	1
	positive regulation of protein kinase C activity	0.0005344	0.1138356	1	1
	cellular response to 1-oleoyl-sn-glycerol 3-phosphate	0.0005344	0.1138356	1	1
	positive regulation of calcium ion transmembrane transport	0.0008221	0.1138356	2	81
	esophagus smooth muscle contraction	0.0010686	0.1138356	1	2
	response to 1-oleoyl-sn-glycerol 3-phosphate	0.0010686	0.1138356	1	2
	nerve development	0.0012001	0.1138356	2	98
	positive regulation of calcium ion transport	0.0016753	0.1181991	2	116
	positive regulation of protein localization to membrane	0.0016753	0.1181991	2	116
	positive regulation of cation transmembrane transport	0.0018506	0.1181991	3	107

Supplementary Table 6. TOP10 GO enrichment in biological processes in GABA

GABA	Description	Pvalue	Qvalue	fg_num	bg_num
**down_up**	oxidative phosphorylation	1.78E-06	0.0014794	6	143
	ATP synthesis coupled electron transport	1.94E-06	0.0014794	5	80
	proton transmembrane transport	3.41E-06	0.0017352	6	160
	respiratory electron transport chain	7.41E-06	0.0026296	5	105
	nervous system development	9.07E-06	0.0026296	19	2536
	aerobic respiration	1.16E-05	0.0026296	6	198
	electron transport chain	1.21E-05	0.0026296	5	116
	aerobic electron transport chain	2.66E-05	0.0044124	4	67
	neuron differentiation	2.67E-05	0.0044124	14	1553
	inorganic cation transmembrane transport	2.89E-05	0.0044124	10	791
**up_down**	chaperone-mediated protein folding	6.85E-06	0.0086167	4	65
	chaperone cofactor-dependent protein refolding	2.72E-05	0.0171265	3	31
	response to nitrogen compound	5.91E-05	0.0198435	10	1201
	'de novo' post-translational protein folding	6.86E-05	0.0198435	3	42
	'de novo' protein folding	7.89E-05	0.0198435	3	44
	response to organonitrogen compound	0.0001462	0.0306271	9	1078
	organic hydroxy compound transport	0.0002223	0.0399102	5	294
	negative regulation of multicellular organism growth	0.0003138	0.0493052	2	14
	protein folding	0.0003587	0.0500917	4	179
	vitamin transmembrane transport	0.0007181	0.0902654	2	21

Supplementary Table 7. TOP10 GO enrichment in biological processes in Microglial

Microglial	Description	Pvalue	Qvalue	fg_num	bg_num
**down_up**	chemical synaptic transmission	1.09E-06	0.0006419	10	762
	anterograde trans-synaptic signaling	1.09E-06	0.0006419	10	762
	trans-synaptic signaling	1.20E-06	0.0006419	10	770
	synaptic signaling	1.92E-06	0.0007686	10	811
	regulation of biological quality	2.73E-06	0.0008762	18	3027
	neuron projection morphogenesis	4.74E-06	0.0008873	9	697
	NADH regeneration	5.03E-06	0.0008873	3	18
	canonical glycolysis	5.03E-06	0.0008873	3	18
	glucose catabolic process to pyruvate	5.03E-06	0.0008873	3	18
	plasma membrane bounded cell projection morphogenesis	5.63E-06	0.0008873	9	712
**up_down**	regulation of G1/S transition of mitotic cell cycle	0.0010392	0.0888973	2	147
	positive regulation of endothelial cell-matrix adhesion via fibronectin	0.0013598	0.0888973	1	4
	regulation of cell cycle G1/S phase transition	0.0014183	0.0888973	2	172
	endothelial cell-matrix adhesion via fibronectin	0.0016994	0.0888973	1	5
	regulation of endothelial cell-matrix adhesion via fibronectin	0.0016994	0.0888973	1	5
	G1/S transition of mitotic cell cycle	0.0017991	0.0888973	2	194
	cell cycle G1/S phase transition	0.002326	0.0888973	2	221
	endothelial cell-matrix adhesion	0.0023785	0.0888973	1	7
	single-stranded RNA binding	0.0309293	0.092788	1	92
	positive regulation of cyclin-dependent protein kinase activity	0.0033964	0.0964862	1	10

Supplementary Table 8. TOP10 GO enrichment in biological processes in OGD

OGD	Description	Pvalue	Qvalue	fg_num	bg_num
**down_up**	meiotic spindle elongation	0.0007288	0.1808617	1	1
	methylglyoxal biosynthetic process	0.001457	0.1808617	1	2
	slow endocytic recycling	0.001457	0.1808617	1	2
	positive regulation of CAMKK-AMPK signaling cascade	0.001457	0.1808617	1	2
	regulation of meiotic cell cycle process involved in oocyte maturation	0.0021848	0.1808617	1	3
	meiotic sister chromatid cohesion, centromeric	0.0029121	0.1808617	1	4
	negative regulation of complement-dependent cytotoxicity	0.0029121	0.1808617	1	4
	neuron differentiation	0.0038429	0.1808617	5	1553
	virion attachment to host cell	0.0043651	0.1808617	1	6
	adhesion of symbiont to host cell	0.0043651	0.1808617	1	6
**up_down**	embryonic digit morphogenesis	0.0003703	0.0658988	2	67
	positive regulation of presynaptic membrane organization	0.0004373	0.0658988	1	1
	regulation of presynaptic membrane organization	0.0008743	0.0658988	1	2
	response to antineoplastic agent	0.0013113	0.0658988	1	3
	regulation of ossification	0.0014482	0.0658988	2	133
	embryonic limb morphogenesis	0.0014482	0.0658988	2	133
	embryonic appendage morphogenesis	0.0014482	0.0658988	2	133
	male germ-line stem cell asymmetric division	0.001748	0.0658988	1	4
	positive regulation of NK T cell differentiation	0.001748	0.0658988	1	4
	appendage morphogenesis	0.0021374	0.0658988	2	162

Supplementary Table 9. TOP10 GO enrichment in biological processes in OPC

OPC	Description	Pvalue	Qvalue	fg_num	bg_num
**down_up**	nervous system development	2.12E-07	0.000194	20	2536
	localization	2.31E-07	0.000194	29	5319
	establishment of localization	4.78E-06	0.0012313	25	4649
	modulation of chemical synaptic transmission	5.29E-06	0.0012313	9	577
	regulation of trans-synaptic signaling	5.36E-06	0.0012313	9	578
	regulation of signaling	5.38E-06	0.0012313	21	3397
	regulation of cell communication	5.43E-06	0.0012313	21	3399
	chemical synaptic transmission	6.81E-06	0.0012313	10	762
	anterograde trans-synaptic signaling	6.81E-06	0.0012313	10	762
	trans-synaptic signaling	7.47E-06	0.0012313	10	770
**up_down**	intracellular protein transmembrane transport	2.17E-05	0.0316877	3	33
	positive regulation of mononuclear cell migration	0.0002272	0.0672544	3	72
	protein transmembrane transport	0.0002564	0.0672544	3	75
	protein import into peroxisome matrix	0.0002744	0.0672544	2	15
	positive regulation of lymphocyte chemotaxis	0.000445	0.0672544	2	19
	cell motility	0.0004639	0.0672544	10	1796
	nervous system development	0.0004771	0.0672544	12	2536
	regulation of leukocyte migration	0.0004819	0.0672544	4	223
	protein localization to peroxisome	0.0004939	0.0672544	2	20
	establishment of protein localization to peroxisome	0.0004939	0.0672544	2	20

Supplementary Table 10. TOP10 GO enrichment in biological processes in Pericytes

Pericytes	Description	Pvalue	Qvalue	fg_num	bg_num
**down_up**	renal filtration cell differentiation	0.0022337	0.0518091	1	23
	podocyte differentiation	0.0022337	0.0518091	1	23
	glomerular epithelial cell differentiation	0.0023307	0.0518091	1	24
	glomerular epithelium development	0.0026219	0.0518091	1	27
	negative regulation of DNA-templated transcription	0.0040046	0.0518091	2	1303
	negative regulation of RNA biosynthetic process	0.0040539	0.0518091	2	1311
	epithelial cell differentiation involved in kidney development	0.0045618	0.0518091	1	47
	negative regulation of RNA metabolic process	0.0047497	0.0518091	2	1419
	negative regulation of fat cell differentiation	0.0054341	0.0518091	1	56
	cell differentiation involved in kidney development	0.0054341	0.0518091	1	56
**up_down**	organelle assembly	0.0006899	0.1222242	6	1014
	cellular component assembly	0.0007867	0.1222242	10	3000
	thymidine catabolic process	0.0011174	0.1222242	1	1
	pyrimidine deoxyribonucleoside catabolic process	0.0011174	0.1222242	1	1
	detection of mechanical stimulus involved in equilibrioception	0.0011174	0.1222242	1	1
	entry receptor-mediated virion attachment to host cell	0.0011174	0.1222242	1	1
	positive regulation of intracellular transport of viral material	0.0011174	0.1222242	1	1
	cellular component biogenesis	0.0015372	0.1222242	10	3263
	angiotensin-mediated drinking behavior	0.0022337	0.1222242	1	2
	uracil catabolic process	0.0022337	0.1222242	1	2
