# Supplementary material for: Drp1 in M1 layer V GABAergic neurons orchestrates rTMS-mediated motor restoration and analgesia after spinal cord injury
Source: Exp Mol Med. 2026 Oct 5;58(10):3440–56. doi: 10.1038/s12276-026-01857-2 (PMC13737150; doi:10.1038/s12276-026-01857-2)

Full unedited blot/gel for Figure [4. f]

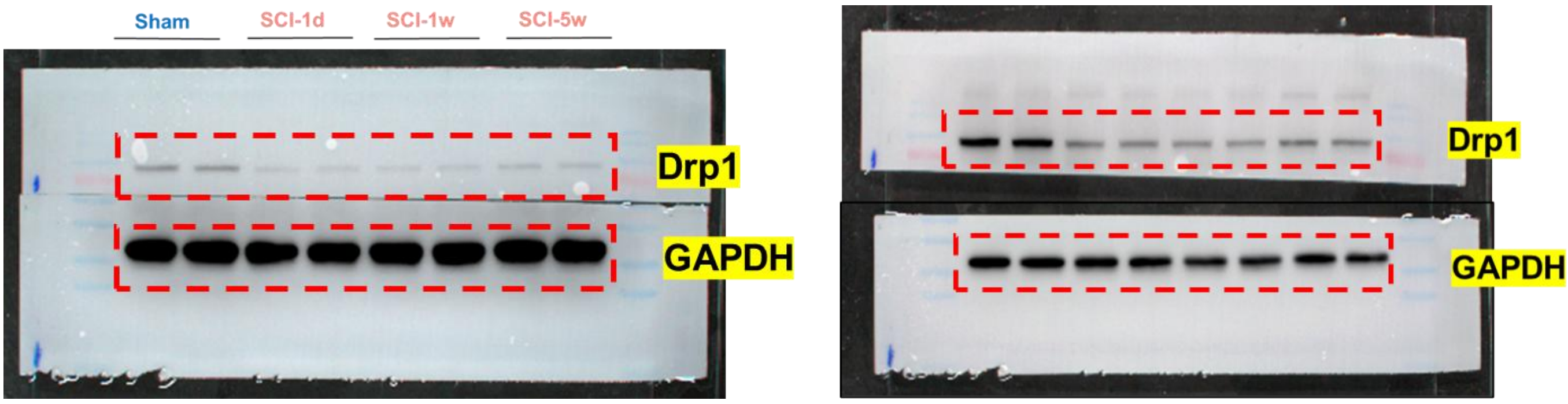

Full unedited blot/gel for Figure [4. h]

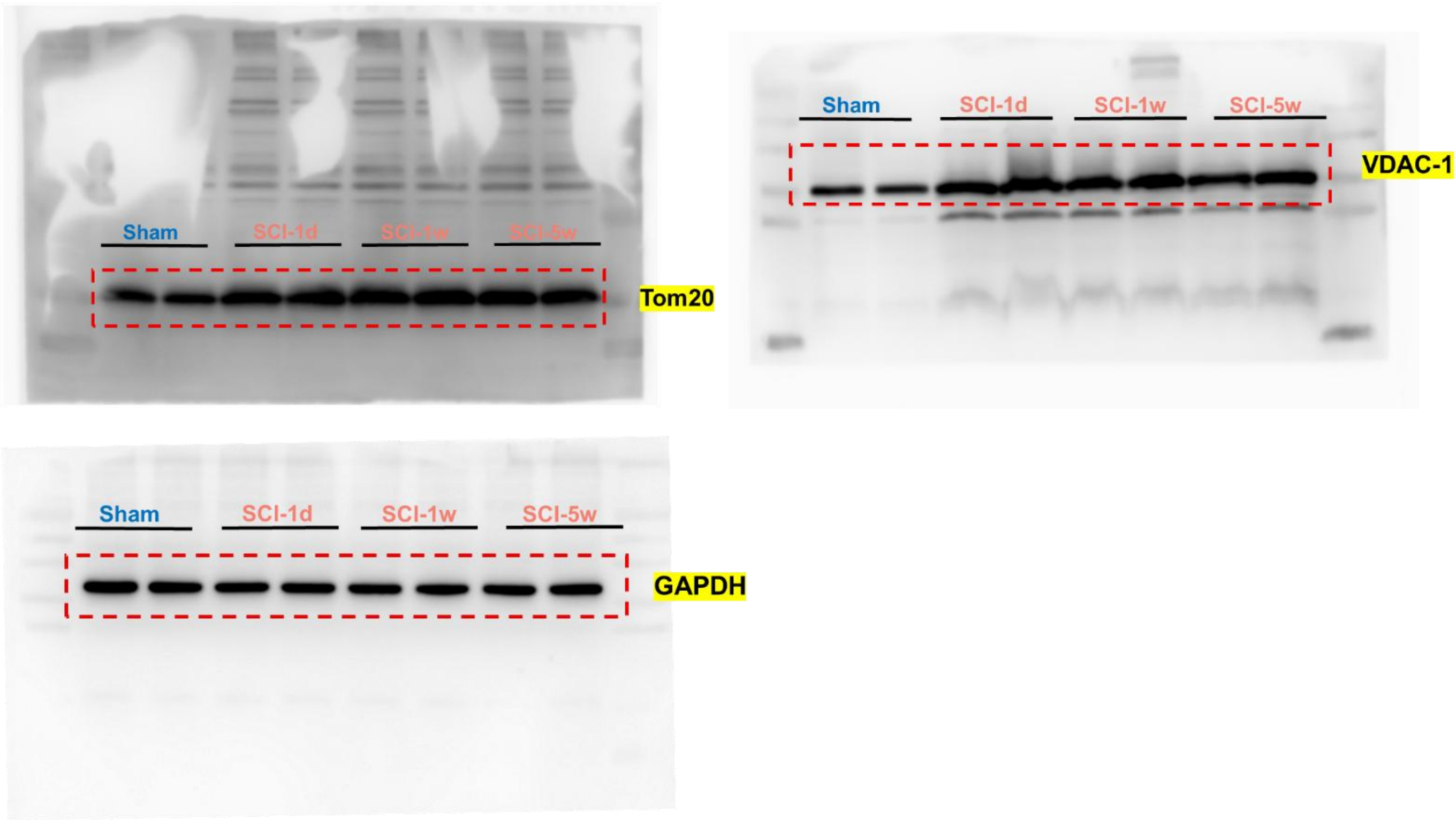

Full unedited blot/gel for Figure [5. b]

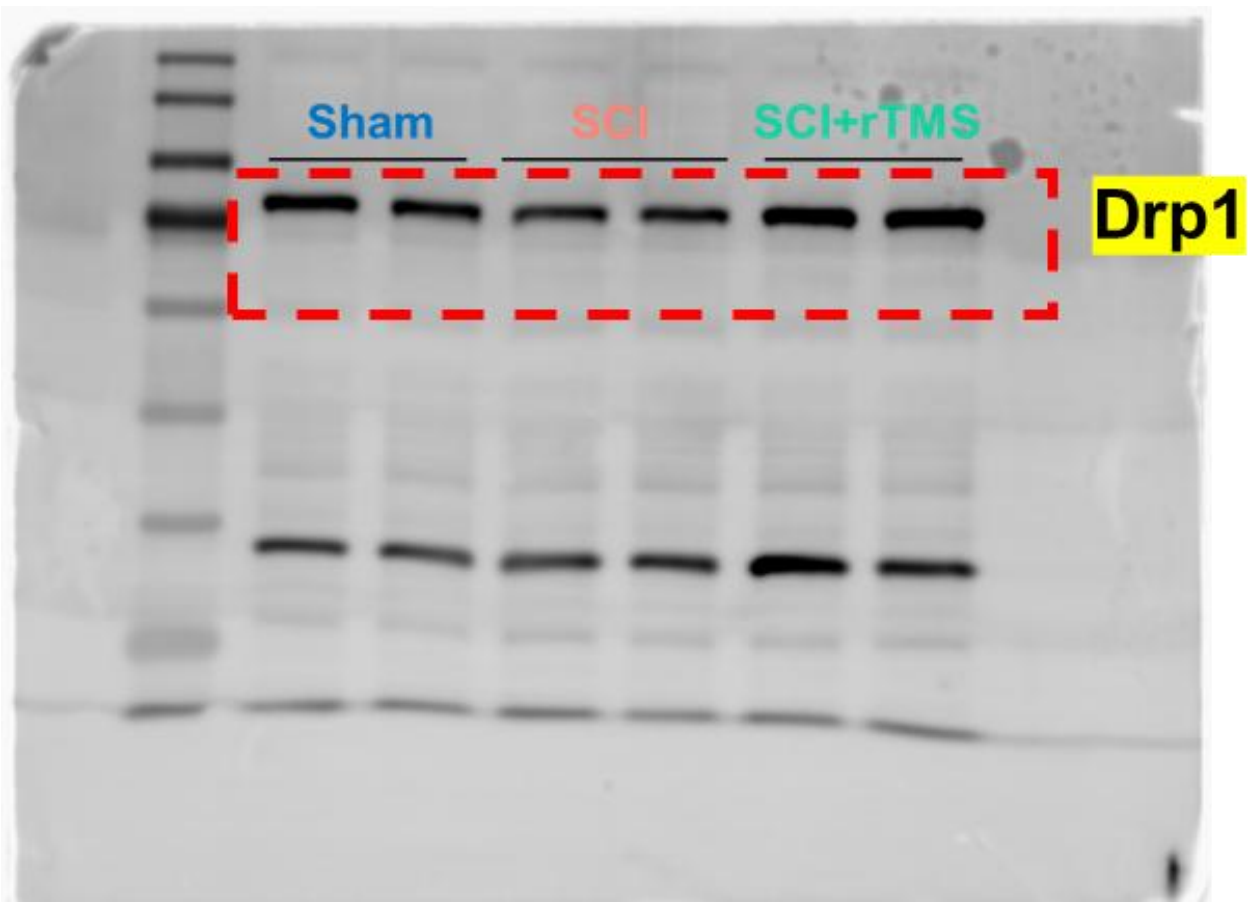

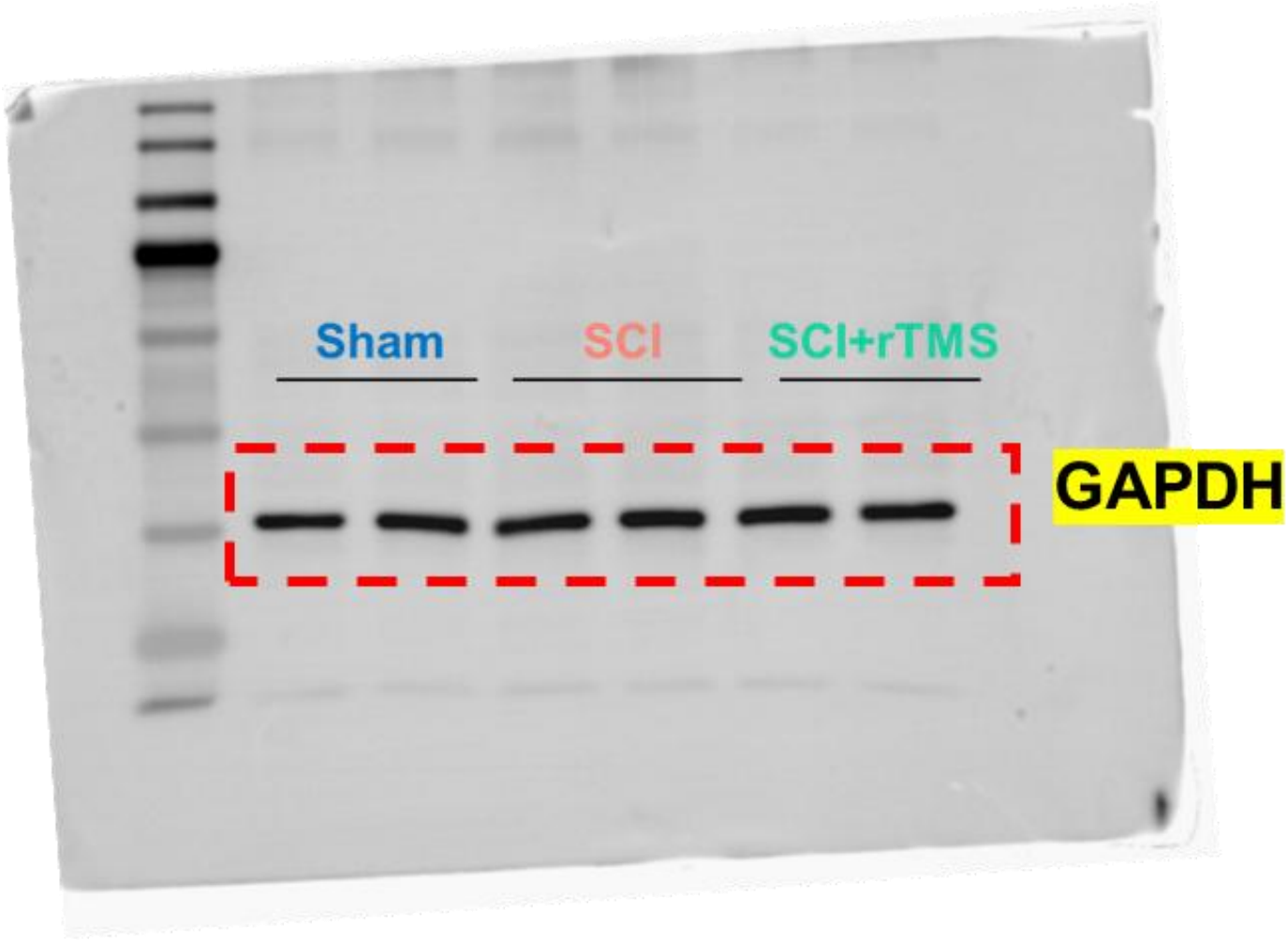

Full unedited blot/gel for Figure [7. c]

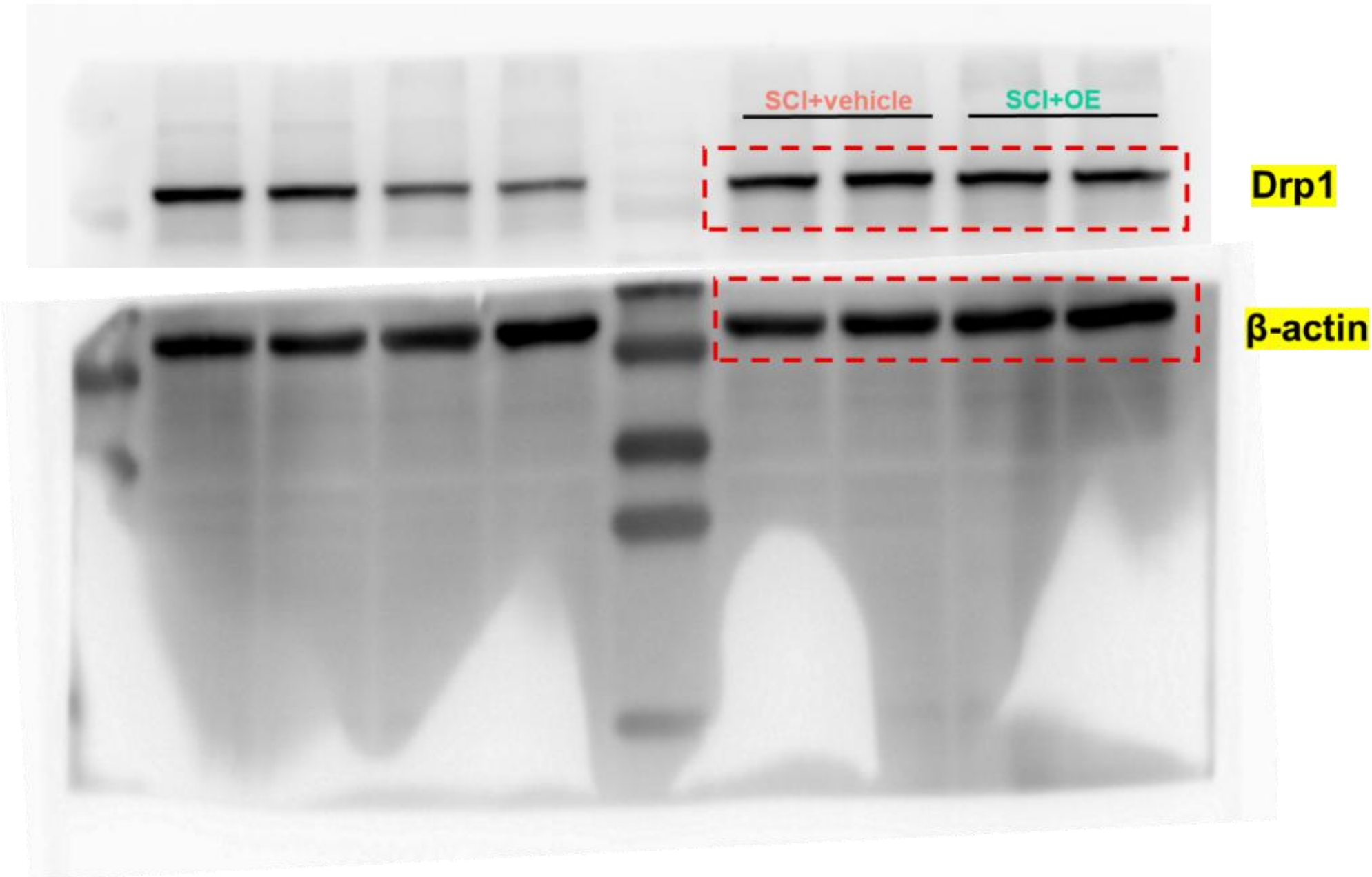

Full unedited blot/gel for Figure [7. r]

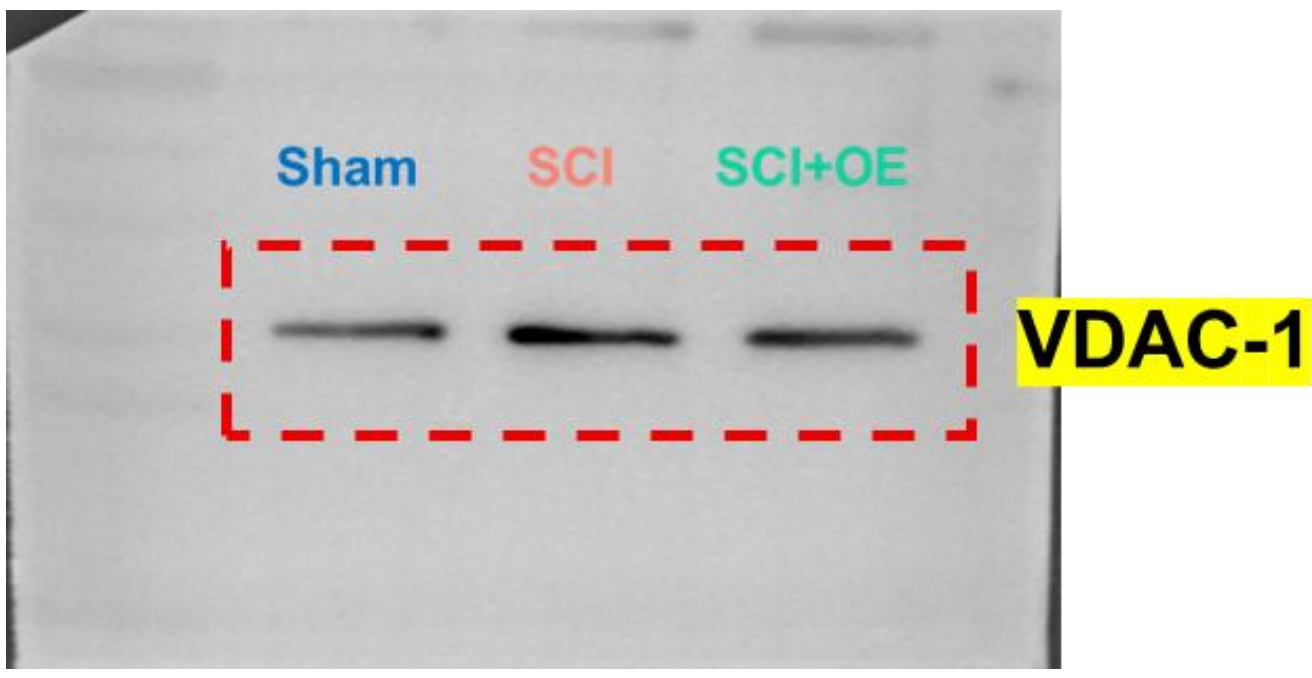

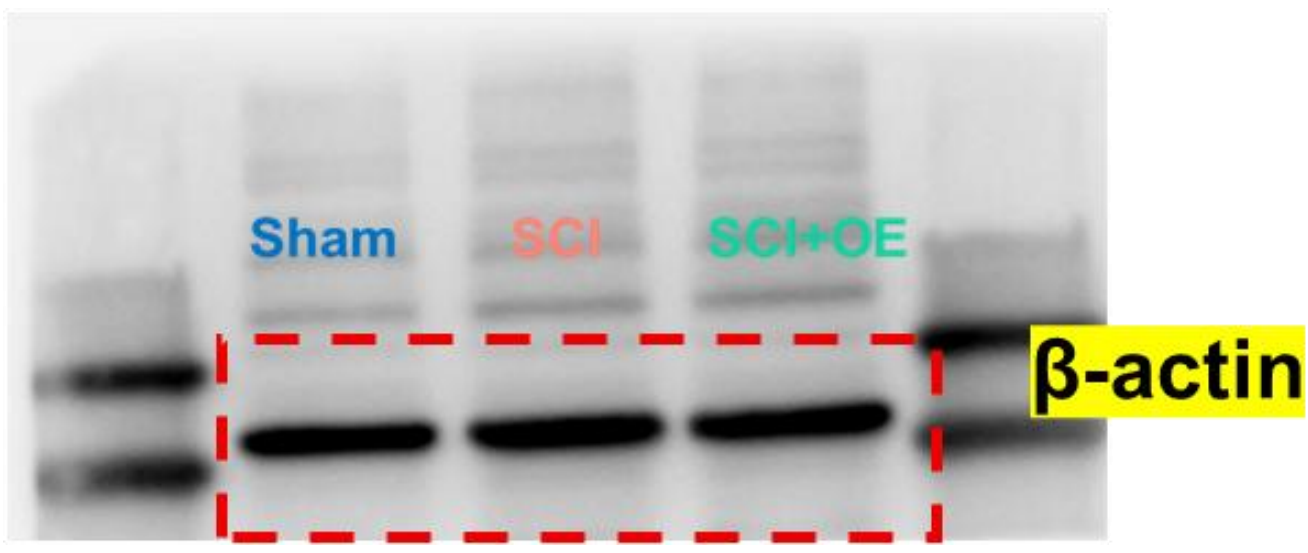

Full unedited blot/gel for Figure [7. s]

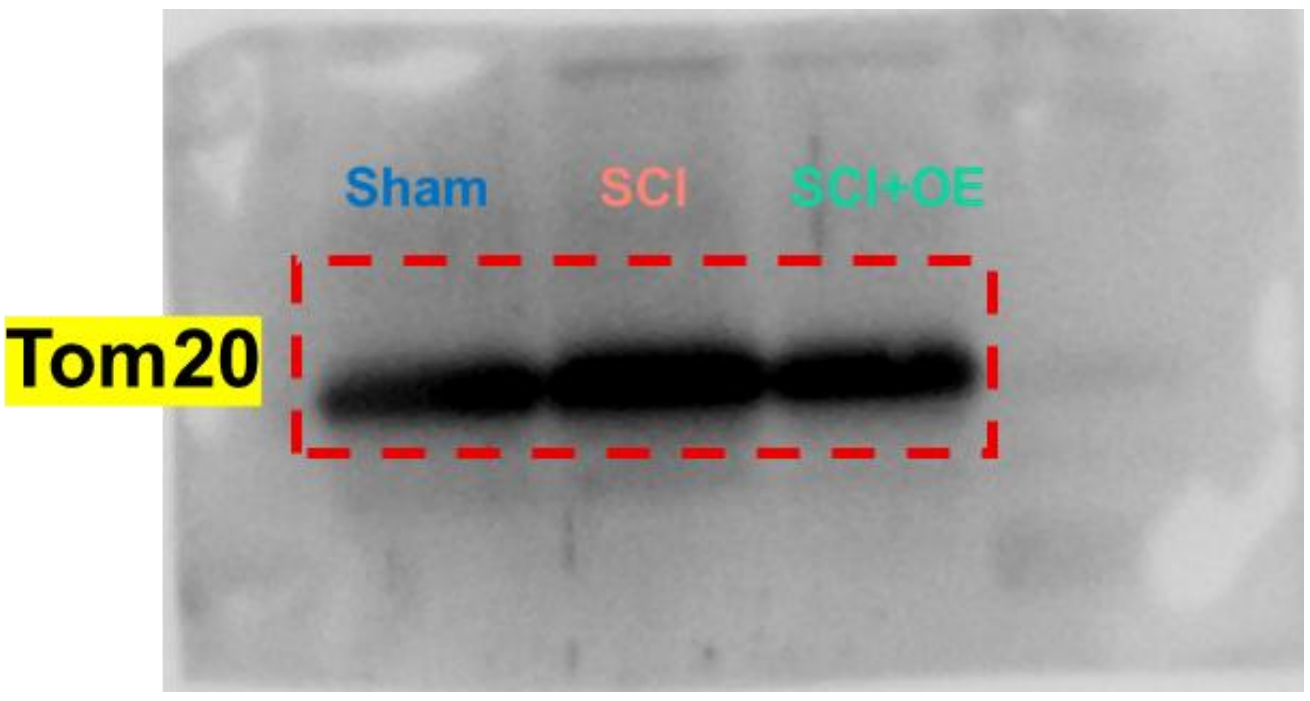
Sham
SCI
SCI+OE
Tom20

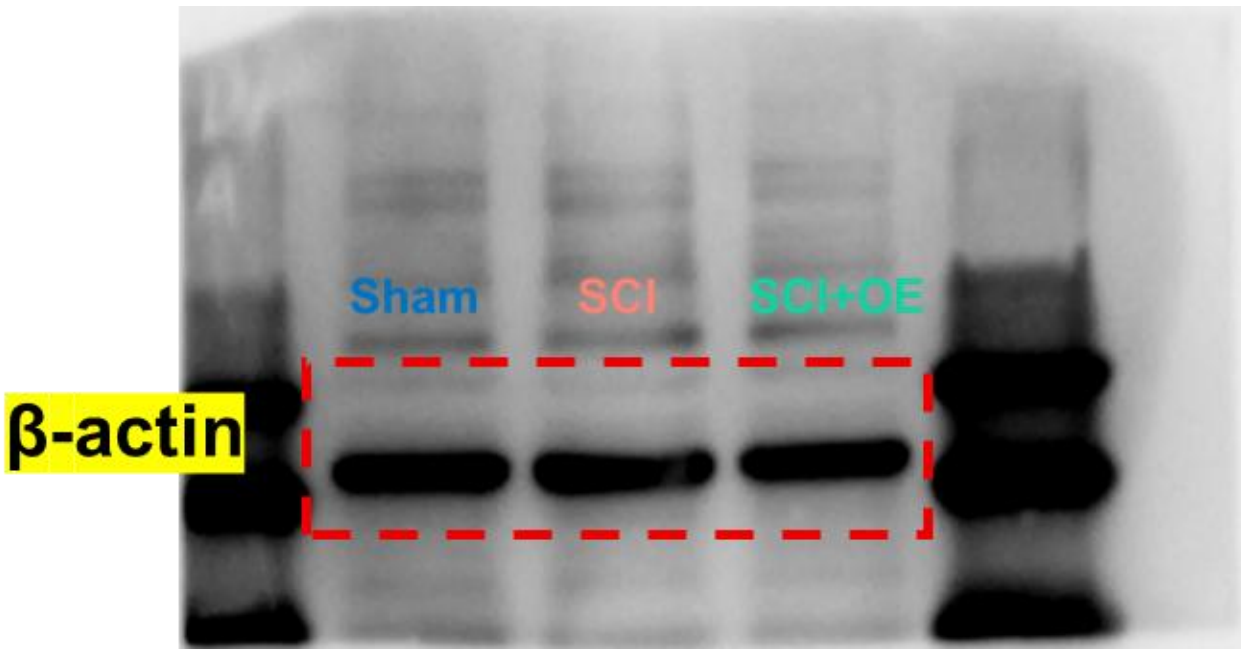
Sham
SCI
SCI+OE
β-actin

Full unedited blot/gel for Figure [8. b]

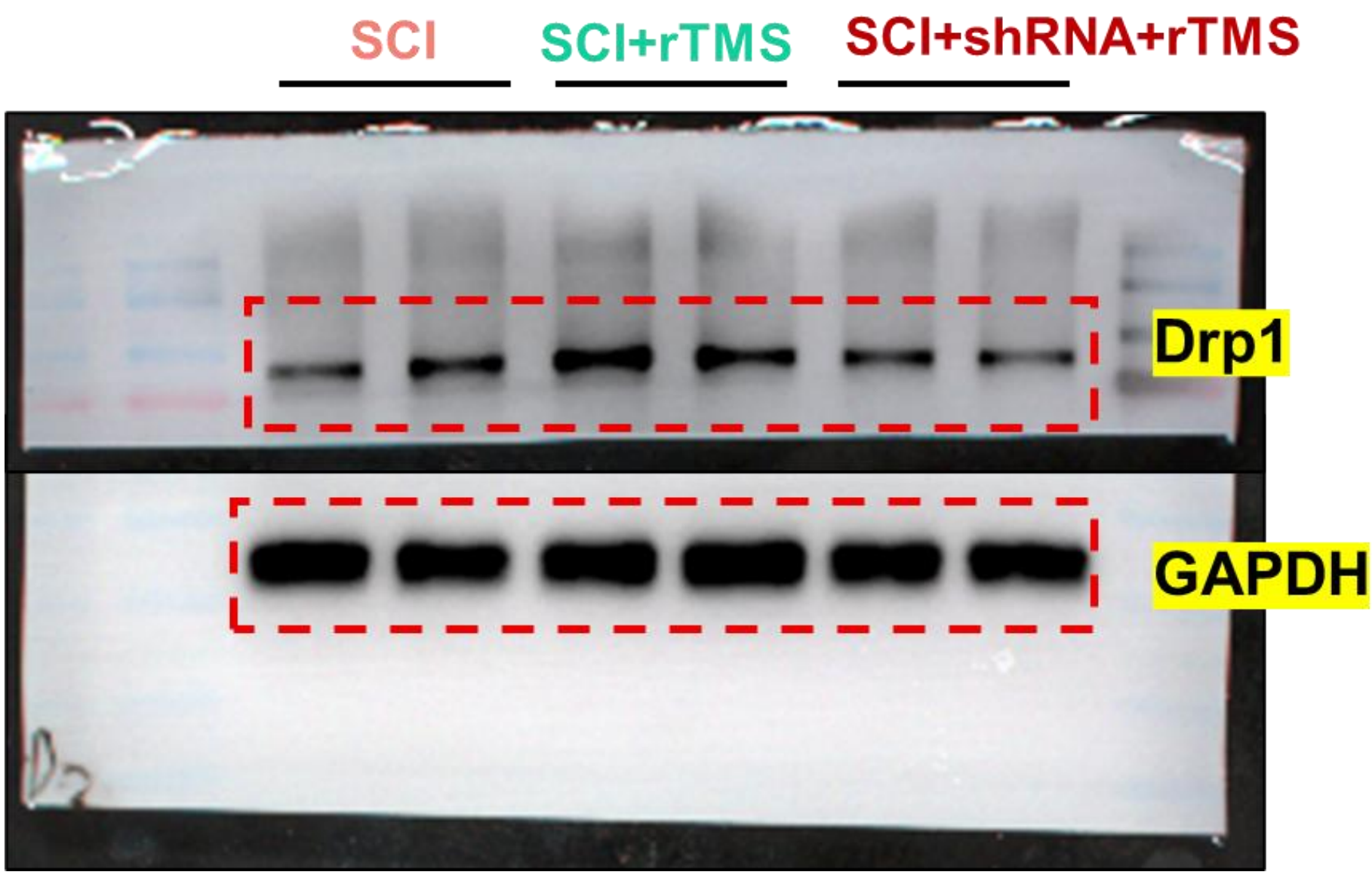

Full unedited blot/gel for Supplementary Figure [5. a]

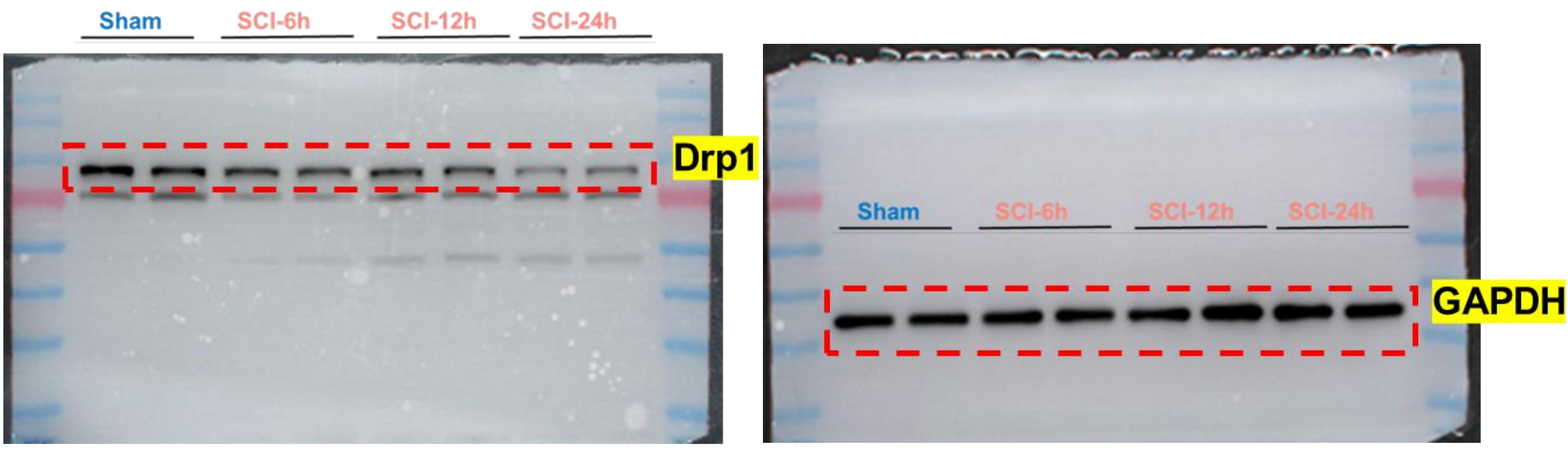

Full unedited blot/gel for Supplementary Figure [5. b]

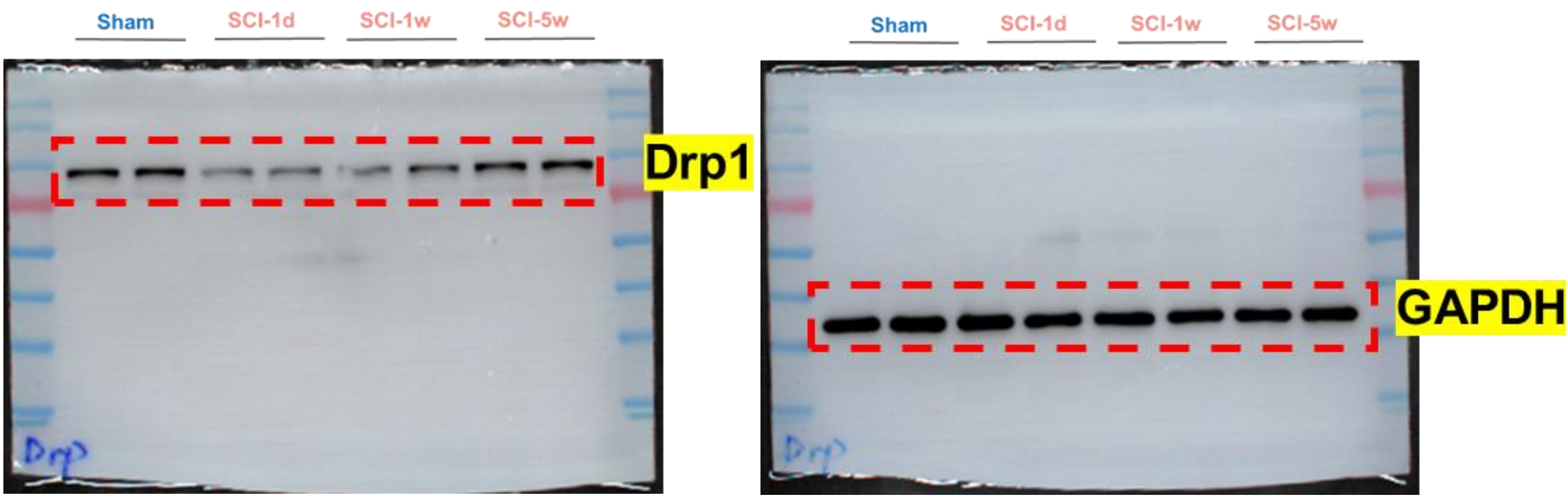

Full unedited blot/gel for Supplementary Figure [5. c]

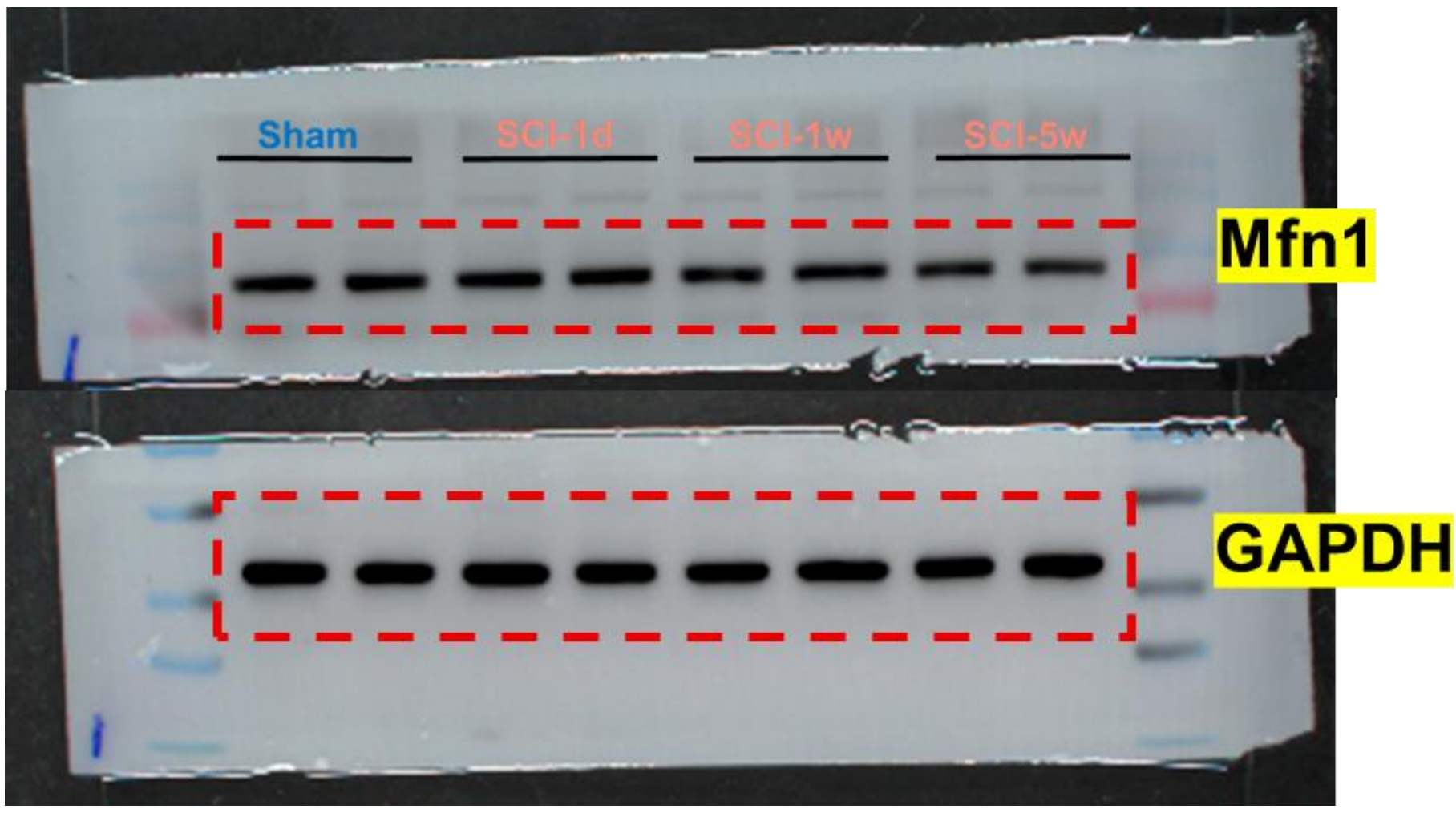

Full unedited blot/gel for Supplementary Figure [5. d]

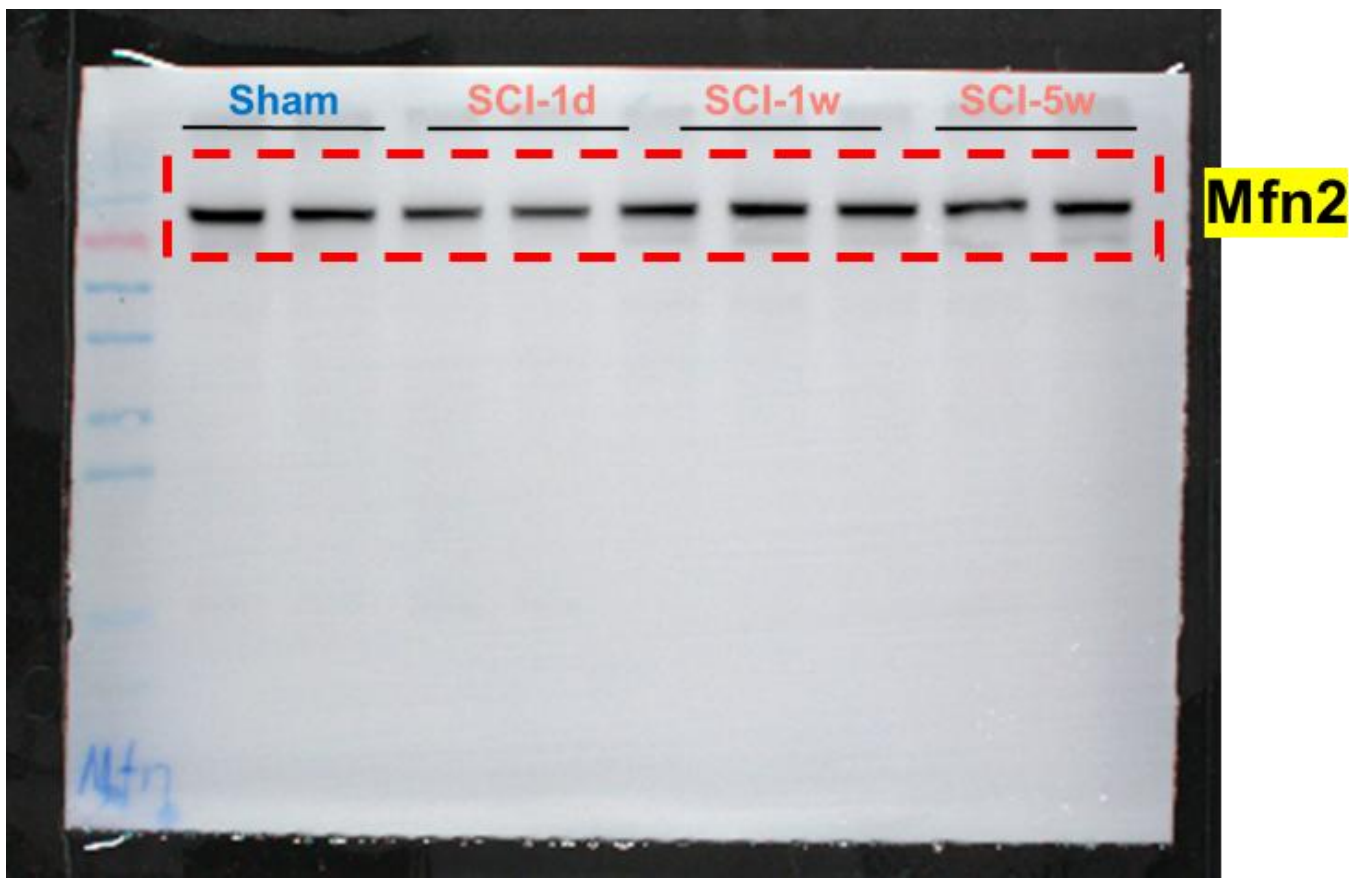

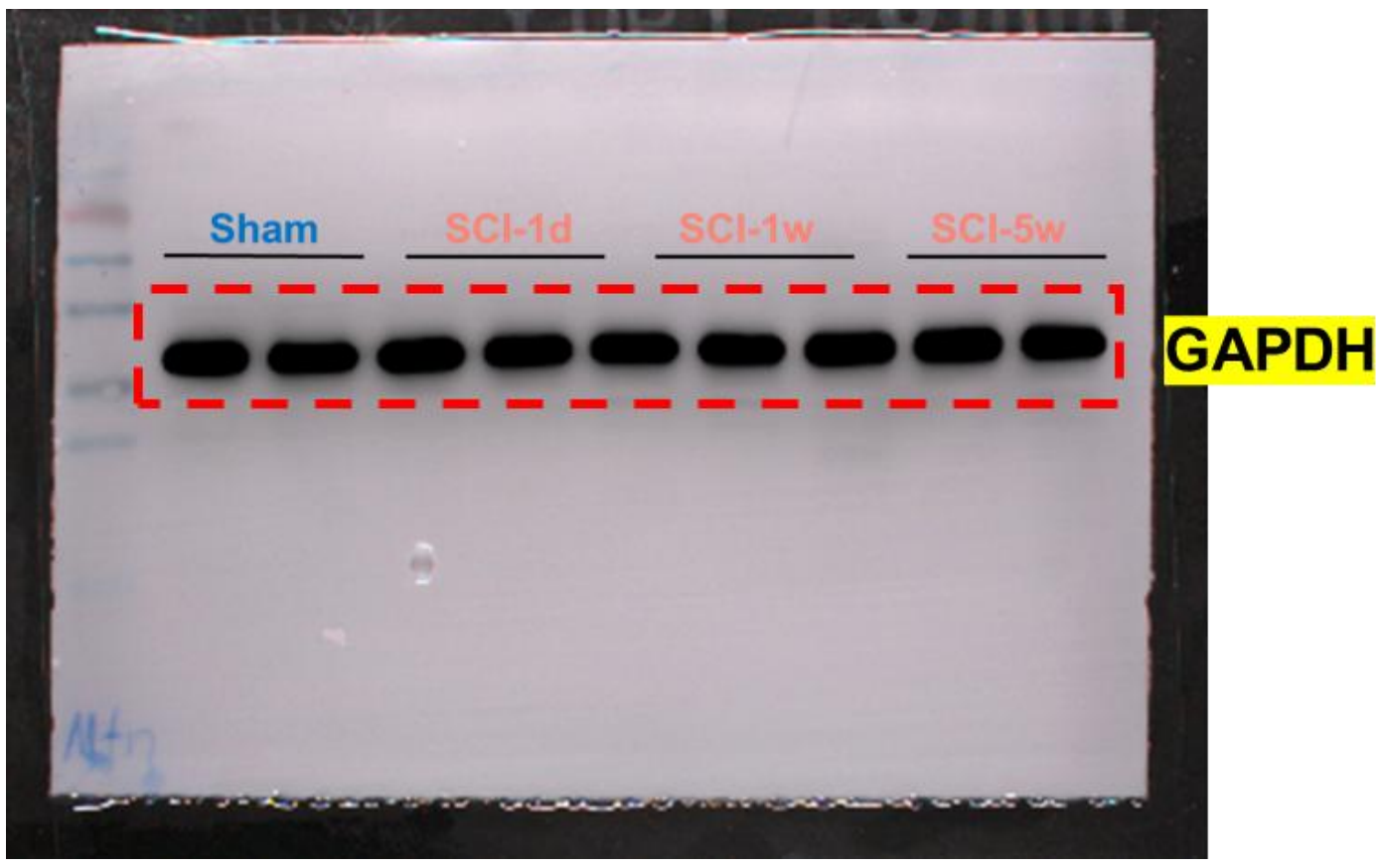

Full unedited blot/gel for Supplementary Figure [7. b]

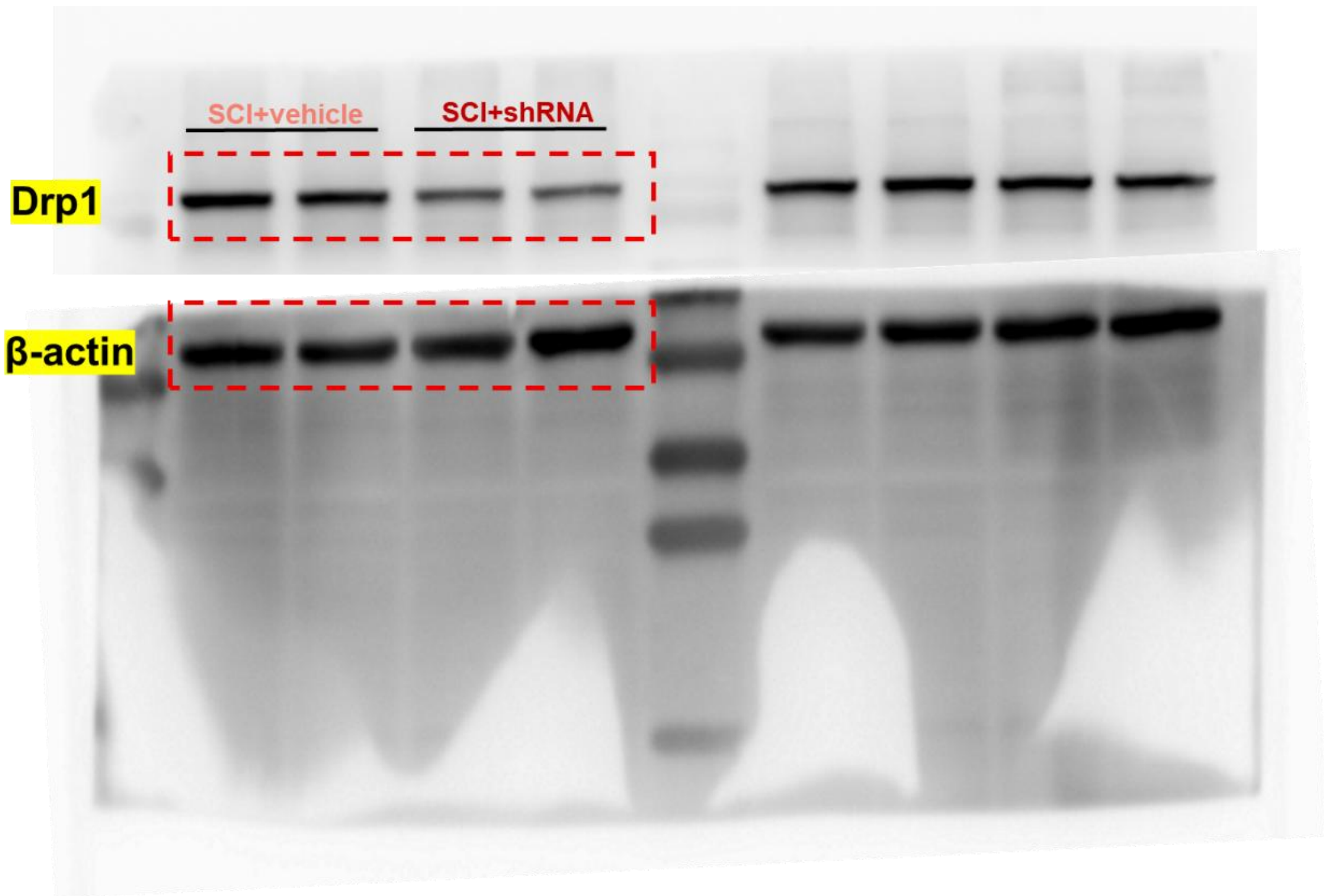

Full unedited blot/gel for Supplementary Figure [7. q]

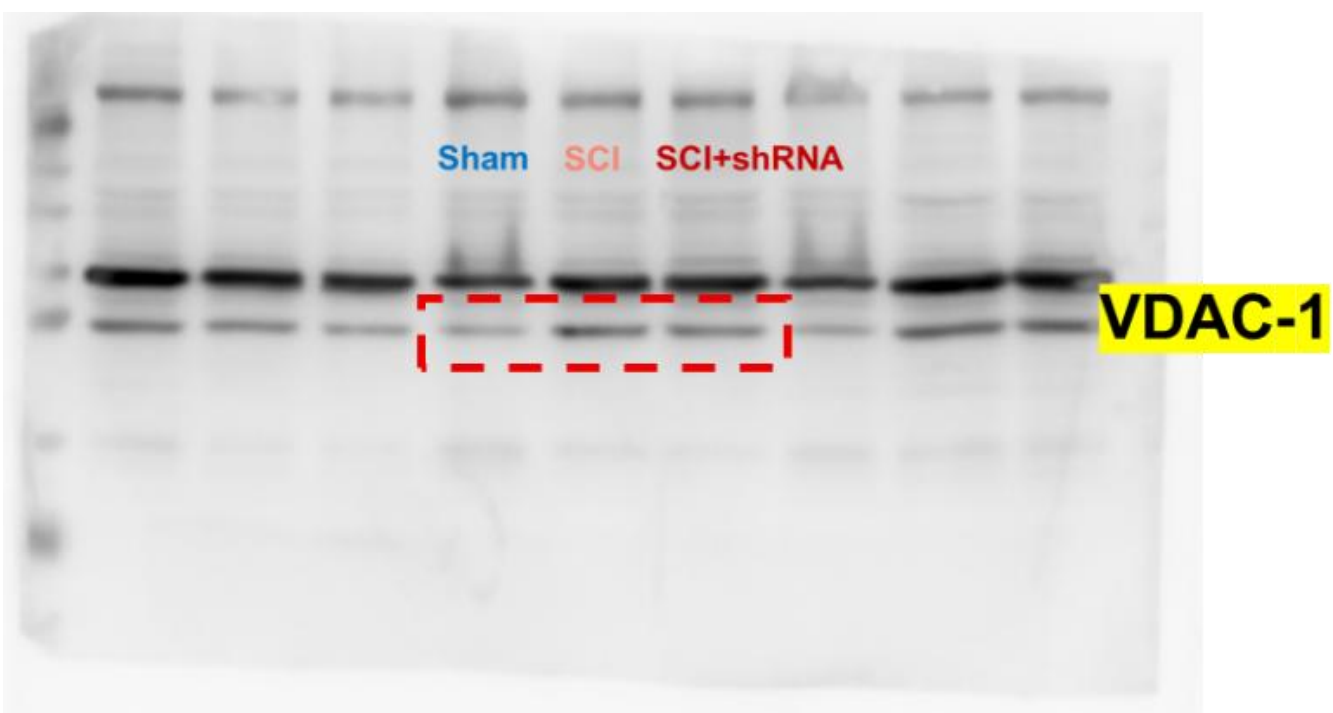

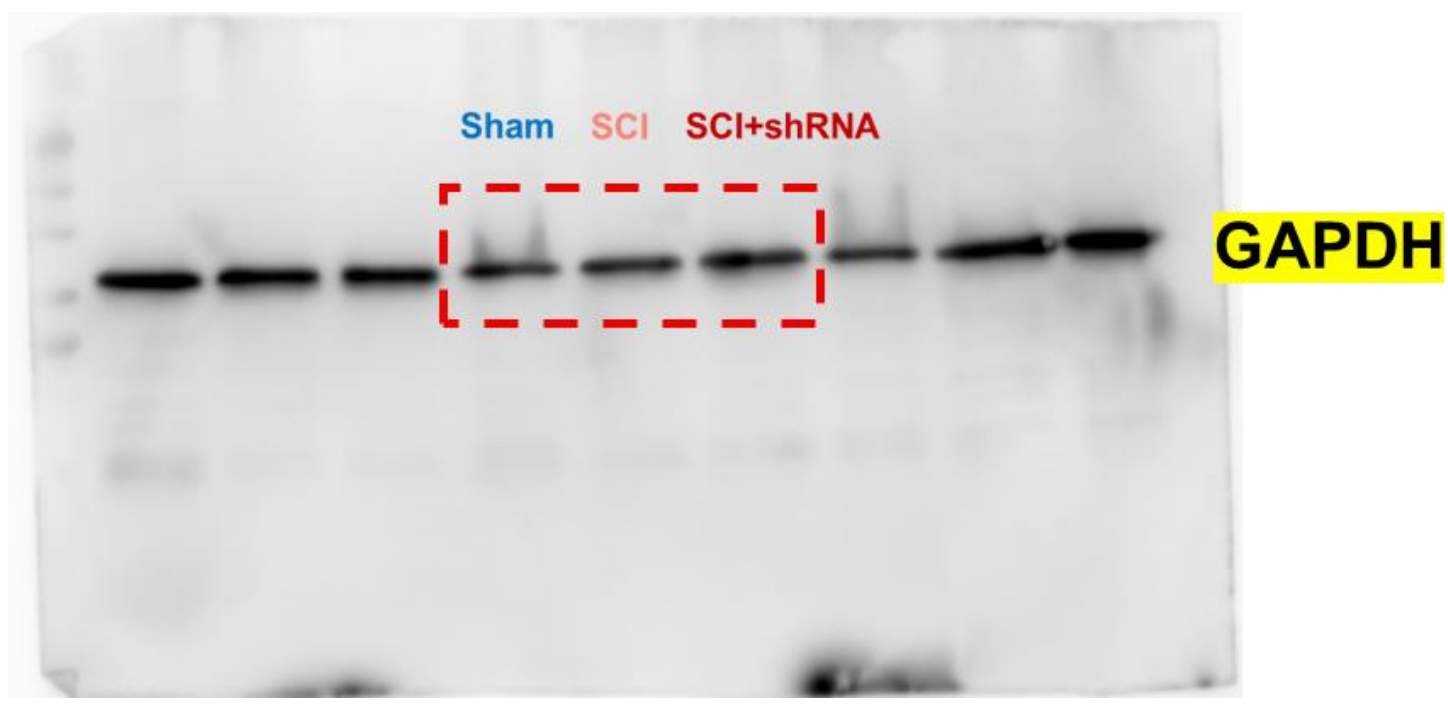

Full unedited blot/gel for Supplementary Figure [7. r]

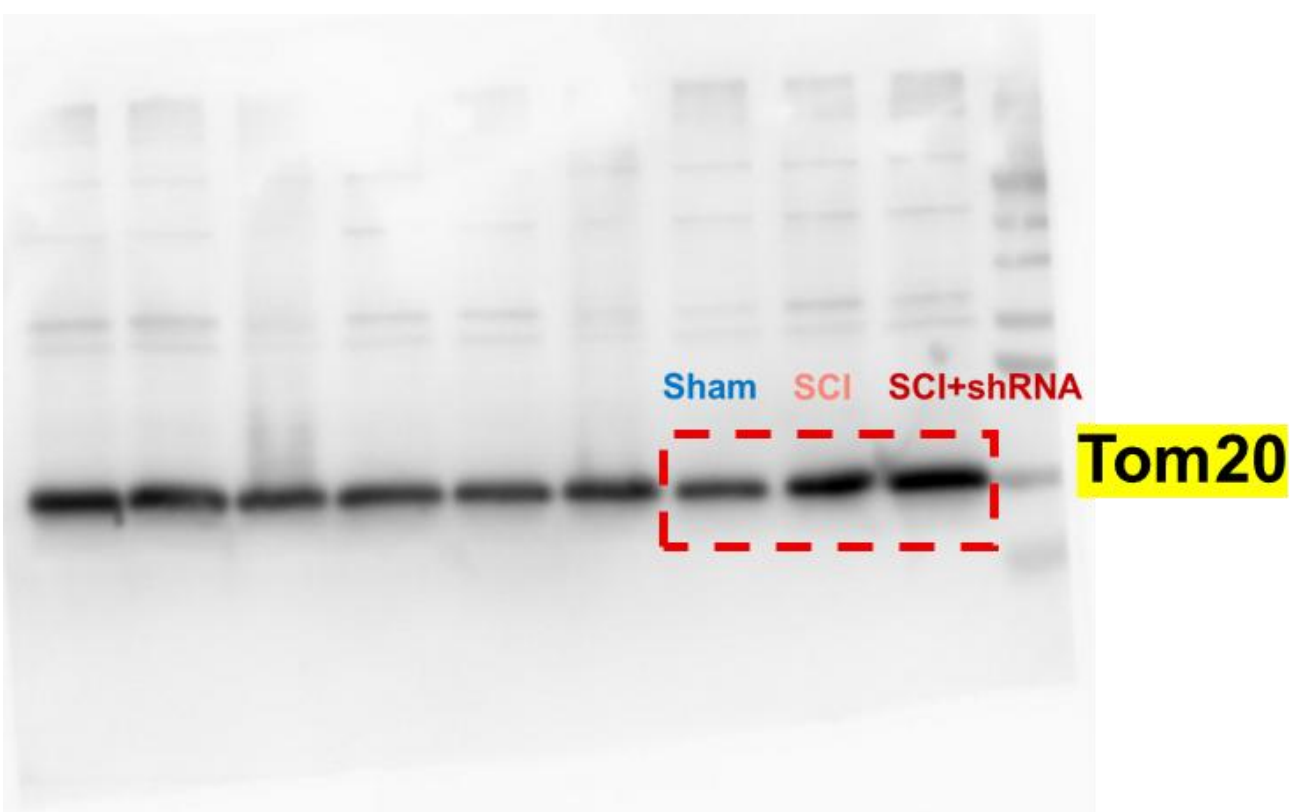

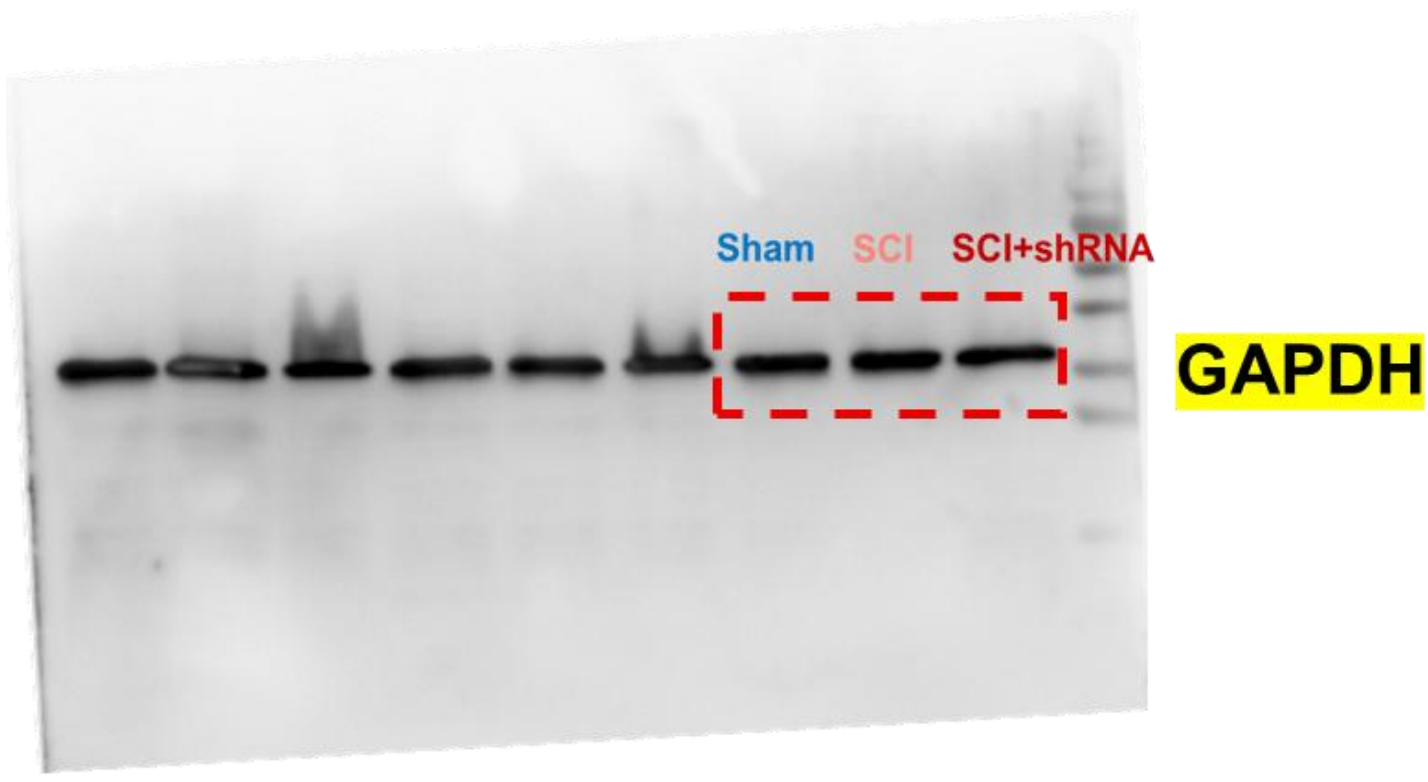

Full unedited blot/gel for Supplementary Figure [8. g]

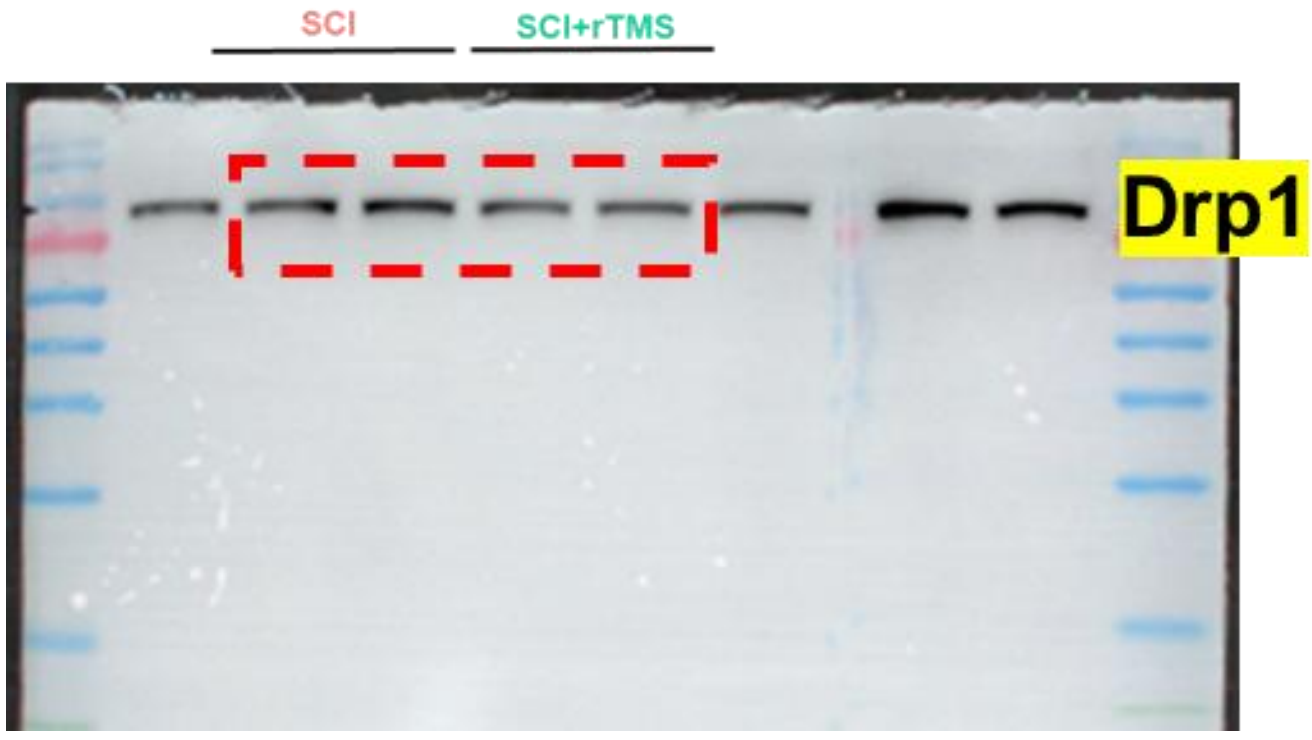

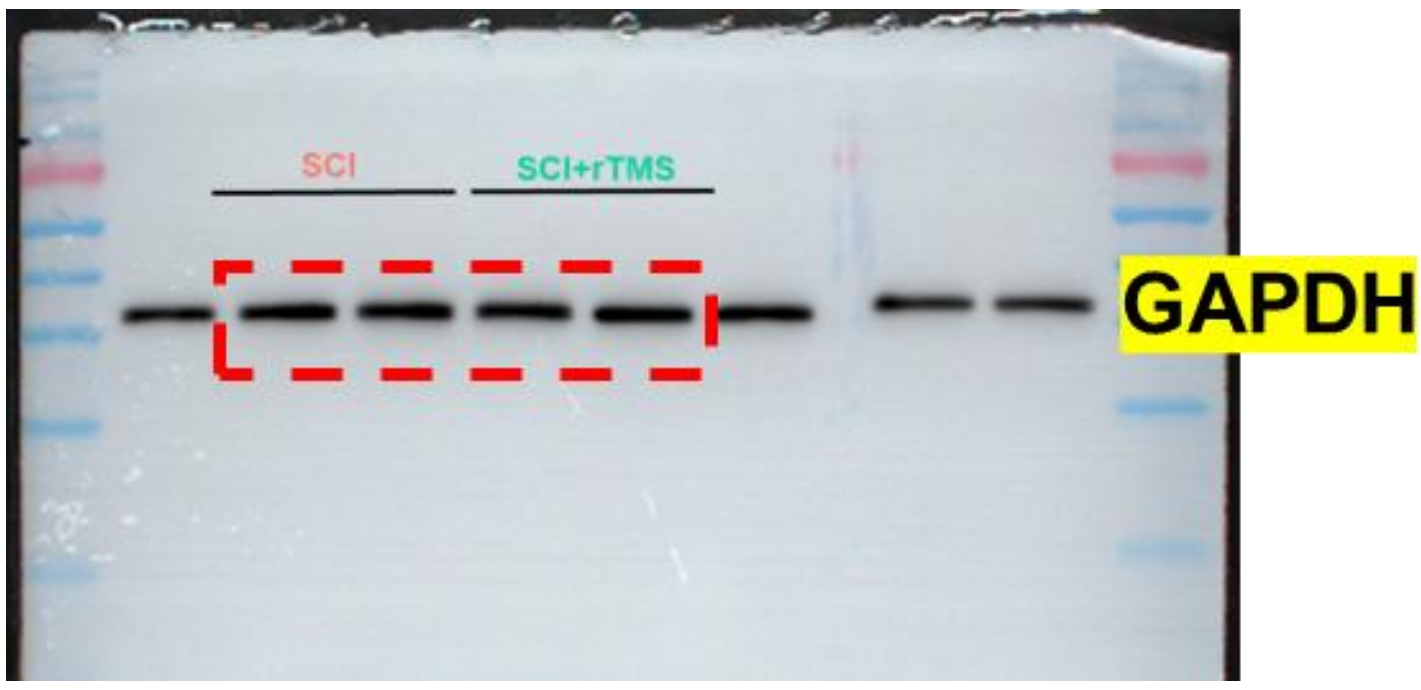

Supplement: Supplementary file 2 [file 12276_2026_1857_MOESM3_ESM.pdf]
