# Supplementary material for: Drp1 in M1 layer V GABAergic neurons orchestrates rTMS-mediated motor restoration and analgesia after spinal cord injury
Source: Exp Mol Med. 2026 Oct 5;58(10):3440–56. doi: 10.1038/s12276-026-01857-2 (PMC13737150; doi:10.1038/s12276-026-01857-2)
Supplement: Supplementary file 3 [file 12276_2026_1857_MOESM4_ESM.pdf]

脊髓损伤患者并发症、合并症、功能预后等相关因素回顾性临床研究

下载XML文档

注册号：<br>Registration number：	ChiCTR2300073824
最近更新日期：<br>Date of Last Refreshed on：	2023-09-14
注册时间：<br>Date of Registration：	2023-07-21
注册号状态：	补注册
Registration Status：	Retrospective registration
注册题目：	脊髓损伤患者并发症、合并症、功能预后等相关因素回顾性临床研究
Public title：	Retrospective clinical research protocol on complications, comorbidities, functional prognosis, and other related factors in patients with spinal cord injury
注册题目简写：	
English Acronym：	
研究课题的正式科学名称：	脊髓损伤患者并发症、合并症、功能预后等相关因素回顾性临床研究
Scientific title：	Retrospective clinical research protocol on complications, comorbidities, functional prognosis, and other related factors in patients with spinal cord injury
研究课题代号(代码)：<br>Study subject ID：	
在二级注册机构或其它机构的注册号：<br>The registration number of the Partner Registry or other register：	

申请注册联系人：	孙晓龙	研究负责人：	袁华
Applicant：	Sun Xiaolong	Study leader：	Yuan Hua
申请注册联系人电话：<br>Applicant telephone：	+86 159 2977 9672	研究负责人电话：<br>Study leader's telephone：	+86 136 5919 2988
申请注册联系人传真 ：<br>Applicant Fax：		研究负责人传真：<br>Study leader's fax：	
申请注册联系人电子邮件：<br>Applicant E-mail：	xlsun@fmmu.edu.cn	研究负责人电子邮件：<br>Study leader's E-mail：	yuanhua@fmmu.edu.cn
申请单位网址(自愿提供)：<br>Applicant website(voluntary supply)：		研究负责人网址(自愿提供)：<br>Study leader's website(voluntary supply)：	
申请注册联系人通讯地址：	陕西省西安市新城区长乐西路169号	研究负责人通讯地址：	陕西省西安市新城区长乐西路169号
Applicant address：	169 Changle Road West, Xincheng District, Xi'an, Shaanxi	Study leader's address：	169 Changle Road West, Xincheng District, Xi'an, Shaanxi
申请注册联系人邮政编码：<br>Applicant postcode：		研究负责人邮政编码：<br>Study leader's postcode：	
申请人所在单位：	空军军医大学第一附属医院		
Applicant's institution：	The First Affiliated Hospital of Air Force Medical University		
研究负责人所在单位：	空军军医大学第一附属医院		
Affiliation of the Leader：	The First Affiliated Hospital of Air Force Medical University		

是否获伦理委员会批准：	是		
Approved by ethic committee：	Yes		
伦理委员会批件文号：	KY20222096-C-1号	伦理委员会批件附件：	查看附件View

Approved No. of ethic committee:		Approved file of Ethical Committee:	
批准本研究的伦理委员会名称：	中国人民解放军空军军医大学第一附属医院医学伦理委员会		
Name of the ethic committee:	Medical Ethics Committee of the First Affiliated Hospital of Air Force Medical University of the People's Liberation Army		
伦理委员会批准日期：<br>Date of approved by ethic committee:	2022-04-27		
伦理委员会联系人：	贾艳艳		
Contact Name of the ethic committee:	Jia Yanyan		
伦理委员会联系地址：	陕西省西安市新城区长乐西路169号		
Contact Address of the ethic committee:	169 Changle Road West, Xincheng District, Xi'an, Shaanxi		
伦理委员会联系人电话：<br>Contact phone of the ethic committee:	+86 29 8477 1794	伦理委员会联系人邮箱：<br>Contact email of the ethic committee:	

研究实施负责（组长）单位：	空军军医大学第一附属医院
Primary sponsor:	The First Affiliated Hospital of Air Force Medical University
研究实施负责（组长）单位地址：	陕西省西安市新城区长乐西路169号
Primary sponsor's address:	169 Changle Road West, Xincheng District, Xi'an, Shaanxi

试验主办单位(项目批准或申办者)：
Secondary sponsor:

国家：	中国	省(直辖市)：	陕西	市(区县)：	西安
Country:	China	Province:	Shaanxi	City:	Xi'an
单位(医院)：	空军军医大学第一附属医院	具体地址：	陕西省西安市新城区长乐西路169号		
Institution hospital:	The First Affiliated Hospital of Air Force Medical University	Address:	169 Changle Road West, Xincheng District, Xi'an, Shaanxi		

经费或物资来源：	空军军医大学
Source(s) of funding:	Air Force Medical University
研究疾病：	脊髓损伤
Target disease:	spinal cord injury
研究疾病代码：	
Target disease code:	
研究类型：	观察性研究
Study type:	Observational study
研究所处阶段：	回顾性研究
Study phase:	Retrospective study
研究设计：	队列研究
Study design:	Cohort study
研究目的：	明确脊髓损伤患者并发症、合并症等相关危险因素及保护因素，功能预后的预测因子。明确脊髓损伤患者并发症、合并症的流行病学特点。
Objectives of Study:	To identify risk factors and protective factors related to complications and comorbidities in patients with spinal cord injury, as well as predictive factors for functional prognosis. To identify the epidemiological characteristics of complications and comorbidities in patients with spinal cord injury.
药物成份或治疗方案详述：	
Description for medicine or protocol of treatment in detail:	
纳入标准：	1.年龄≥18岁； 2.符合神经学分类国际标准中SCI的诊断标准。
Inclusion criteria	1. Age >= 18 years old; 2. Conforming to the diagnostic criteria of SCI in the international standards for neurological classification.
排除标准：	1.伴有严重心、肺、脑等重要脏器疾病患者； 2.临床资料不完整者。
Exclusion criteria:	1. Patients with severe heart, lung, brain and other important organ diseases; 2. Incomplete clinical data.

研究实施时间：<br>Study execute time:	从 From 2022-05-01至 To 2032-05-01	征募观察对象时间：<br>Recruiting time:	从 From 2022-05-01 至 To 2032-05-01

干预措施：
Interventions:

组别：	根据是否发生并发症、合并症分组	样本量：	2000
Group:	Grouping based on whether complications and comorbidities occurred	Sample size:	
干预措施：	无	干预措施代码：	
Intervention:	None	Intervention code:	

研究实施地点：<br>Countries of recruitment and research settings：	国家：	中国	省(直辖市)：	陕西	市(区县)：	西安
	Country：	China	Province：	Shaanxi	City：	Xi'an
	单位(医院)：	空军军医大学第一附属医院	单位级别：	三级甲等		
	Institution hospital：	The First Affiliated Hospital of Air Force Medical University	Level of the institution：	Tertiary A		

测量指标：<br>Outcomes：	指标中文名：	运动指数	指标类型：	主要指标
	Outcome：	total motor score	Type：	Primary indicator
	测量时间点：		测量方法：	
	Measure time point of outcome：		Measure method：	
	指标中文名：	ASIA分级	指标类型：	次要指标
	Outcome：	American Spinal Injury Association	Type：	Secondary indicator
	测量时间点：		测量方法：	
	Measure time point of outcome：		Measure method：	
	指标中文名：	SCIM评分	指标类型：	次要指标
	Outcome：	Spinal Cord Independence Measure	Type：	Secondary indicator
	测量时间点：		测量方法：	
	Measure time point of outcome：		Measure method：	
	指标中文名：	疼痛数字评分	指标类型：	次要指标
	Outcome：	numeric rating scales	Type：	Secondary indicator
	测量时间点：		测量方法：	
	Measure time point of outcome：		Measure method：	
	指标中文名：	导尿情况	指标类型：	次要指标
	Outcome：	Urinary catheterization condition	Type：	Secondary indicator
	测量时间点：		测量方法：	
	Measure time point of outcome：		Measure method：	
	指标中文名：	改良Barthel评分	指标类型：	次要指标
	Outcome：	Modified Barthel score	Type：	Secondary indicator
	测量时间点：		测量方法：	
	Measure time point of outcome：		Measure method：	
	指标中文名：	并发症情况	指标类型：	次要指标
	Outcome：	Complications	Type：	Secondary indicator
	测量时间点：		测量方法：	
	Measure time point of outcome：		Measure method：	
	指标中文名：	合并症情况	指标类型：	次要指标
	Outcome：	Comorbidities	Type：	Secondary indicator
	测量时间点：		测量方法：	
	Measure time point of outcome：		Measure method：	

采集人体标本：<br>Collecting sample(s) from participants：	标本中文名：	无	组织：	
	Sample Name：	None	Tissue：	
	人体标本去向	其它	说明	

Fate of sample:	Others	Note:	

征募研究对象情况：<br>Recruiting status：	正在进行<br>Recruiting	年龄范围：<br>Participant age：	最小 Min age 18 岁 years<br>最大 Max age 岁 years
性别：	男女均可	Gender：	Both

随机方法（请说明由何人用什么方法产生随机序列）：	不适用
Randomization Procedure (please state who generates the random number sequence and by what method)：	N/A
是否公开试验完成后的统计结果：<br>Calculated Results after the Study Completed public access：	公开/Public
盲法：	无
Blinding：	None
试验完成后的统计结果（上传文件）：	点击下载
Calculated Results after the Study Completed(upload file)：	download
是否共享原始数据：<br>IPD sharing	是Yes
共享原始数据的方式（说明：请填入公开原始数据日期和方式，如采用网络平台，需填该网络平台名称和网址）：	临床试验公共管理平台 (http://www.medresman.org.cn/login.aspx)
The way of sharing IPD"(include metadata and protocol, If use web-based public database, please provide the url)：	ResMan (http://www.medresman.org.cn/login.aspx)
数据采集和管理（说明：数据采集和管理由两部分组成，一为病例记录表(Case Record Form, CRF)，二为电子采集和管理系统(Electronic Data Capture, EDC)，如ResMan即为一种基于互联网的EDC：	病例记录表:评估表;电子采集和管理系统
Data collection and Management (A standard data collection and management system include a CRF and an electronic data capture：	Case Record Form: Evaluation Form; Electronic collection and management system.
数据与安全监察委员会：<br>Data and Safety Monitoring Committee：	有/Yes

返回列表

World Health Organization 香港临床试验注册中心

网站首页 | ChiCTR简介 | 检索入口 | 重要文件 | 注册指南 | 常见问题

中国临床试验注册中心 - 世界卫生组织国际临床试验注册平台一级注册机构

Copyright(c) (2005 - 2023) Chictr.org.cn.All rights reserved. 中国临床试验注册中心 版权所有

蜀ICP备16010396号-9

提示：推荐使用IE8.0以上版本 宽屏显示分辨率下使用系统
