# Supplementary material for: Drp1 in M1 layer V GABAergic neurons orchestrates rTMS-mediated motor restoration and analgesia after spinal cord injury
Source: Exp Mol Med. 2026 Oct 5;58(10):3440–56. doi: 10.1038/s12276-026-01857-2 (PMC13737150; doi:10.1038/s12276-026-01857-2)
Supplement: Supplementary file 4 [file 12276_2026_1857_MOESM5_ESM.pdf]

脊髓损伤后大脑双侧M1手区不同兴奋性干预方案对运动恢复影响的多中心随机对照研究

下载XML文档

注册号：<br>Registration number：	ChiCTR2400094593
最近更新日期：<br>Date of Last Refreshed on：	2025-12-03
注册时间：<br>Date of Registration：	2024-12-25
注册号状态：	预注册
Registration Status：	Prospective registration
注册题目：	脊髓损伤后大脑双侧M1手区不同兴奋性干预方案对运动恢复影响的多中心随机对照研究
Public title：	A multicenter randomized controlled study of the effects of different excitability intervention protocols in bilateral M1 hand regions of the brain on motor recovery after spinal cord injury
注册题目简写：	
English Acronym：	
研究课题的正式科学名称：	脊髓损伤后大脑双侧M1手区不同兴奋性干预方案对运动恢复影响的多中心随机对照研究
Scientific title：	A multicenter randomized controlled study of the effects of different excitability intervention protocols in bilateral M1 hand regions of the brain on motor recovery after spinal cord injury
研究课题代号(代码)：<br>Study subject ID：	
在二级注册机构或其它机构的注册号：<br>The registration number of the Partner Registry or other register：	

申请注册联系人：	张鑫	研究负责人：	袁华
Applicant：	Zhang Xin	Study leader：	Yuan Hua
申请注册联系人电话：<br>Applicant telephone：	+86 29 8477 5439	研究负责人电话：<br>Study leader's telephone：	+86 29 8477 5439
申请注册联系人传真 ：<br>Applicant Fax：		研究负责人传真：<br>Study leader's fax：	
申请注册联系人电子邮件：<br>Applicant E-mail：	445034571@qq.com	研究负责人电子邮件：<br>Study leader's E-mail：	445034571@qq.com
申请单位网址(自愿提供)：<br>Applicant website(voluntary supply)：		研究负责人网址(自愿提供)：<br>Study leader's website(voluntary supply)：	
申请注册联系人通讯地址：	西安市长乐西路127号	研究负责人通讯地址：	西安市长乐西路127号
Applicant address：	No. 127, Changle West Road, Xi'an	Study leader's address：	No. 127, Changle West Road, Xi'an
申请注册联系人邮政编码：<br>Applicant postcode：		研究负责人邮政编码：<br>Study leader's postcode：	
申请人所在单位：	空军军医大学第一附属医院		
Applicant's institution：	The First Affiliated Hospital of Air Force Medical University		
研究负责人所在单位：	空军军医大学第一附属医院		
Affiliation of the Leader：	The First Affiliated Hospital of Air Force Medical University		

是否获伦理委员会批准：	是		
Approved by ethic committee：	Yes		
伦理委员会批件文号：	KY20242248-F-1号; KY20242248-X-1号	伦理委员会批件附件：	查看附件View

Approved No. of ethic committee:	Approved file of Ethical Committee:
批准本研究的伦理委员会名称:	空军军医大学第一附属医院医学伦理委员会
Name of the ethic committee:	Medical Ethics Committee of the first Afficiated Hospital of the Air Force Medical University
伦理委员会批准日期:<br>Date of approved by ethic committee:	2024-08-16
伦理委员会联系人:	伍晓晓
Contact Name of the ethic committee:	Wu Xiaoxiao
伦理委员会联系地址:	西安市长乐西路127号
Contact Address of the ethic committee:	No. 127, Changle West Road, Xi'an
伦理委员会联系人电话:<br>Contact phone of the ethic committee:	+86 29 8477 1794
伦理委员会联系人邮箱:<br>Contact email of the ethic committee:	

研究实施负责（组长）单位:	空军军医大学第一附属医院
Primary sponsor:	The First Affiliated Hospital of Air Force Medical University
研究实施负责（组长）单位地址:	西安市长乐西路127号
Primary sponsor's address:	No. 127, Changle West Road, Xi'an

试验主办单位(项目批准或申办者):
Secondary sponsor:

国家:	中国	省(直辖市):	陕西省	市(区县):	西安市
Country:	China	Province:	Shaanxi Province	City:	Xi'an City
单位(医院):	空军军医大学第一附属医院	具体地址:	西安市长乐西路127号		
Institution hospital:	The First Affiliated Hospital of Air Force Medical University	Address:	No. 127, Changle West Road, Xi'an		

经费或物资来源:	国家自然科学基金
Source(s) of funding:	National Natural Science Foundation of China
研究疾病:	脊髓损伤
Target disease:	spinal cord injury
研究疾病代码:	
Target disease code:	
研究类型:	干预性研究
Study type:	Interventional study
研究所处阶段:	探索性研究/预试验
Study phase:	0
研究设计:	随机平行对照
Study design:	Parallel
研究目的:	比较不同方案的rTMS治疗脊髓损伤下肢运动功能障碍的疗效差异，探寻更有效的刺激方案，为rTMS治疗脊髓损伤下肢运动障碍提供临床指导
Objectives of Study:	To compare the differences in efficacy of different protocols of rTMS for the treatment of lower limb motor dysfunction in spinal cord injury, to explore more effective stimulation protocols, and to provide clinical guidance for the treatment of lower limb motor dysfunction in rTMS for spinal cord injury
药物成份或治疗方案详述:	方案1：优势侧10Hz rTMS+非优势侧1 Hz rTMS；方案2：优势侧10Hz rTMS+非优势侧sham rTMS；方案3：优势侧10 Hz rTMS+非优势侧10 Hz rTMS；方案4：sham rTMS+非优势侧sham rTMS
Description for medicine or protocol of treatment in detail:	Scheme 1: 10 Hz rTMS on dominant side + 1 Hz rTMS on non-dominant side; Scheme 2: 10 Hz rTMS on dominant side + sham rTMS on non-dominant side; Scheme 3: 10 Hz rTMS on dominant side + 10 Hz rTMS on non-dominant side; Scheme 4: sham rTMS + sham rTMS on non-dominant side
纳入标准:	1.年龄18-70岁，右利手（爱丁堡利手问卷）;<br>2.已确诊SCI患者;<br>3.受伤后病程： 6月以内;<br>4.患者2周内未进行任何形式的rTMS治疗;<br>5.无认知障碍，简易精神状态检查 (MMSE)评分≥24分;<br>6.患者或直系家属签署知情同意书。
Inclusion criteria	1. age 18-70 years, right-handed (Edinburgh Sharp Handedness Questionnaire);<br>2. patients with established SCI;<br>3. duration of illness after injury: less than 6 months;<br>4. patients not undergoing any form of rTMS treatment within 2 weeks;<br>5. no cognitive impairment with a score of >=24 on the Brief Mental State Examination (MMSE);<br>6. informed consent signed by the patient or immediate family.
排除标准:	1.严重神经系统疾病，不能耐受相关检测； 2.下肢骨折、动静脉瘘或其他导致下肢活动障碍的疾病； 3.头部或颈部有铁磁性植入物； 4.植入心脏起搏器； 5.严重凝血障碍、严重心功能不全或无法控制的高血压：收缩压>160 mmHg或舒张压>100 mmHg； 6.其他严重的全身性疾病如肿瘤等； 7.患者于1周内服用过巴氯芬、地西泮等影响spTMS检测的药物。

Exclusion criteria:	1. severe neurological disease that cannot tolerate the test; 2. lower limb fracture, arteriovenous fistula or other diseases that cause lower limb mobility impairment; 3. ferromagnetic implants in the head or neck; 4. implanted cardiac pacemakers; 5. severe coagulation disorders, severe cardiac insufficiency, or uncontrollable high blood pressure: systolic blood pressure >160 mmHg or diastolic blood pressure >100 mmHg; 6. other serious Systemic diseases such as tumors; 7. Patients who have taken medications that affect spTMS testing such as baclofen and diazepam within 1 week.
研究实施时间：<br>Study execute time：	从 From 2024-06-01至 To 2026-06-01
征募观察对象时间：<br>Recruiting time：	从 From 2025-01-01 至 To 2026-06-01

干预措施：
Interventions：

组别：	A组	样本量：	18
Group：	Group A	Sample size：	
干预措施：	优势侧10Hz rTMS+非优势侧1 Hz rTMS	干预措施代码：	
Intervention：	10 Hz rTMS on dominant side + 1 Hz rTMS on non-dominant side	Intervention code：	
组别：	B组	样本量：	18
Group：	Group B	Sample size：	
干预措施：	优势侧10Hz rTMS+非优势侧sham rTMS	干预措施代码：	
Intervention：	10Hz rTMS on dominant side + sham rTMS on non-dominant side	Intervention code：	
组别：	C组	样本量：	18
Group：	Group C	Sample size：	
干预措施：	优势侧10 Hz rTMS+非优势侧10 Hz rTMS	干预措施代码：	
Intervention：	10 Hz rTMS on dominant side + 10 Hz rTMS on non-dominant side	Intervention code：	
组别：	D组	样本量：	18
Group：	Group D	Sample size：	
干预措施：	sham rTMS+非优势侧sham rTMS	干预措施代码：	
Intervention：	sham rTMS + non-dominant side sham rTMS	Intervention code：	

研究实施地点：
Countries of recruitment and research settings：

国家：	中国	省(直辖市)：	陕西省	市(区县)：	西安市
Country：	China	Province：	Shaanxi	City：	Xi'an
单位(医院)：	空军军医大学第一附属医院	单位级别：	三甲		
Institution hospital：	The First Affiliated Hospital of Air Force Medical University	Level of the institution：	Tertiary A		
国家：	中国	省(直辖市)：	陕西省	市(区县)：	汉中市
Country：	China	Province：	Shaanxi	City：	Hanzhong
单位(医院)：	汉中市中心医院	单位级别：	三甲		
Institution hospital：	Hanzhong City Center Hospital	Level of the institution：	Tertiary A		
国家：	中国	省(直辖市)：	陕西省	市(区县)：	咸阳市
Country：	China	Province：	Shaanxi	City：	Xianyang
单位(医院)：	咸阳市中心医院	单位级别：	三甲		
Institution hospital：	Xianyang Center Hospital	Level of the institution：	Tertiary A		
国家：	中国	省(直辖市)：	陕西省	市(区县)：	西安市
Country：	China	Province：	Shaanxi	City：	Xi'an
单位(医院)：	西安市高新医院	单位级别：	三甲		
Institution hospital：	Xi'an Gaoxin Hospital	Level of the institution：	Tertiary A		
国家：	中国	省(直辖市)：	陕西省	市(区县)：	西安市
Country：	China	Province：	Shaanxi	City：	Xi'an

单位(医院)：	西安国际医学中心医院	单位级别：	三甲		
Institution hospital：	Xi'an International Medical Center Hospital	Level of the institution：	Tertiary A		
国家：	中国	省(直辖市)：	陕西省	市(区县)：	西安市
Country：	China	Province：	Shaanxi	City：	Xi'an
单位(医院)：	西安市第九医院	单位级别：	三甲		
Institution hospital：	Xi'an Ninth Hospital	Level of the institution：	Tertiary A		
国家：	中国	省(直辖市)：	陕西省	市(区县)：	西安市
Country：	China	Province：	Shaanxi	City：	Xi'an
单位(医院)：	西安市红会医院	单位级别：	三甲		
Institution hospital：	Xi'an Honghui Hospital	Level of the institution：	Tertiary A		
国家：	中国	省(直辖市)：	陕西省	市(区县)：	西安市
Country：	China	Province：	Shaanxi	City：	Xi'an
单位(医院)：	陕西省康复医院	单位级别：	三甲		
Institution hospital：	Shaanxi Rehabilitation Hospital	Level of the institution：	Tertiary A		

测量指标：
Outcomes：

指标中文名：	关键肌力	指标类型：	主要指标
Outcome：	Manual MuscleTest	Type：	Primary indicator
测量时间点：		测量方法：	
Measure time point of outcome：		Measure method：	
指标中文名：	改良Ashworth评分	指标类型：	次要指标
Outcome：	modified Ashworth scale	Type：	Secondary indicator
测量时间点：		测量方法：	
Measure time point of outcome：		Measure method：	
指标中文名：	10米步行测试	指标类型：	次要指标
Outcome：	10 Metre Walk Test	Type：	Secondary indicator
测量时间点：		测量方法：	
Measure time point of outcome：		Measure method：	
指标中文名：	脊髓损伤独立性测量	指标类型：	次要指标
Outcome：	spinal cord lesion independence measure	Type：	Secondary indicator
测量时间点：		测量方法：	
Measure time point of outcome：		Measure method：	
指标中文名：	改良Barthel评分	指标类型：	次要指标
Outcome：	Modified Barthel score	Type：	Secondary indicator
测量时间点：		测量方法：	
Measure time point of outcome：		Measure method：	
指标中文名：	简化McGill疼痛问卷	指标类型：	次要指标
Outcome：	Short-formMcGill Pain Questionnaire-2	Type：	Secondary indicator
测量时间点：		测量方法：	

Measure time point of outcome：		Measure method：	
指标中文名：	数字评分法	指标类型：	次要指标
Outcome：	numerical rating scale	Type：	Secondary indicator
测量时间点：		测量方法：	
Measure time point of outcome：		Measure method：	
指标中文名：	神经病理性疼痛评估量表	指标类型：	次要指标
Outcome：	Douleur Neuropathique 4 questions	Type：	Secondary indicator
测量时间点：		测量方法：	
Measure time point of outcome：		Measure method：	
指标中文名：	疼痛部位及范围	指标类型：	次要指标
Outcome：	Location and extent of pain	Type：	Secondary indicator
测量时间点：		测量方法：	
Measure time point of outcome：		Measure method：	
指标中文名：	焦虑自评量表	指标类型：	次要指标
Outcome：	Generalized Anxiety Disorder-7	Type：	Secondary indicator
测量时间点：		测量方法：	
Measure time point of outcome：		Measure method：	
指标中文名：	9项患者健康问卷	指标类型：	次要指标
Outcome：	Patient Health Questionnaire-9	Type：	Secondary indicator
测量时间点：		测量方法：	
Measure time point of outcome：		Measure method：	
指标中文名：	单脉冲TMS电生理检测	指标类型：	次要指标
Outcome：	Single-pulse Transcranial Magnetic Stimulation	Type：	Secondary indicator
测量时间点：		测量方法：	
Measure time point of outcome：		Measure method：	

采集人体标本：Collecting sample(s) from participants：				
	标本中文名：	无	组织：	
	Sample Name：	NONE	Tissue：	
	人体标本去向	**使用后销毁**	说明	
	Fate of sample：	Destruction after use	Note：	

征募研究对象情况：Recruiting status：	正在进行 Recruiting	年龄范围：Participant age：	最小 Min age 18 岁 years<br>最大 Max age 70 岁 years
性别：	男女均可	Gender：	Both
随机方法（请说明由何人用什么方法产生随机序列）：	由独立第三方通过随机软件生成随机数表		
Randomization Procedure (please state who generates the random number sequence and by what method)：	A table of random numbers is generated by an independent third party through random software		
是否公开试验完成后的统计结果：Calculated Results after the Study Completed public access：	公开/Public		
盲法：	单盲，对评估人员设盲。		
Blinding：	Single blind, blind evaluators.		
试验完成后的统计结果（上传文件）：	点击下载		
Calculated Results after the Study Completed(upload fil	download		

e)：	
是否共享原始数据：<br>IPD sharing	否No
共享原始数据的方式（说明：请填入公开原始数据日期和方式，如采用网络平台，需填该网络平台名称和网址）：	不共享原始数据
The way of sharing IPD"(include metadata and protocol, If use web-based public database, please provide the url)：	Do not share original data
数据采集和管理（说明：数据采集和管理由两部分组成，一为病例记录表（Case Record Form, CRF），二为电子采集和管理系统(Electronic Data Capture, EDC)，如ResMan即为一种基于互联网的EDC：	采用病历记录表进行数据管理
Data collection and Management (A standard data collection and management system include a CRF and an electronic data capture：	Case Record Form were used for data management
数据与安全监察委员会：<br>Data and Safety Monitoring Committee：	暂未确定/Not yet

返回列表

中国临床试验注册中心 - 世界卫生组织国际临床试验注册平台一级注册机构
Copyright(c) (2005 - 2023) Chictr.org.cn.All rights reserved. 中国临床试验注册中心 版权所有
蜀ICP备16010396号-9
提示：推荐使用IE8.0以上版本 宽屏显示分辨率下使用系统
